# Alteration of the Gut–Lung Axis After Severe COVID-19 Infection and Modulation Through Probiotics: A Randomized, Controlled Pilot Study

**DOI:** 10.3390/nu16223840

**Published:** 2024-11-08

**Authors:** Angela Horvath, Hansjörg Habisch, Barbara Prietl, Verena Pfeifer, Irina Balazs, Gabor Kovacs, Vasile Foris, Nikolaus John, Daniela Kleinschek, Nicole Feldbacher, Henning Grønbæk, Holger Jon Møller, Kristina Žukauskaitė, Tobias Madl, Vanessa Stadlbauer

**Affiliations:** 1 Center for Biomarker Research in Medicine (CBmed), Division of Translational Precision Medicine, Division of Precision Medicine Technologies, 8010 Graz, Austria; angela.horvath@cbmed.at (A.H.); barbara.prietl@cbmed.at (B.P.); verena.pfeifer@cbmed.at (V.P.); dr.irina.balazs@gmail.com (I.B.); nicole.feldbacher@cbmed.at (N.F.); 2 Division for Gastroenterology and Hepatology, Department of Internal Medicine, Medical University of Graz, 8010 Graz, Austria; kristina.zukauskaite@medunigraz.at; 3 Otto Loewi Research Center, Medicinal Chemistry, Medical University of Graz, 8010 Graz, Austria; hansjoerg.habisch@medunigraz.at (H.H.); tobias.madl@medunigraz.at (T.M.); 4 BioTechMed-Graz, 8010 Graz, Austria; 5 Division of Endocrinology and Diabetes, Department of Internal Medicine, Medical University of Graz, 8010 Graz, Austria; 6 Division of Pulmonology, Department of Internal Medicine, Medical University of Graz, 8010 Graz, Austria; gabor.kovacs@uniklinikum.kages.at (G.K.); vasile.foris@medunigraz.at (V.F.); nikolaus.john@medunigraz.at (N.J.); 7 Ludwig Boltzmann Institute for Lung Vascular Research, 8010 Graz, Austria; daniela.kleinschek@lvr.lbg.ac.at; 8 Departments of Hepatology and Gastroenterology, Aarhus University Hospital, 8200 Aarhus, Denmark; henning.gronbaek@aarhus.rm.dk; 9 Department of Clinical Medicine, Aarhus University, 8000 Aarhus, Denmark; holgmoel@rm.dk; 10 Department of Clinical Biochemistry, Aarhus University Hospital, 8200 Aarhus, Denmark; 11 Institute of Biosciences, Life Sciences Center, Vilnius University, 01513 Vilnius, Lithuania

**Keywords:** COVID-19, probiotics, gut microbiome, gut–lung axis, immune phenotyping, neutrophil function, quality of life

## Abstract

Background: The gut–lung axis could be a potential therapeutic target for improving post-acute COVID-19 symptoms, and probiotics have been proposed as possible modulators. Aim: We conducted a pilot study to understand alterations in the gut–lung axis and to explore the effects of a probiotic in post-acute COVID-19 disease. Methods: We included patients after severe COVID-19 disease (sCOV, n = 21) in a randomized, placebo-controlled trial to test the effect of a probiotic (Pro-Vi 5, Institute Allergosan, Graz, Austria) in a six-month intervention and used patients after mild disease (mCOV, n = 10) as controls, to compare the intestinal microbiome, metabolome, and patient-reported outcomes and biomarkers along the gut–lung axis at baseline and throughout probiotic intervention. Results: Compared to mCOV patients, sCOV patients showed lower microbial richness, which was significantly improved by probiotic intervention. A reorganization of Ruminococcaceae and Lachnospiraceae taxa was observed in sCOV patients but remained unaffected by the intervention. Serum metabolome showed a dysregulation of lipoproteins in accordance with higher BMI and comorbidities in sCOV patients. HDL and LDL fractions/components were temporarily decreased in the probiotic group. Stool metabolome was altered at baseline in sCOV patients and an increase in L-DOPA after 3 months and butyrate after 6 months of intervention could be observed. Probiotics partially improved reduced quality of life and modulated altered immune responses in sCOV patients. Increased intestinal permeability at baseline remained unaffected. Conclusion: The study provides evidence of long-term alterations of the gut–lung axis after severe COVID-19 infection and suggests that probiotics can modulate the biomarkers of the gut–lung axis.

## 1. Introduction

The outbreak of the novel coronavirus (SARS-CoV-2)-infected disease (COVID-19) began in December 2019, spread throughout China in early 2020, and developed as a pandemic thereafter, which has affected populations, societies, and lives considerably. Soon after the outbreak, it was reported that symptoms of the disease can persist for a relatively long time after viral clearance, suggesting the existence of a post-acute disease state [1]. Considerable disagreement about the definition and, thus, prevalence of post-acute COVID-19 exists, ranging from 10–87% of patients with COVID-19, depending on the time point of the study, the type of assessment, and the organ system studied [2,3]. This sometimes-dismissed syndrome in the early period of the pandemic is now recognized as a multi-organ disease, which reflects a significant healthcare challenge [4]. Post-acute COVID-19 typically involves different organ systems with pulmonary, cardiovascular, haematological, neuropsychiatric, endocrine, renal, gastrointestinal, and dermatological sequalae. Symptoms, including severe fatigue, cognitive dysfunction, or pain, lead to a considerable burden of disease and impairment of quality of life [4,5]. Post-acute COVID-19 sequelae are still less well understood compared to the pathophysiology of the acute disease. Viral immune perturbation and/or inflammatory tissue injury during the acute infection account for the post-acute COVID-19 syndrome.

COVID-19 infection is frequently characterized by a hyperinflammatory phenotype. The COVID-19 cytokine storm is characterised by rapid proliferation and hyperactivation of T cells, macrophages, mast cells, neutrophil granulocytes, and natural killer cells, and the overproduction of inflammatory cytokines and chemical mediators released by immune or nonimmune cells [6,7]. Early data also suggest that even if symptoms are only ‘mild to moderate’ during the acute infection, non-reversible lung damage develops in some patients. This may lead to long-term pulmonary complications for a subset of patients [8,9]. Growing evidence suggests a crosstalk between the gut microbiome and the lung, with the host immune system facilitating the communication between the microbiome, gut barrier, and the effector organ—termed the gut–lung axis. This gut–lung interaction may influence the COVID-19 severity in patients with extrapulmonary conditions [10,11]. The gut–lung axis as a link between dysbiosis, barrier dysfunction, translocation of bacterial products, and hyperinflammation has also been proposed as a potential therapeutic target [10,12,13]. Probiotics have been proposed to be a possible modulator of the disturbed gut–lung axis in COVID-19 disease and post-acute COVID-19 syndrome [14,15].

Most studies, however, focused on the relationship between the microbiome, the immune system, and outcome during the acute infection, and much less is known how the gut–lung axis recovers after mild and after severe infections and how this could be associated with long-term sequelae of the disease [16]. To that end, we assessed a large set of clinical, immune system, and microbiome/metabolome-related biomarkers in patients after mild and after severe COVID-19 disease and performed a randomized, double-blind, controlled pilot trial with a probiotic mixture in patients who previously suffered from severe COVID-19 disease. The main aim was to explore which biomarkers of the gut–lung axis are altered in patients after mild versus severe COVID-19 infection and which of these biomarkers can be modulated by a probiotic.

## 2. Materials and Methods

### 2.1. Clinical Study

Patients after severe COVID-19 infection within the last 12 months, were defined as having experienced one or more of the following: hospitalization, need for oxygen supply, need for intensive care treatment, need for specific treatment of COVID-19 disease, or antibiotic treatment, and were recruited for this study. Patients were included between 2 June 2021 and 17 July 2022 when they gave written informed consent, and were 18 years old or older. Patients with probiotic treatment in the last 4 weeks before inclusion and those with pre-existing lung diseases were excluded. Patients after COVID-19 infection with a mild disease course (meeting none of the above criteria) and no residual symptoms of COVID-19 disease were assigned as controls. The study was conducted according to the ethical standard of the Declaration of Helsinki of 2013, approved by the research ethics board of the Medical University of Graz (33-302 ex 20/21) (15 April 2021) and registered at clinicaltrials.gov (NCT04813718). After fulfilling all inclusion criteria and none of the exclusion criteria, patients from the group with severe COVID-19 infection were randomized into 2 groups in a 1:1 ratio. Group 1 received a probiotic mixture (OMNi-BiOTiC^®^ Pro-Vi 5, Institut Allergosan, Graz, Austria) twice a day, and group 2 received a similar looking and tasting placebo twice a day. Participants were randomized in blocks of four and allocated to each group with the software Sealed Envelope Ltd. 2022 (available from: https://www.sealedenvelope.com/simple-randomiser/v1/lists [accessed on 11 July 2023]). The bacterial strains in OMNi-BiOTiC^®^ Pro-Vi 5 are *Lactobacillus delbrueckii* ssp. *bulgaricus* LB2 (LMG P-21905), *Lactobacillus rhamnosus* SP1 (DSM 21690), *Lactobacillus reuteri* DSM 12246, *Lactobacillus rhamnosus* CRL1505 (DSM 29673), and *Bifidobacterium animalis ssp. lactis* DSM 15954. The matrix consists of maltodextrin, hydrolysed potato starch, inulin, and vitamin D (5 µg cholecalciferol). One sachet contains 5 × 10^9^ CFU (2 g). The placebo consists of the matrix without bacterial strains. The study product is licensed as a food for special medical purposes as OMNi-BiOTiC^®^ Pro-Vi 5. All probiotic strains used in this study have “qualified presumption of safety” status. Full blood count, inflammation, kidney function, liver function, electrolytes, glucose metabolism, lipid metabolism were assessed using standard methods at the central laboratory facility of the Medical University of Graz, Graz, Austria. A complete lung function test (spirometry, whole-body plethysmography, diffusion capacity for CO (DLCO) and NO (DLNO)), capillary blood gas analysis from the hyperaemized earlobe, and a 6 min walk test (6MWT) were performed at the Division of Pulmonology of the Medical University of Graz, Graz, Austria by trained personnel.

### 2.2. Quality of Life

Quality of life was assessed by the short form (SF) 36 questionnaire for health-related quality of life [17], the gastrointestinal quality of life index (GIQLI) [18], and the acute respiratory tract infection questionnaire (ARTIQ) [19]. Analysis and formation of items und sum scores were conducted according to the authors’ suggestions. To grade post-acute COVID-19 outcome, the recovery was graded by the recovery scale for COVID-19 and the respiratory functioning, symptoms, and conditions were assessed by the modified Medical Research Council Dyspnea Scale (mMRC), as suggested in the PC-COS consensus [20] (and the unpublished draft consensus https://www.pc-cos.org/pc-cos_results, accessed on 13 October 2022) retrospectively on the dataset.

### 2.3. Microbiome Sequencing

Stool samples were collected in DNA/RNA shield (ZymoResearch, Irvine, CA, USA) either on the day of the study visit or the day before, stored at room temperature until the study visit, and then frozen at −80 °C until further analysis. For microbiome analysis, samples were thawed, and DNA was extracted using the QIAamp FAST DNA stool mini kit (QIAGEN, Hilden, Germany) automated using the QIAcube (QIAGEN, Hilden, Germany). Approximately 200 mg stool were transferred to 0.70 mm garnet bead tubes filled with 1 mL InhibitEx buffer (QIAGEN, Hilden, Germany). Subsequently, bead beating was performed using a SpeedMill PLUS (Analytik Jena, Jena, Germany) for 45 s at 60 Hz. Samples were then heated to 95 °C for 5 min and centrifuged for 1 min at 10.000 rpm. The resulting supernatant was transferred to a 1.5 mL microcentrifuge tube, which was placed in the QIAcube for follow-up automated DNA isolation according to the manufacturer’s instructions. Isolated DNA was used to amplify the hypervariable regions V1–V2 of the *16S rRNA* gene using the primer pair 27F-338R in a dual-barcoding approach according to Caporaso et al. [21]. A total of 3 µg of DNA were used for amplification, and PCR products were verified by electrophoresis in agarose gel. PCR products were normalized using the SequalPrep Normalization Plate Kit (ThermoFisherScientific, Waltham, MA, USA) pooled equimolarly and sequenced on the Illumina MiSeq v3 2 × 300 bp (Illumina Inc., San Diego, CA, USA). Demultiplexing was based on 0 mismatches in the barcode sequences. The resulting sequences were pre-processed on a local Galaxy instance (https://galaxy.medunigraz.at, accessed on 21 December 2022) using QIIME2 tools; amplicon sequencing variants (ASV) were identified with the dada2 inference algorithm, while taxonomic assignment was performed with a naïve Bayesian classifier trained on the SILVA database V132 with release at 99% identity. Cyanobacteria were regarded as contaminations and excluded from further analysis.

### 2.4. Gut Permeability and Bacterial Translocation Analysis Methods

Zonulin (Immundiagnostik, Bensheim, Germany), soluble CD14 (R&D Systems, Minneapolis, MN, USA), and LPS (Hycult Biotech, Uden, The Netherlands) were measured in serum by using an enzyme-linked immunosorbent assay (ELISA) according to the manufacturer’s instructions.

### 2.5. NMR Metabolomics

Lipoproteins, small molecular metabolites, and glycoproteins of human serum samples were measured on a Bruker 600 MHz Avance Neo NMR spectrometer (Bruker, Rheinstetten, Germany). Among the measured lipoproteins are very low density lipoprotein (VLDL), intermediate density lipoprotein (IDL), low density lipoprotein (LDL), and high density lipoprotein (HDL) subclasses, including their main apolipoprotein and lipid/cholesterol constituents (a total of 112 parameters). Small metabolites (a total of 41 parameters) include amino acids and their derivatives, carboxylic acids, ketone bodies, and monosaccharides. Serum samples were thawed, and 330 µL of each sample were mixed with 330 µL of Bruker serum buffer (Bruker, Rheinstetten, Germany). Following gentle mixing, 600 µL of the samples were transferred into 5 mm glass tubes and placed into a SampleJet rack (Bruker, Rheinstetten, Germany). Proton spectra were obtained at a constant temperature of 310 K using a standard nuclear Overhauser effect spectroscopy (NOESY) pulse sequence (Bruker: noesygppr1d), a Carr–Purcell–Meiboom–Gill (CPMG) pulse sequence [22] with pre-saturation during the relaxation delay (Bruker: cpmgpr1d) to achieve water suppression, and a fast scan 2D J-resolved (JRES) pulse sequence (Bruker: jresgpprqf) [23]. For resolving N-acetyl- and choline moieties of glycoproteins and other inflammation related proteins, J-edited diffusional 1H-NMR spectra (JEDI or PGPE, respectively) were recorded [24]. Raw data analysis was carried out using the Bruker IVDr Plasma (B.I.) module (for the quantification of lipoproteins and small molecular metabolites) [25], and the PhenoRisk PACS™ RuO module was used for quantifying glycoproteins [26] in the analysis software (Topspin version 4.1, Bruker). Fecal samples for NMR spectroscopy measurements were prepared as previously described [27]. Briefly, 200 µL of samples (50 to 100 mg of stool in 2 mL of DNA/RNA shield) were mixed with 400 µL methanol to inactivate and precipitate proteins. The remaining solids were lysed using a Precellys homogenizer (Bertin Technologies SAS, Montigny-le-Bretonneux, France) and stored at −20 °C for 1 h, followed by centrifugation for 30 min at 10,000× *g* at 4 °C. Finally, the supernatants were lyophilized, resuspended in 500 μL NMR buffer (0.08 M Na_2_HPO_4_, 5 mM 3-(trimethylsilyl) propionic acid-2,2,3,3-d4 sodium salt (TSP), and 0.04 (*w*/*v*) % NaN_3_ in D_2_O, with the pH adjusted to 7.4 with HCl or NaOH, respectively), and transferred into 5 mm NMR tubes for measurement on the NMR instrument using the CPMG pulse sequence as described. Spectra pre-processing and data analysis were carried out using Matlab^®^ scripts (courtesy of Prof. Jeremy Nicholson, Imperial College London, London, UK). NMR data were imported to Matlab^®^ vR2014b (Mathworks, Natick, MA, USA), with the regions around the water, TSP, and remaining methanol signals excluded, aligned, and corrected for the sample metabolite dilution by probabilistic quotient normalization [28]. The reported values correspond to an arbitrary unit (A.U.) derived from the area under the peak being proportional to concentration.

### 2.6. Innate Immunity

The soluble hemoglobin receptor (sCD163) and soluble mannose receptor (sMR) were analyzed as macrophage activation markers; sCD163 and sMR were measured in plasma samples by in-house sandwich ELISAs using a BEP-2000 ELISA-analyzer (Dade Behring), as previously described [29,30].

Neutrophils were isolated from human peripheral venous blood as previously described [31]. Neutrophils were counted with either a TC20TM Automated Cell Counter (Bio-Rad, Hercules, CA, USA) or a Neubauer chamber, and their viability was determined with trypan blue exclusion staining. The method yields a neutrophil purity of >95% according to Diff-Quik (Thermo Scientific)-stained cytocentrifuge preparations. ROS production was measured indirectly using chemiluminescence in 5 × 10^5^ freshly isolated neutrophils per well at 37 °C in luminescence-grade 96-well plates (Greiner Bio-One, Kremsmünster, Austria) in a Lumistar Omega luminescence microplate reader (BMG Labtech, Offenburg, Germany), as previously described [31,32]. To investigate neutrophil chemotaxis, µ-slide chemotaxis chambers (ibidi, Martinsried, Planegg, Germany) were used. Freshly isolated human neutrophils were mixed with collagen from rat tails (Roche, Basel, Switzerland), sodium bicarbonate buffer, 10x HBSS with Ca^2+^ and Mg^2+^, and phenol red. The suspension was added to the capillary of µ-slide and removed with the pipette from another edge of capillary. Slides were incubated for 20 min at 37 °C until the collagen solidified. HBSS buffer with or without fMLF was added to the slides to create a gradient, and images of neutrophils were taken every 30 s for 30 min at 37 °C. The paths of individual neutrophils were tracked, and analysis of the tracks was performed using the “TrackMaxima” plug-in in ImageJ. The methods are described in detail in the Supplementary Information.

### 2.7. T Cell and B Cell Immune Phenotyping

The cellular expression of molecular biomarkers for immune regulation and/or inflammation in the context of viral infection and massive stimulation of the immune system was measured by quantitative fluorescence-activated cell sorting (FACS) technology using a BD LSR Fortessa System (BD Biosciences, Franklin Lakes, NJ, USA). Peripheral blood mononuclear cells (PBMCs) were isolated from heparinized whole blood samples (BD lithium heparin vacutainer tubes, BD Biosciences, Franklin Lakes, NJ, USA) within 3 h after blood donation. Whole blood was diluted 1:1 with PBS (ThermoFisher Scientific, Waltham, MA, USA) and layered into a tube prefilled with Lymphoprep density gradient media (Stemcell Technologies, Cologne, Germany). Density gradient centrifugation was performed (20 min, 800× *g*, RT) and the PBMC layer was collected and washed with PBS. Cell viability and cell number were measured using an automated dual-fluorescence cell counter (LUNA-FL, Logos Biosystems, Villeneuve d’Ascq, France) prior to multi-parameter staining of approximately 1 × 10^6^ cells per FACS panel. In total, 3 panels, targeting T and B cell subtypes, were stained per PBMC sample. All antibodies were purchased from Becton Dickinson (Franklin Lakes, NJ, USA) or Thermo Fisher (Waltham, MA, USA) (Table S1). Surface panel staining was performed using BD Lyse/Fix buffer according to the manufacturer’s instructions (BD Biosciences, USA). Intracellular staining was performed using Fix Perm buffer kits (BD Biosciences, USA) combined with surface and viability staining prior to permeabilization. UltraComp eBeads (ThermoFisher, Germany) were used for compensation, and specific fluorescence-minus-one (FMO) controls were applied to appropriate gating of T cell and B cell subtypes. A multiplex gating strategy (for detailed information about the exact definition of the gated population, see Table S1) was applied to quantify T cell subtypes, such as regulatory T cells (Tregs), naïve, memory, effector, and exhausted T cell subtypes (TEMRA cells) within panel 1 and 2. B cell subtypes, including naïve, memory, resting, exhausted, and transitional B cells, were gated by a combination of antibody signals within staining panel 3.

### 2.8. Statistical Analysis

Differences in biomarkers between patients after severe and mild COVID-19 disease were assessed with Student’s T-tests or Mann–Whitney U-tests, as appropriate. The effects of the probiotic intervention were described by calculating the change between the baseline values and the values after 3 and 6 months of intervention and testing the differences between groups with either Student’s T-tests or Mann–Whitney U-tests, as appropriate. Data were analyzed using R version 4.2.2 (31 October 2022 ucrt) and the additional packages rcompanion, DescTools, tidyverse, readxl, and writexl.

For statistical analysis of the microbiome composition, count table, taxonomy, and sample data were handed off to phyloseq, an R package for metagenomic analysis. Alpha diversity was estimated from an even count table, rarefied to the minimum read count per sample, using the metrics observed OTUs, Shannon index, inverse Simpson (implemented in the phyloseq::estimate_richness() function), and evenness (calculated as Shannon-index/log(observed OTUs). For beta-diversity analysis, Bray–Curtis dissimilarity was used as the foundation for the distance matrix, calculated with the phyloseq::ordinate() function and permutational multivariate analysis of variance using distance matrices (PERMANOVA) to determine whether there was significant clustering among the test groups, using the vegan::adonis2() function. Principal coordinate analysis (PCoA) was used for low-dimensional visualization (phyloseq::plot_ordination()). Redundancy analysis (RDA) was performed on a Hellinger-transformed abundance table to determine influencing factors on the microbiome composition using the vegan::rda() function. To test for differences in the overall change in the microbiome, the pldist R package was used to determine the intra-individual change between timepoints. Bray–Curtis dissimilarities were calculated, and PCoA/PERMANOVA as well as distance-based redundancy analysis (vegan::dbrda()) were performed to identify significant influences on the microbiome changes during the study. To describe changes in the microbiome in more detail, multivariate association analysis (MaAsLin2) was applied on microbiome changes over time, as obtained with pldist-tools. In addition to the above-mentioned R packages, the following packages were used: ggpubr, lme4, jtools, ggplotify, ggConvexHull, ggrepel, and ggh4x.

Predicted metagenomics were calculated via the application “TAX4FUN2” implemented on the web-based analysis platform “microbiome analyst” (https://www.microbiomeanalyst.ca/, Version 2.0, last visited on 9 March 2023), based on a count table with SILVA-based taxonomic annotations, randomized to the minimum read count. Gene abundances were summarized to pathways for further analysis. Statistical analysis was analogous to the above-described analysis of the microbiome data set.

The statistical analysis of the metabolomics data was performed using the web-based analysis platform “MetaboAnalyst” (https://www.metaboanalyst.ca/, Version 5.0, last visited 3 July 2023). Orthogonal or sparse partial least squares discriminant analysis was used to estimate the metabolome’s overall similarity between groups and identify important discriminant features. Changes in the metabolome due to the intervention were identified with multiple linear regression, similar to the multivariate association analysis of the microbiome data set. In addition, a targeted analysis of short-chain fatty acids in the stool metabolome was conducted with ANOVA and Sidak’s multiple comparison test.

To quantify the co-relationship between matched data sets, multiple co-inertia analysis was applied using the cia() function from the R package “made4”. Unsupervised multi-omics analysis was performed with multi-omics factor analysis (MOFA) implemented in the R package “MOFA2” across microbiomics, predicted metagenomics, stool, serum metabolomics, and B- and T cell populations. Before entering the model, the microbiomics data set was subjected to Hellinger transformation, while all other data sets were initially log(x + 1)-transformed and then mean centered and normalized to the standard deviation. Based on the data structure and the small sample size, the number of latent factors extracted from the MOFA-model was limited to five.

## 3. Results

### 3.1. Clinicopathological Characteristics

**Figure 1:**
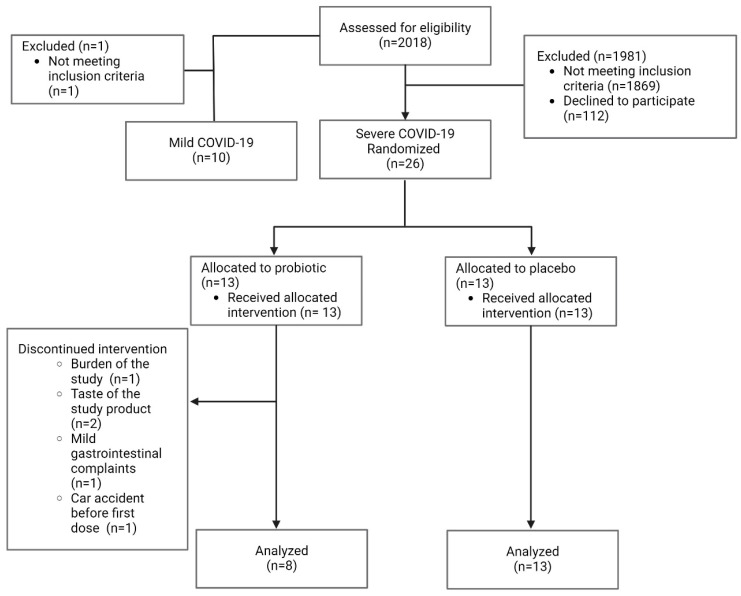
Patient flow chart.

We included 36 patients in this study, of whom 10 patients were included after mild COVID-19 infection and 26 after severe COVID-19 infection. The 26 patients after severe COVID-19 infection were included in a probiotic intervention trial, with 13 allocated to the probiotic group and 13 allocated to the placebo group. Eight patients in the probiotic and thirteen patients in the placebo group finished the study according to the protocol and were included in the analysis; five patients in the probiotic group dropped out during the study (*p* = 0.039, Figure 1). Four of the five dropouts were directly or indirectly related to the study—the burden of the study, the taste of the study product, and mild gastrointestinal complaints were observed as reasons for dropout. A total of 91% of patients after severe COVID-19 infection were hospitalized, 76% needed oxygen, and 43% received intensive care treatment. Patients after severe COVID-19 infection were included on average 318 days after the positive test, whereas patients after mild COVID-19 infection were included significantly earlier, after 221 days on average. Patients’ characteristics are listed in Table 1 (patients after severe COVID-19 infection compared to patients after mild COVID-19 infection) and Table 2 (baseline comparison between patients receiving the probiotic and the placebo treatment). Patients after severe COVID-19 infection were older, had a higher BMI/were more often obese, and were physically more active but had similar smoking status and alcohol drinking habits compared to controls after mild COVID-19 infection. Patients after severe COVID-19 infection had significantly more comorbidities, according to the Charlson comorbidity index [33]. In terms of routine biochemistry, patients after severe COVID-19 infection had slightly lower baseline serum albumin and total protein, serum calcium, and estimated glomerular filtration rate but higher fasting glucose levels compared to patients after mild COVID-19 infection. Patients after severe COVID-infection had significantly lower baseline scores for general health, and physical functioning in the SF-36 questionnaire, and lower physical function and symptoms scores in the GIQLI questionnaire and were bothered slightly more by medical treatment, compared to patients after mild COVID-19 infection. The ARTIQ questionnaire revealed that patients after severe COVID-infection experienced more lower-airway symptoms compared to patients after mild COVID-19 infection. Fewer patients recovered completely from severe COVID-19 infection, and their recovery and mMRC score were worse (*p* = 0.003, *p* = 0.004, and *p* = 0.006, respectively) than those in the mild COVID-19 group (Table S2).

No differences in baseline characteristics and routine laboratory parameters between those patients randomized to the probiotic intervention and those randomized to the placebo intervention were observed (Table 2).

### 3.2. Marker of the Gut–Lung Axis in Patients After Severe COVID-19 Infection Compared to Patients After Mild COVID-19 Infection

#### 3.2.1. Microbiome Differences Between Patients After Severe COVID-19 Infection and After Mild COVID-19 Infection

For microbiome analysis, 37,781.85 (±11,247.93) raw reads and 20,661.6 (±6973.00) high-quality reads per sample were available on average. Read counts for each group, and time points are given in Table S3. The minimum read count per sample was 9512, which was also used as the rarefaction depth for alpha diversity analysis.

**Figure 2:**
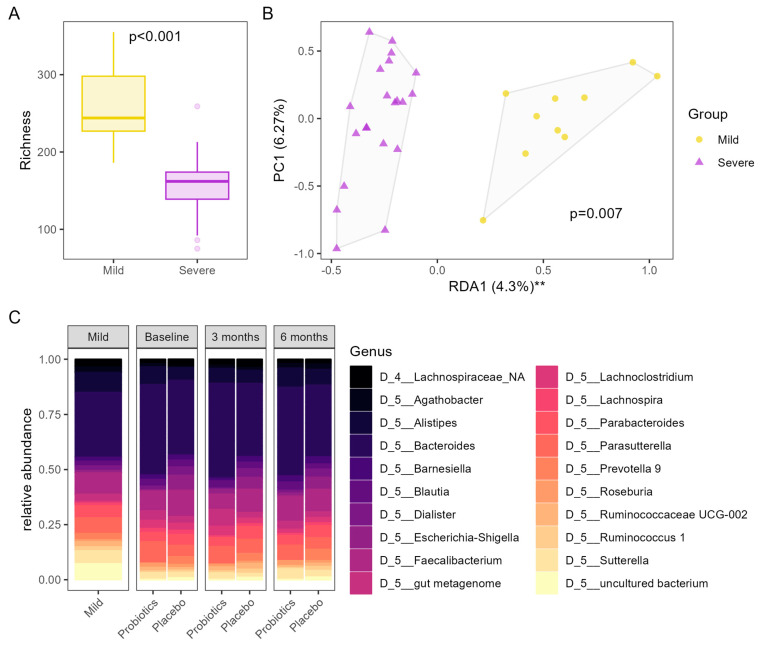
Microbiome composition showed significant differences between patients after severe COVID-19 infection compared to patients after mild COVID-19 disease. (**A**) A reduction in microbial richness was observed in patients after severe disease (purple) compared to patients after mild disease (yellow); *p*-values derived from the Mann–Whitney test. (**B**) Significant differences in the composition of the microbiome in patients after severe disease (purple) compared to patients after mild disease (yellow); *p*-value derived from redundancy analysis. ** *p* < 0.01 (**C**) Overview of the 20 most abundant genera in patients after mild disease (“Mild”) and patients after severe disease shown separately for patients allocated to “Probiotic” or “Placebo” interventions at each timepoint of the intervention.

At baseline, patients after severe COVID-19 infection showed a significantly lower microbial richness in their microbiomes compared to patients after mild COVID-19 infection (*p* = 0.001), a reduced Shannon index (*p* = 0.005), and a trend towards a reduced inverse Simpson index (*p* = 0.07). Patients after severe COVID-19 and patients after mild COVID-19 infection were clustered distinctly in a principal coordinate plot based on Bray–Curtis dissimilarities (PERMANOVA: *p* = 0.002), and redundancy analysis identified severe COVID-19 infection as a significant influence on the gut microbiome (RDA: *p* = 0.007). The overall composition of the microbiomes in groups and time points are shown in Figure 2 and Figure S1–S4.

The difference in microbiome composition is characterized by a reorganization of the families Ruminococcaceae and Lachnospiraceae. While some genera, such as *Ruminococcus 1*, *Eubacterium xylanophilum* group, *Ruminococcaceae NK4A214* group, *Eubacterium runimatium*, *Ruminococcus gauvreauii* group, and *Coprococcus*, were decreased in patients after severe COVID-19 infection, the genera *Flavonifractor* and *Ruminococcus gnavus* group were increased (for more details, see Figure S5). Co-inertia analysis following non-symmetric correspondence analysis showed a low global similarity between differentially abundant genera and symptoms assessed by ARTIQ (RV coefficient = 0.21, see also Figure S6). On the family level, neither Lachnospiraceae nor Ruminococcaceae showed significant differences between patients after severe COVID-19 infection and controls after mild COVID-19 infection. Differentially abundant families are shown in Figure S7. The functional potential of the microbiome estimated by Tax4Fun2 showed no differences in the alpha or beta diversity of predicted KEGG pathways between patients after mild or severe COVID-19 infection (see also Figure S8).

#### 3.2.2. Gut Permeability and Bacterial Translocation Differences Between Patients After Severe COVID-19 Infection and After Mild COVID-19 Infection

Patients after severe COVID-19 infection had higher DAO levels (*p* = 0.035), indicating increased intestinal permeability, but unaltered levels of zonulin in serum, LBP, and sCD14, indicating no apparent differences in bacterial translocation (Table S2).

#### 3.2.3. Metabolomics Differences Between Patients After Severe COVID-19 Infection and After Mild COVID-19 Infection

**Figure 3:**
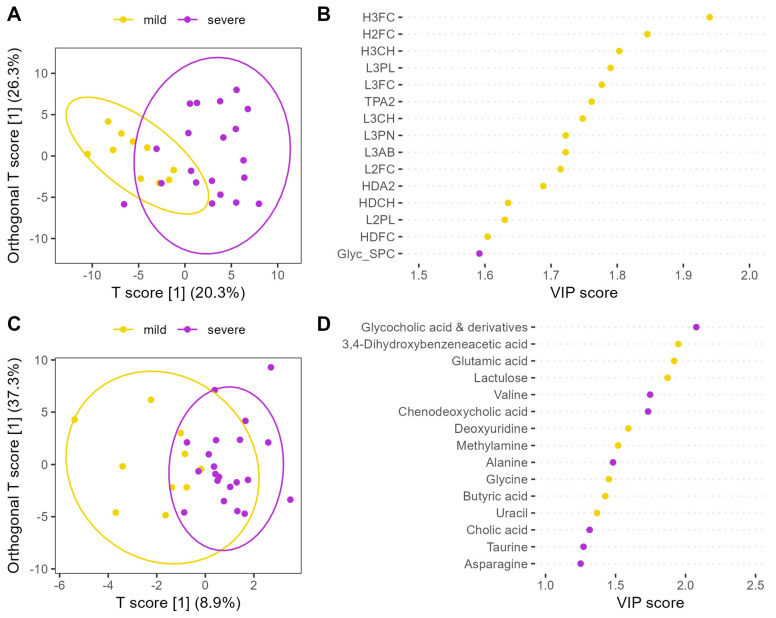
Differences in serum and stool metabolomics between patients after mild and severe COVID-19 disease. (**A**) oPLS-DA score plot indicating differences between the patients after mild and severe disease in the serum metabolome. (**B**) Features of high importance to the oPLS-DA model in (**A**) and their variable importance in projection (VIP) score; colors indicate in which group the feature was more abundant (purple: more abundant after severe disease; yellow: more abundant after mild disease). (**C**) oPLS-DA score plot indicating differences between the patient group in the stool metabolome; (**D**) Features of high importance to the oPLS-DA model in (**C**) and their variable importance in projection (VIP) score; colors indicate in which group the feature was more abundant (purple: more abundant after severe disease; yellow: more abundant after mild disease). H3FC—HDL-3 free cholesterol, H2FC—HDL-2 free cholesterol, H3CH—HDL-3 cholesterol, L3PL—LDL-3 phospholipids, L3FC—LDL-3 free cholesterol, TPA2—total Apo-A2, L3CH—LDL-3 cholesterol, L3PN—LDL-3 particle number, L3AB—LDL-3 Apo-B100, L2FC—LDL-2 free cholesterol, HDA2—HDL Apo-A2, HDCH—HDL cholesterol, L2PL—LDL-2 phospholipids, HDFC—HDL free cholesterol, Glyc_SPC—glycoprotein to supramolecular phospholipid composite ratio.

Serum metabolomics analysis showed significant differences between patients after severe and mild COVID-19 disease, as shown in Figure 3A,B. These differences were mainly characterized by a profound dysregulation of lipoproteins, where VLDL particles and their components, such as (free) cholesterol, phospholipids, and triglycerides, were significantly higher, and HDL particles and their components were significantly lower in patients after severe disease compared to patients after mild disease. Details are given in Table S4. In addition, a decrease in glutamine and an increase in phenylalanine, citric acid, acetoacetic acid, and the glycoprotein to supramolecular phospholipid composite (SPC) ratio—a novel biomarker for infection/inflammation—were observed in patients after severe disease.

Stool metabolomics also showed differences between patient groups. Patients after severe COVID-19 disease showed increased levels of glycocholic acid and its derivates, and decreased levels of 3,4-dihydroxybenzeneacetic acid (DOPAC), glutamic acid, and lactulose as indicated by oPLS-DA (Figure 3C,D and Table S5).

#### 3.2.4. Innate and Adaptive Immunity Differences Between Patients After Severe COVID-19 Infection and After Mild COVID-19 Infection

The macrophage activation parameter sMR was higher in patients after severe COVID-19 infection (*p* = 0.008), but sCD163 did not differ between the groups (Table S2). Neutrophil function analysis showed that the directedness of chemotaxis, as well as the ROS production in response to *E. coli*, was significantly lower (*p* = 0.035 and *p* = 0.009, respectively, Table S2), in patients after severe COVID-19 infection compared to patients after mild COVID-19 infection. Other chemotaxis parameters (chemotactic efficiency index, random migration, and chemotaxis towards fMLF) and ROS production in response to PBS, fMLF, and PMA did not differ between patients after mild or severe COVID-19 infection.

**Figure 4:**
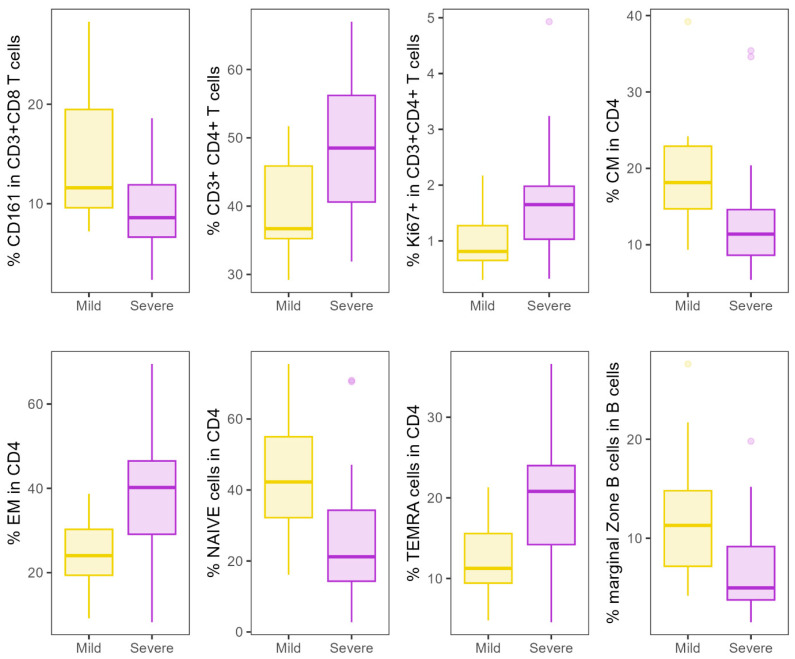
Differences in B- and T cell populations between patients after mild and severe COVID-19 infection before intervention; depicted cell populations showed a significant difference between patients after severe and patients after mild COVID-19 disease in Mann–Whitney U tests (all *p* < 0.05). CM—central memory T helper cells; EM—effector memory T cells; TEMRA—terminally differentiated effector memory cells.

At baseline, differences in T- and B cell subpopulations were found when comparing patients after severe COVID-19 infection with patients after mild COVID-19 infection (Figure 4). We observed a lower number of CD3+CD4+ T helper cells (*p* = 0.013) in patients after mild infection as compared to patients with severe infection, whereas no significant differences were found in CD3+CD8+ cytotoxic T cells between the groups. Within CD3+CD4+ T helper cells the following subtypes were significantly increased in patients after severe infection: effector memory T cells (EM; *p* = 0.009), terminally differentiated effector memory cells (TEMRA; *p* = 0.035), and proliferative, Ki67-expressing memory cells (*p* = 0.028). On the other hand, lower central memory T helper cells (CM; *p* = 0.024), naïve T helper cells (*p* = 0.031), CD161+ cytotoxic T cells (*p* = 0.04), and marginal zone B cells (*p* = 0.021) were observed in patients after severe disease. Significant differences did not withstand multiplicity correction. Details are given in Table S6.

#### 3.2.5. Multi-Omics Analysis

**Figure 5:**
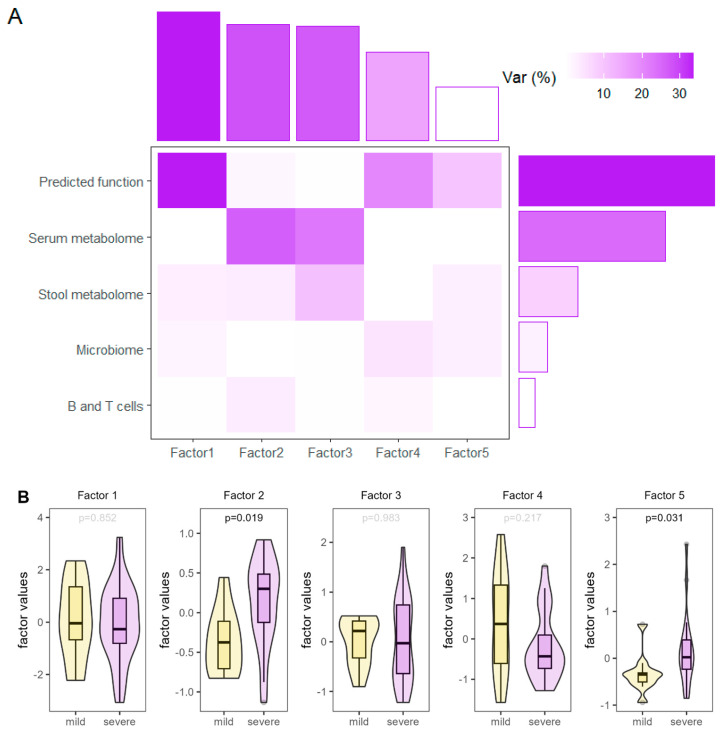
Summary of multi-omics factor analysis. (**A**) Explained variance by factor and view (i.e., type of data); (**B**) Values of identified latent factors which explain the most variance in the data set shown for patients after mild or severe COVID-19 disease; *p*-values derived from Mann–Whitney tests.

To identify latent factors across the microbiome, predicted metagenomics, serum and stool metabolomics data sets, and the B- and T-cell populations, multi-omics factor analysis was applied. Five latent factors were identified with varying degrees of explained variance, as seen in Figure 5. Factor 2 and 5 showed significantly higher values in patients after severe COVID-19 disease compared to patients after mild disease. As shown in Figures S9 and S10, these differences were mostly driven by VLDL particle number and predicted glucose and mannose metabolism capacity, which were significantly higher in patients after severe COVID-19 disease, and reflected the higher body mass index and possibly accompanying dyslipidemia of this patient group.

### 3.3. Modulation of the Gut–Lung Axis by a Multispecies Probiotic in Patients After Severe COVID-19 Infection

#### 3.3.1. Clinical Data

The patients in the probiotic group felt significantly less tired in the ARTIQ questionnaire after 3 months (*p* = 0.014, Tables S7 and S8), but after 6 months this only sustained as a a trend (*p* = 0.07). Also, the psychological burden significantly improved after 3 months of probiotic intervention (*p* = 0.022). No other significant changes between probiotic- and placebo-treated patients were observed over time in the quality of life questionnaires and in the routine laboratory parameters (Tables S9–S12).

#### 3.3.2. Microbiome Modulation by a Multispecies Probiotic in Patients After Severe COVID-19 Infection

**Figure 6:**
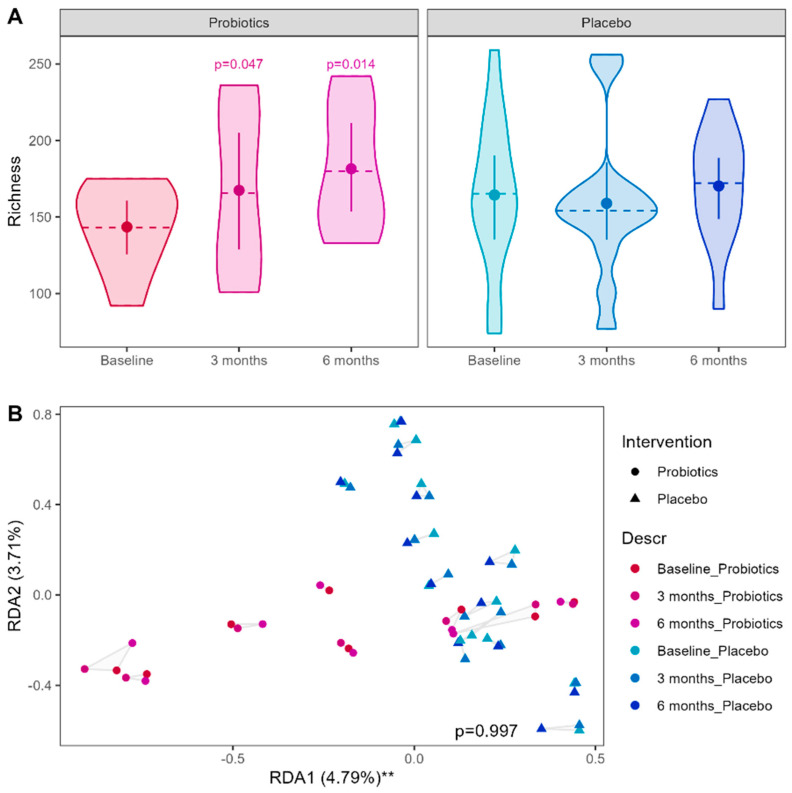
Effects of the probiotic intervention on the gut microbiome of patients after severe COVID-19 infection. (**A**) Alpha diversity over time in both treatment groups estimated by microbial richness, dashed lines represent the median, point (error bar) represent mean (CI95%); *p*-values derived from a mixed-effect model accounting for the allocation, timepoint and repeated measurements. (**B**) Redundancy analysis (RDA) of the microbiome composition of both groups over time. ** *p* < 0.01.

Patients after severe COVID-19 infection allocated to the probiotic group showed a significant improvement in microbial richness after 3 months (*p* = 0.047) and 6 months (*p* = 0.014) of intervention, which was not observed in the placebo group (Figure 6). The other alpha diversity metrices did not show significant changes, but there was a statistical trend towards decreased evenness of the microbiome in the probiotic group after 3 months of intervention (*p* = 0.06). Patients in both groups showed a comparable microbiome composition in general, although patients in the placebo group showed a more pronounced inter-individual variation in their microbiome compositions (PERMANOVA: *p* = 0.001) compared to the probiotic group; however, the composition of the microbiomes did not change during the intervention (PERMANOVA: *p* > 0.999). Similarly, RDA identified group allocation to account for some of the variation in the microbiome compositions (variance = 0.029, F = 2.14, *p* = 0.001), as well as the individual variation (variance = 0.034, F = 2.52, *p* = 0.001), but did not detect a significant influence of the probiotic intervention on the microbiome over time (variance = 0.008, F = 0.28, *p* > 0.999). Furthermore, no significant associations between the intervention and the observed taxa on any taxonomic level could be observed. In line with the hypothesis-generating nature of the study, the variation in the differentially abundant families associated with severe COVID-19 infection over time within the two intervention groups was separately examined for statistical trends; however, no indication of a probiotic modulation of any specific taxa could be found. No influence of the intervention on predicted metagenomics could be observed (Figure S11).

#### 3.3.3. Gut Permeability and Bacterial Translocation Was Unchanged by a Multispecies Probiotic in Patients After Severe COVID-19 Infection

None of the biomarkers of gut permeability or bacterial translocation were altered by the intervention. Details are given in Tables S13 and S14.

#### 3.3.4. Metabolomics Changes by a Multispecies Probiotic in Patients After Severe COVID-19 Infection

After 3 months of intervention, patients in the probiotic group showed a temporary decrease in lipoprotein components (total Apo-A2, total cholesterol, HDL cholesterol, HDL Apo-A2, HDL-4 Apo-A1, HDL-4 Apo-A2, HDL-4 phospholipids, HDL-4 cholesterol, HDL-4 free cholesterol, HDL-3 free cholesterol, LDL particle number, LDL phospholipids, LDL Apo-B100, LDL cholesterol, LDL free cholesterol, LDL-1 particle number, LDL-1 phospholipids, LDL-1 Apo-B100, LDL-1 cholesterol, and LDL-1 free cholesterol). H4A2 and HDCH remained significant after multiplicity correction (see Figure S12). The decreased lipoprotein components returned to baseline values after 6 months of intervention, while values remained stable in the placebo group. After 6 months, a decrease in glycine in the probiotics group and increased histidine in the placebo group were observed.

Stool metabolomics showed an increase in 3,4-dihydroxybenzeneacetic acid (DOPAC), fumaric acid, tyrosine, proline, and deoxyuridine and a decrease in cholic acid and malonic acid in the probiotic group after 6 months of intervention. The changes did not withstand multiplicity control. There were no changes in the stool metabolome after 3 months of intervention (see Figure S13).

A focused analysis of short-chain fatty acids, i.e., butyric acid, acetic acid, and propionic acid, in stool showed a significant increase in butyric acid after 6 months of probiotic intervention. Other short-chain fatty acids were unaffected (see Figure S14).

#### 3.3.5. Innate and Adaptive Immunity Changes by a Multispecies Probiotic in Patients After SeVere COVID-19 Infection

Macrophage activation markers were not altered through the intervention (Tables S15 and S16). In terms of neutrophil function, we observed a significantly lower ROS production in response to fMLF, *E. coli*, and PMA in the probiotic group compared to the placebo group after 3 months of treatment (*p* = 0.033, *p* = 0.01, *p* = 0.034, respectively), for details see Table S17. No significant changes were observed between probiotic- and placebo-treated patients for neutrophil chemotaxis (Table S18).

**Figure 7:**
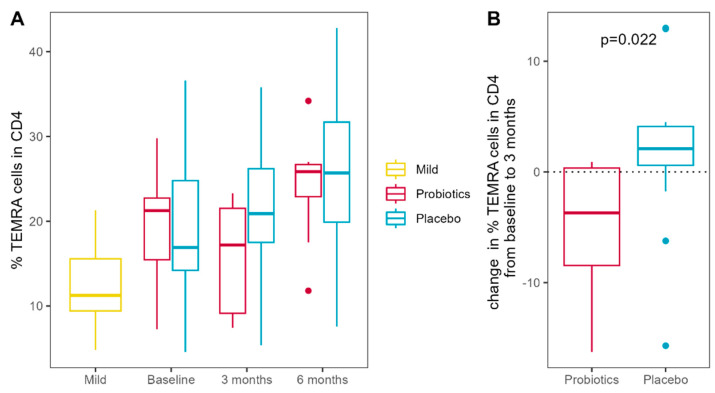
(**A**) Percentage of terminally differentiated effector memory cells (TEMRA) cells in the CD4-positive population throughout the study period; (**B**) Change in percentage of TEMRA cells within the first 3 months of intervention in the probiotic and placebo groups. Patients after mild COVID-19 infection serve as the control.

After 3 months of intervention, the percentage of CD4+ terminally differentiated effector memory cells decreased significantly in the probiotic group, while the placebo group showed a slight increase (TEMRA; *p* = 0.022). Terminally differentiated effector memory cells increased non-significantly in both groups over the next 3 months (Figure 7). Notably, there was a trend towards increased regulatory T cells (Tregs) in the probiotic group after 6 months of intervention compared to baseline. Overall, there were no differences in intervention response in any cell population after 6 months of intervention (details are given in Tables S19 and S20).

## 4. Discussion

We conducted a pilot study in patients after mild and severe COVID-19 infection to identify potentially modifiable biomarkers of the gut–lung axis. We identified distinct differences between patients after mild and after severe COVID-19 infection in gut microbiome composition, gut permeability, and serum, as well as stool metabolome composition and immune parameters, whereas most routine laboratory parameters were unchanged. As expected, quality of life was impaired after severe COVID-19 infection. Patients after severe COVID-19 infection also received an intervention which aimed to modulate gut microbiome composition and/or function. The treatment was associated with the improvement of some clinical parameters, such as microbiome composition and microbial-derived metabolites, as well as lipoprotein composition and markers of innate and adaptive immunity.

Patients after severe COVID-19 infection in our cohort were included approximately 10 months after the infection, whereas those after mild infection were studied approximately 7 months after the infection. Despite being studied later after the infection, patients after severe COVID-19 infection showed significantly lower baseline scores for general health and physical functioning and still suffered from lower airway symptoms, which is in line with many other reports: a meta-analysis of 63 studies showed that that fatigue and dyspnea were found in 37% and 21% of patients, respectively, 9–12 months after the infection [34]—this is comparable with our cohort, where fatigue was found in 41% and dyspnea in 24% of the patients after severe COVID-19 infection. At the time we designed the study, there was no consensus available on standardized symptom definition for post-acute COVID-19 syndrome. We therefore aimed to analyze recovery and the respiratory function, symptoms, and conditions using the modified Medical Research Council Dyspnea Scale, as suggested by the Post COVID Condition (Long COVID) Core Outcome Set project [20] and found that, while 80% of patients after mild COVID-19 infection showed full recovery and no residual symptoms, only 24% of patients after severe COVID-19 infection were fully recovered at the time of the study. These findings underpin the need to better understand the pathophysiological basis of slow recovery after COVID-19, even when the threat of the pandemic has vanished after quick introduction of vaccination and a steep reduction in the number of severe cases [35]. Our study indicates that long-term consequences of severe COVID-19 infection that are associated with the gut–lung axis may persist. The importance of the gut–lung axis in general [36], and specifically in COVID-19, has recently gained attention [11,37,38]. Reduced alpha diversity has been described in the acute setting [39] as well as after 6 months [40]. Low microbial richness was related to worse symptoms at 6 months. The persistence of COVID-19-induced alterations in the gut microbiome during the acute phase of the disease and the influence of the persisting alterations on post-acute disease severity, highlights the bidirectional nature of the gut–lung axis. We confirm and extend the finding of a reduction in diversity to around 10 months after the acute infection. The observed reorganization of the families Ruminococcaceae and Lachnospiraceae is also in line with available evidence where Ruminococcaceae and especially the depletion of *Faecalibacterium prausnitzii* has been implicated as a potential marker for COVID-19-associated alterations of the microbiome [39,41,42,43,44]. Persisting microbiome alterations after a severe SARS-CoV-2 infection align with previous findings from our group, where we showed long-term microbiome alterations in patients after intensive care treatment [45]. Our cohort of patients after severe COVID-19 infection also showed a higher BMI and higher comorbidities, which is not unexpected, since obesity and comorbidities belong to the well-known risk factors that may have predisposed them to the severe disease course [46,47]. Obesity has been known for more than 15 years to be associated with alterations in the gut microbiome, such as reduced diversity and a reduction in strains that exhibit positive effects on the host, such as the production of short chain fatty acids [48]. Since we do not have data on the microbiome composition of our cohort before the patients were infected with COVID-19, we cannot exclude that diversity was already lower before the infection. It is also likely that the pandemic, with physical separation, extensive hygiene, travel barriers, and other measures that influence overall loss in microbial diversity and prevent re-inoculation, may additionally contribute to a decades-long decline in microbial diversity due to hygiene, antibiotics, and urban living in many parts of the world, therefore multiple factors may contribute to our finding of dysbiosis after severe COVID-19 infection [49].

Increased gut permeability and bacterial translocation were described during acute COVID-19 illness [50] and up to 6 months after the illness [51]. Here, we describe signs of long-term alteration in gut permeability after severe COVID-19 infection, since DAO was still elevated in patients after severe COVID-19 infection, whereas markers of bacterial translocation were not different in patients after mild and severe disease. Innate immune response was also mainly studied in the acute setting where exaggerated ROS production and NETosis were found in critically ill patients [52] and where dysregulated innate immune response is thought to contribute to hyperinflammatory syndrome [53]. We provide evidence of alterations in macrophage activation and neutrophil dysfunction, as well as alterations in B- and T-cell subsets in the post-acute setting. Higher sMR levels and less directed chemotaxis as well as lower ROS response to *E. coli* can be considered as mild defects in innate immune function that could predispose patients after severe COVID-19 infection to further infections. sMR is strongly expressed on interstitial and alveolar macrophages, and an association of elevated sMR levels and poor prognosis in inflammatory lung diseases has been established [54]. This is in accordance with a study showing macrophage activation through sCD163 in comparison to healthy donors [51]; however, sCD163 was not elevated in our study compared to in patients after mild disease. The same study also showed an upregulation of genes related to neutrophil activation [51]. T- and B- cell immune phenotyping showed differences between patients after mild and severe COVID-19 infection. The T- cell arm of the adaptive immune system is vital in the host defense against viral pathogens and in long-lasting immune memory that prevents reinfection, while a robust CD4+ T helper cell response is needed to activate B cells that transform into plasma cells and plasmablasts, which produce specific antibodies to fight viral infection. Although the differences did not withstand multiplicity correction, the shift in the populations indicates long-term changes in T/B cell subsets, depending on the disease severity as well as the potential to modulate T cell subsets through microbiome modulation. In acute COVID-19 infection, a striking loss of T cells, particularly of naïve CD4+ T cells, but an increase in effector and memory subsets, has been observed [55]. Interestingly, in chronic inflammatory conditions, such as patients undergoing dialysis, increased CD38+CD8+ effector memory and TEMRA T cells as well as CD161+CD8+ T cells have been observed that might protect patients from a severe disease course [56]. After clearing the infection, T cell subpopulations have been shown to recover in several studies, but abnormalities may persist after the resolution of infection [56]. These populations could lead to an increased susceptibility to infections and autoimmune diseases or explain clinical symptoms of long COVID-19. In a recent study in patients with long COVID symptoms, a miscoordination between their SARS-CoV-2-specific T and B cell responses was described, indicating that the proper crosstalk between the humoral and cellular arms of adaptive immunity is impaired [57]. In our study, we show baseline differences between patients with mild and severe disease. Severe COVID infections lead to a persistent decrease in CD4+ T helper cells even after several months, which supports the concept that a reduction in T helper cell counts in severe acute COVID patients persists at least for several months [58]. The remaining T helper cells are more often expressing naïve and central memory phenotypes when compared to mild cases. Additionally, proinflammatory CD161 expressing CD8+ cytotoxic cells, known as potent cytokine producers, and marginal zone B cells are increased, indicating an ongoing response to viral infections.

Also, untargeted metabolomics analysis could clearly discriminate between patients after mild and after severe COVID-19 infection at baseline. Serum samples in our cohort revealed more pronounced differences compared to stool samples. A multitude of studies aimed to find metabolomics signatures in the early phase of the disease to predict severity and outcome [59,60,61], suggesting a reprogramming of some metabolic pathways alongside the reprogramming of the immune system during the disease. There is evidence that metabolomics changes show “healing” after recovery from the disease, but persistent changes have also been observed, such as ketone bodies (3-hydroxybutyric acid, acetoacetic acid, and acetone), amino acids (methionine, tyrosine, valine), lactic acid, and acetic acid [62]. Lipid pathway dysregulation was found up to two years after diagnosis in comparison to acutely infected patients [63]. We could verify a profound dysregulation of lipoproteins, where VLDL particles and their components, such as (free) cholesterol, phospholipids, and triglycerides, were significantly higher, and high-density lipoproteins (HDL) and their components were significantly lower in patients after severe disease compared to patients after mild disease. A decrease in glutamine and an increase in phenylalanine, citric acid, acetoacetic acid, and the glycoprotein to supramolecular phospholipid composite (SPC) ratio—a novel biomarker for infection/inflammation—were observed in patients after severe disease. Acute COVID-19 infection causes a reduction in lipoprotein-bound serum phospholipids, which gives rise to SPC signals [64]. SPC was, therefore, proposed as a sensitive molecular marker for COVID-19 positivity [65]

Alterations in the gut–lung axis can weaken host immunity through multiple mechanisms, increasing susceptibility not only to COVID-19 itself but also to secondary bacterial and fungal infections [66]. A reduction in immunomodulatory gut bacteria may directly impair systemic and pulmonary immune responses. Additionally, lower levels of these beneficial bacteria reduce short-chain fatty acid (SCFA) production, which is crucial for maintaining gut barrier integrity [67]. This compromised barrier allows bacterial translocation into circulation, triggering immune activation and, ultimately, immune exhaustion, which elevates overall infection risk [68]. Furthermore, COVID-19-associated loss of microbial diversity reduces colonization resistance in the intestine, potentially opening ecological niches for pathogenic colonization in the gut [69]. This combination of factors highlights how the gut–lung axis’s disruption during COVID-19 can predispose patients to heightened infection risks and indicates the occurrence of secondary bacterial or fungal infections as a potential confounder in future studies. Our study is unique in the sense that we not only describe differences in a large set of biomarkers of the gut–lung axis in patients after COVID-19 infection, but we also aimed to and were successfully able to modify some biomarkers with an intervention targeted to modulate the microbiome. A potential therapeutic target in ameliorating post-acute COVID-19 symptoms could be the gut microbiome. Potential strategies to modulate the gut microbiome are fecal microbiota transplantation, probiotics, prebiotics, or diet [70]. Probiotics, which are live microorganisms that have beneficial health effects, have been studied to prevent gastrointestinal dysbiosis and to decrease the likelihood of secondary infections, as well as for their immunomodulating and antiviral properties. Despite considerable research efforts, high quality evidence is still limited in this field [71]. Probiotics were already in use early in the pandemic and were subsequently studied in COVID-19 disease and post-acute COVID-19 syndrome [14,15]. We chose the study product in our trial because of data on the anti-inflammatory, wound healing, and fibrosis protective effects of the strains in the product in addition to the expected antiviral effects of the individual strains. *Lactobacillus delbrueckii* ssp. *bulgaricus* has been shown to accelerate wound healing processes, to suppress NFkappaB signaling and inflammation, and to strengthen the intestinal barrier [72,73,74,75,76,77]. *Lactobacillus rhamnosus* also shows anti-inflammatory properties and strengthens the intestinal barrier [78,79,80,81], increases the resistance against viral infections [82], and thereby helps to avoid lung tissue damage during infection [83]. *Lactobacillus rhamnosus* further prevents liver fibrosis via a farnesoid X receptor-dependent pathway [84]. *Lactobacillus reuteri* is widely present in the intestines of healthy individuals and regulates the intestinal immune system by reducing inflammation through multiple mechanisms [85]. These pathways include a reduction in the production of proinflammatory cytokines in response to different stimuli in different animal and cell culture models via folate and histidine/histamine metabolism, as well as the alleviation of airway inflammation via the increase in butyrate production in the gut microbiome [85,86,87,88,89,90]. *Bifidobacterium animalis* ssp. *lactis* attenuates intestinal and hepatic inflammation in different animal models. Potential mechanisms that were identified include an increase in the short-chain fatty acid production of the microbiome and the promotion of regulatory T cells [91,92,93,94,95]

Our therapeutic intervention with a rationally selected multispecies probiotic was able to improve the clinical parameters and biomarkers of the gut–lung axis. Clinically we could show statistically significant improvements in quality of life, namely in the perception of tiredness and the psychological aspect of the ARTIQ. Since fatigue is one of the most important consequences after COVID-19 infection [96], that is frequently associated with work inability [97], improving fatigue is a very important goal that could be reached faster with our probiotic intervention compared to placebo treatment. Moreover, we could show an increase in microbial richness, as well as metabolomic changes and changes in innate and adaptive immune function markers. Increasing microbial richness of the microbiome is a desired aim in microbiome modulation, since we and others showed that severe COVID-19 disease is associated with a decrease in microbial richness [38]. This phenomenon has been observed in many acute and chronic diseases, as well as our modern lifestyle [98,99]. We could also modulate serum as well as stool metabolomic composition. In serum, HDL-related lipoprotein parameters, particularly HDL cholesterol, and HDL-4 Apo-A2, were reduced after 3 months of treatment and increased to baseline values again after 6 months, indicating changes in lipoprotein metabolism in the probiotic group. This is in agreement with several studies reporting no or minor changes in lipoprotein-related parameters upon treatment with probiotics [100,101]. The observed differential effects of the interventions on the serum amino acids glycine and histidine warrant further targeted analysis to understand their role in the gut–lung axis.

Stool metabolomics showed an increase in 3,4-dihydroxybenzeneacetic acid (DOPAC), fumaric acid, tyrosine, proline, and deoxyuridine, and a decrease in cholic acid and malonic acid in the probiotic group after 6 months of intervention, as well as an increase in butyrate when SCFAs were analyzed separately in line with the exploratory nature of our study. This finding is promising, since both DOPAC and butyrate are microbial metabolites associated with health, especially with quality of life and better mental health [102]. Microbial butyrate is the best-studied metabolite of the human gut microbiome that has immunomodulatory properties [103], can influence tumor cell growth [104,105], and can even affect distant organs or cells, for example, microglia differentiation in the brain [106]. Modulating the microbiome to produce more butyrate is also seen as a desired goal in COVID-19 [107]. In addition to these encouraging findings in clinical, microbiome, and metabolome parameters, we also observed improvements in innate and adaptive immune markers. Neutrophil functional analysis showed a decrease in ROS production towards different stimuli during the probiotic intervention, which can be interpreted as a positive finding, since COVID-19 infection is associated with excess ROS production [108] leading to tissue damage [109] during acute infection. To the best of our knowledge, there are no published data available to compare the present findings on the time course of ROS production in neutrophils after severe COVID-19 infection. Furthermore, we could show that probiotic treatment was associated with a decrease in CD4+ TEMRA cells after 3 months of therapy; however, this effect was not present at the end of therapy. CD4+ TEMRA cells, while clearly important in fighting viral infections, are also enriched in chronic inflammation and autoimmunity and are considered proinflammatory and cytotoxic but exhausted T cells [110]. A faster reduction in this T cell population after clearance of the infection is, therefore, a desired goal. Concurrently, a trend for an increase in the regulatory T helper cell population (Tregs) can be reported after 6 months of probiotic treatment in severe COVID-19 cases, which could benefit the recovery toward immune homeostasis.

Our study is limited by a small sample size; however, this was anticipated, and the study was intentionally designed as a pilot to identify biomarkers of the gut–lung axis and assess the potential to modulate these markers, which we achieved. Another limitation is the lack of data on viral shedding in stool, prolonged viral persistence in the gut, and viral genotype within our cohort. Given the cross-sectional design and the inclusion of patients 10 months post-acute disease, these data were beyond the study’s scope. Additionally, potential confounding factors, such as detailed medication regimens, baseline microbiome compositions, and pre-infection body weight and composition, could not be accounted for, as these were not collected for timepoints prior to or during the acute SARS-CoV-2 infection.

## 5. Conclusions

In conclusion, this study demonstrates distinct and persistent changes in clinical, microbiome, metabolomic, and immune parameters of the gut–lung axis in patients following severe COVID-19, some of which were partially modulated by a multi-species probiotic. This exploratory study aimed to identify promising directions for understanding and managing post-acute COVID-19 syndrome. Key findings include notable disruptions along the gut–lung axis; however, critical confounding factors—such as viral load, medication use, secondary or recurring infections, and comorbidities, particularly obesity and metabolic syndrome—must be accounted for in future studies. These insights provide a valuable foundation for further research on acute viral infections and the prevention or treatment of post-acute COVID-19 syndrome, especially as COVID-19 cases continue to recur and other respiratory viral infections might also result in post-acute infection syndromes with considerable clinical overlap to post-acute COVID-19.

## Figures and Tables

**Table 1:** Baseline characteristics of patients after severe and after mild COVID-19 infection.

	Severe (n = 21)	Mild (n = 10)	*p*-Value
Age (years)	56 (45; 62)	38 (34; 52)	0.031
Sex (female/male), n (%)	8 (38)/13 (62)	5 (50)/5 (50)	ns
Days between positive test and inclusion	318 (284; 332)	221 (203; 275)	0.013
Body mass index (BMI) (kg/m^2^)	28 (27; 31)	23 (21; 24)	<0.001
Obesity (BMI > 30 kg/m^2^) (yes/no), n (%)	7 (33)/14 (66)	0 (0)/10 (100)	0.038
Diabetes (yes/no), n (%)	3 (14.3)/18 (85.7)	0 (0)/10 (100)	ns
Arterial hypertension (yes/no), n (%)	8 (38)/13 (62)	0 (0)/10 (100)	0.023
Charlson comorbidity index (0/1–2/>2), n (%)	11 (52)/8 (38)/2(10)	10 (100)/0/0	0.02
Smoking status (smoker/ex-smoker/non-smoker)	2 (9)/9 (43)/10 (48)	2 (20)/1 (10)/7 (70)	ns
Smoking (packs/year)	17 (14; 35)	8 (2; 13)	ns
Alcohol consumption (g/week)	36 (18; 90)	11 (0; 180)	ns
Sports (hours/week)	5 (3; 7)	2 (2; 5)	0.035
Ongoing post-acute COVID-19 symptoms (yes/no), n (%)	13 (62)/8 (38)	NA	NA
Hospitalization during COVID-19 infection (yes/no), n (%)	19 (91)/2 (9)	NA	NA
Oxygen during COVID-19 infection (yes/no), n (%)	16 (76)/5 (24)	NA	NA
Intensive care unit during COVID-19 infection (yes/no), n (%)	9 (43)/12 (57)	NA	NA
Non-invasive ventilation during COVID-19 infection (yes/no), n (%)	10 (48)/11 (52)	NA	NA
Invasive ventilation during COVID-19 infection (yes/no), n (%)	4 (19)/17 (81)	NA	NA
Tracheotomy during COVID-19 infection (yes/no), n (%)	1 (5)/20 (95)	NA	NA
Extracorporeal membrane oxygenation support during COVID-19 infection (yes/no), n (%)	2 (9)/19 (91)	NA	NA
Albumin (g/dL)	4.6 (4.5; 4.7)	4.7 (4.7; 5.0)	0.007
Total protein (g/dL)	7.3 (7.2; 7.6)	7.6 (7.6; 8)	0.003
Calcium (mmol/L)	2.37 (2.35; 2.39)	2.44 (2.39; 2.54)	0.02
Glomerular filtration rate (mL/min)	87.2 (81.3; 99.2)	103.0 (90.8; 114.7)	0.022
Fasting glucose (mg/dL)	101 (94; 109)	86 (78; 91)	0.007

Data are shown as median and 95% confidence interval unless stated otherwise; NA, not applicable; ns, not significant.

**Table 2:** Baseline characteristics of patients after severe COVID-19 infection randomized to probiotic and placebo intervention.

	Probiotic (n = 8)	Placebo (n = 13)	*p*-Value
Age (years)	54 (45; 65)	60 (43; 63)	ns
Sex (female/male), n (%)	3 (38)/5 (62)	5 (39)/8 (61)	ns
Days between positive test and inclusion	320 (234; 346)	313 (254; 323)	ns
Body mass index (kg/m^2^)	28 (26; 36)	30 (27; 31)	ns
Obesity (BMI > 30 kg/m^2^) (yes/no), n (%)	3 (37.5)/5 (62.5)	4 (30.8)/9 (69.2)	ns
Diabetes (yes/no), n (%)	1 (12.5)/7 (87.5)	2 (15.4)/11 (84.6)	ns
Arterial hypertension (yes/no), n (%)	4 (50)/4 (50)	4 (30.8)/9 (69.2)	ns
Smoking status (smoker/ex-smoker/non-smoker)	2 (25)/3 (37.5)/3 (37.5)	0 (0)/6 (46)/7 (54)	ns
Smoking (packs/year)	25 (15; 35)	16 (8; 36)	ns
Alcohol consumption (g/week)	36 (8; 144)	18 (0; 72)	ns
Sports (hours/week)	6 (4; 10)	4 (2; 7)	ns
Charlson comorbidity index (0/1–2/>2), n (%)	6 (75)/1 (12.5)/1 (12.5)	5 (38)/7 (54)/1(8)	ns
Ongoing post-acute COVID-19 symptoms (yes/no), n (%)	6 (75)/2 (25)	7 (54)/6 (46)	ns
Hospitalization during COVID-19 infection (yes/no), n (%)	7 (88)/1 (12)	12 (92)/1 (8)	ns
Oxygen during COVID-19 infection (yes/no), n (%)	7 (75)/2 (25)	10 (77)/3 (23)	ns
Intensive care unit during COVID-19 infection (yes/no), n (%)	2 (25)/6 (75)	7 (54)/6 (46)	ns
Non-invasive ventilation during COVID-19 infection (yes/no), n (%)	3 (38)/5 (62)	7 (54)/6 (46)	ns
Invasive ventilation during COVID-19 infection (yes/no), n (%)	1 (13)/7 (87)	3 (23)/10 (77)	ns
Tracheotomy during COVID-19 infection (yes/no), n (%)	1 (13)/7 (87)	0 (0)/13 (100)	ns
Extracorporeal membrane oxygenation support during COVID-19 infection (yes/no), n (%)	0 (0)/8 (100)	2 (15)/11 (85)	ns
Albumin (g/dL)	4.6 (4.5; 4.8)	4.5 (4.4; 4.7)	ns
Total protein (g/dL)	7.5 (7.2; 7.9)	7.2 (6.8; 7.3)	ns
Calcium (mmol/L)	2.38 (2.35; 2.39)	2.37 (2.32; 2.41)	ns
Glomerular filtration rate (mL/min)	84.27 (74.49; 91.16)	90.99 (84.81; 105.69)	ns
Fasting glucose (mg/dL)	101.5 (93; 124)	100.5 (91; 109)	ns

Data are shown as median and 95% confidence interval unless stated otherwise; ns, not significant.

## Data Availability

The study was registered at clinicaltrials.gov (NCT04813718). Sequencing data are available at the National Center for Biotechnology Information Sequence Read Archive (NCBI SRA, https://www.ncbi.nlm.nih.gov/sra/, accessed on 6 October 2024) under the project accession number PRJNA999592. The nuclear magnetic resonance raw data has been deposited at MetaboLights under the accession number (the ID will be made available upon acceptance of the manuscript) (https://www.ebi.ac.uk/metabolights/, accessed on 6 October 2024). All other individual patient data are available upon reasonable request from the corresponding author.

## Supplementary Materials

The following supporting information can be downloaded at: https://www.mdpi.com/article/10.3390/nu16223840/s1. All references mentioned in the Supplementary Materials are also cited in the main text for consistency. Table S1. Antibodies, clones, fluorochromes, manufacturer and reference number of the antibodies used for B- and T-cell analysis. Table S2. Baseline characteristics of patients after severe COVID-19 infection (n = 21) and after mild COVID-19 infection (n = 10). Table S3. Read counts per sample for each group and time point (data is given as mean and standard deviation). Table S4. Serum metabolomics including inflammatory proteins, small molecular metabolites and lipoproteins for patients after severe and mild COVID-19 disease. Values are given as median (95% confidence interval). Table S5. Stool metabolites in patients after mild and severe COVID-19 disease in arbitrary units. Data are given as median (95% confidence interval). Table S6. T- and B-cell populations in patients after mild and severe COVID-19 infection. Values are given as median and 95% confidence interval. Table S7. Baseline and 3 months of treatment values, and changes in ARTIQ, GIQLI and SF-36 questionnaire, recovery- and mMRC score parameters; data are given as median (95% confidence interval). Table S8. Baseline and end of treatment values, and changes in ARTIQ, GIQLI and SF-36 questionnaire, recovery- and mMRC score parameters. Table S9. Baseline and after 3 months of treatment values, and changes in standard laboratory parameters; data are given as median (95% confidence interval). Table S10. Baseline and end of treatment values, and changes in standard laboratory parameters; data are given as median (95% confidence interval). Table S11. Baseline and after 3 months of treatment values, and changes in lung function parameters; data are given as median (95% confidence interval). Table S12. Baseline and end of treatment values, and changes in lung function parameters; data are given as median (95% confidence interval). Table S13. Baseline and 3 months of treatment values, and changes in gut permeability and bacterial translocation parameters; data are given as median (95% confidence interval). Table S14. Baseline and end of treatment values, and changes in gut permeability and bacterial translocation parameters; data are given as median (95% confidence interval). Table S15. Baseline and 3 months of treatment values, and changes in macrophage activation markers; data are given as median (95% confidence interval). Table S16. Baseline and end of treatment values, and changes in macrophage activation markers; data are given as median (95% confidence interval). Table S17. Baseline and 3 months of treatment values, and changes in neutrophil function parameters; data are given as median (95% confidence interval). Table S18. Baseline and end of treatment values, and changes in neutrophil chemotaxis parameters; data are given as median (95% confidence interval). Table S19. B- and T-cell populations at baseline and after 3 months of intervention for patients in the probiotic group and the placebo group. Data are given as median (95% confidence interval). Table S20. B- and T-cell populations at baseline and after 6 months of intervention for patients in the probiotic group and the placebo group. Data are given as median (95% confidence interval). Figure S1. Taxa plot on Phylum level for all groups and time points. Figure S2. Taxa plot on Class level for all groups and time points. Figure S3. Taxa plot on Order level for all groups and time points. Figure S4. Taxa plot of the 20 most abundant families for all groups and time points. Figure S5. Differentially abundant genera at baseline assessed by MaAsLin2 comparing patients after severe COVID-19 disease to patients after mild COVID-19 disease (“Mild”). Patients after severe disease are shown according to their allocation in the intervention trial to show comparability of the microbiome before the intervention started (“Probiotics”/“Placebo”). All genera in this panel showed a q-value smaller than 0.05 after Benjamini Hochberg correction when comparing patients after mild to patients after severe disease. Figure S6. Co-inertia plot for differentially abundant genera and symptoms assessed with the ARTIQ questionnaire. Figure S7. Differentially abundant families identified by MaAsLin2 in patients after severe COVID-19 infections (placebo, verum (probiotics)) compared to controls after mild COVID-19 infection. Figure S8. Pathway diversity of predicted metagenomics based on Tax4Fun analysis of the 16S rRNA gene sequencing of patients after severe COVID-19 infections (placebo, verum (probiotics)) and controls after mild COVID-19 infection. Figure S9. Multi-omics factor analysis; details for factor 2. A. Heatmap for the 10 serum metabolites contributing the highest weights to the factor with hierarchical clustering and group allocation for the examined samples. Figure S10. Multi-omics factor analysis; details for factor 5. Figure S11. Pathway diversity of predicted metagenomics based on Tax4Fun analysis of the *16S rRNA* gene sequencing in patients treated with Probiotics (I.e. Verum) or Placebo. Figure S12. Serum lipoprotein parameters modulated by probiotic intervention. Figure S13. Stool metabolites modulated by probiotic interventions. Figure S14. Short chain fatty acids in stool throughout the study period for patient in the probiotic and the placebo group.
