# Supplementary material for: Alteration of the Gut–Lung Axis After Severe COVID-19 Infection and Modulation Through Probiotics: A Randomized, Controlled Pilot Study
Source: Nutrients. 2024 Nov 8;16(22):3840. doi: 10.3390/nu16223840 (PMC11597208; doi:10.3390/nu16223840)
Supplement: Supplementary file 1 [file nutrients-16-03840-s001.zip › nutrients-3272246-supplementary.pdf]

Supplementary information

# Alteration of the gut-lung axis after severe COVID-19 infection and modulation through probiotics: A randomized, controlled pilot study

Angela Horvath [1,2], Hansjörg Habisch [3,4], Barbara Prietl [1,5], Verena Pfeifer [1,5], Irina Balazs [1,2], Gabor Kovacs [6], Vasile Foris [6], Nikolaus John [6], Daniela Kleinschek [7], Nicole Feldbacher [1,2], Henning Grønbæk [8], Holger Jon Møller [9,10], Kristina Žukauskaitė [2,11], Tobias Madl [3,4], Vanessa Stadlbauer [1,2]

[1]Center for Biomarker Research in Medicine (CBmed), Graz, Austria;

[2]Division for Gastroenterology and Hepatology, Department of Internal Medicine, Medical University of Graz, Graz, Austria;

[3]Gottfried Schatz Research Center for Cell Signaling, Metabolism and Aging, Molecular Biology and Biochemistry, Medical University of Graz, Graz, Austria;

[4]BioTechMed-Graz, Graz, Austria;

[5]Division of Endocrinology and Diabetes, Department of Internal Medicine, Medical University of Graz, Graz, Austria;

[6]Division of Pulmonology, Department of Internal Medicine, Medical University of Graz, Graz, Austria;

[7]Ludwig Boltzmann Institute for Lung Vascular Research, Graz, Austria;

[8]Departments of Hepatology and Gastroenterology, Aarhus University Hospital, Denmark;

[9]Department of Clinical Biochemistry, Aarhus University Hospital, Denmark;

[10]Department of Clinical Medicine, Aarhus University, Denmark;

[11]Institute of Biosciences, Life Science Center, Vilnius University, Vilnius, Lithuania.

**Corresponding Author**
Vanessa Stadlbauer, MD, PhD
Department of Internal Medicine
Division of Gastroenterology and Hepatology
Auenbruggerplatz 15
8036 Graz
Austria
Phone: 0043 316 385 82282
Email: *vanessa.stadlbauer@medunigraz.at*

**Supplementary methods**

*Neutrophil isolation*

Neutrophils were isolated from human peripheral venous blood using the Percoll method, as described before. [1] 36 ml of whole blood was gently mixed with 4 ml warm sodium citrate 3.8% solution (Roth, Karlsruhe, Germany or Sigma-Aldrich). 40 ml in total was centrifuged at 350 x g for 20 min at room temperature (RT). Supernatant (platelet-rich plasma) was carefully collected without disturbing the cells and transferred to the glass tube. 6 ml of 6% dextran solution (Serva, Heidelberg, Germany or Sigma-Aldrich) were added to the tube with pelleted cells and 0.9% sodium chloride was added up to 50 ml and the tube was gently rolled to mix the content. Suspension was left standing upright for 30 min at RT until a clear upper interface was visible. Supernatant was collected without disturbing the pellet and centrifuged (350 x g, 6 min, RT). Supernatant was discarded, cell pellet was resuspended in 3 ml 55% Percoll (Cytiva, Marlborough, Massachusetts, USA) and overlaid on a 81%/70% Percoll gradient (3 ml each). After centrifugation (720 x g, 20 min, RT) polymorphonuclear cell (PMN) layer was removed to another tube and washed twice with 50 ml of 1x Dulbecco s phosphate buffered saline (DPBS) without $Ca^{2+}$ and $Mg^{2+}$ (230 x g, 6 min, room temperature) and resuspended in assay specific medium. Neutrophils were counted with either TC20TM Automated Cell Counter or Neubauer chamber and their viability was determined with trypan blue exclusion staining. All the reagents were sterile to keep cells intact. The method yields a neutrophil purity >95% according to Diff-Quik (Thermo Scientific) stained cytocentrifuge preparations.

*ROS production of isolated neutrophils*

ROS production was measured essentially as previously described. [1] Human neutrophils right after isolation with Percoll method were resuspended in PBS++ (Dulbecco s PBS supplemented with $Ca^{2+}$ and $Mg^{2+}$, 1 g/l D-glucose and 4 mM sodium bicarbonate) to a concentration of $6.25x10^6$ cells/ml. Luminescence-grade 96 well plate (Nunc, Thermo Fisher Scientific or Greiner Bio-One) was pre-treated with 1% skimmed milk for 1 hour at RT and subsequently washed 2 times with PBS without $Ca^{2+}$ and $Mg^{2+}$ and 1 time with PBS++. 180µl of cells were mixed with 225 l of horseradish peroxidase (18.75 U/ml) and luminol (150 µM) and added to the plate containing BA/vehicle and PBS++, fMLF (added after 45 min of luminescence reading; final concentration 0.8 µM) or heat-inactivated serum-opsonized E. coli (40 bacteria/cell). Total ROS production (sum of intracellular and extracellular ROS) was measured at 37 C in real-time indirectly by chemiluminescence for 60 min in a Lumistar Omega luminescence microplate reader (BMG Labtech, Offenburg, Germany)). Relative light units per second (RLUs/s) or total RLUs integrated over the indicated periods of time were analyzed.

*Neutrophil chemotaxis*

To investigate neutrophil chemotaxis, µ-slide chemotaxis chambers (ibidi, Martinsried, Planegg, Germany) were used. Freshly isolated human neutrophils (Percoll method) were resuspended in HBSS++ buffer in a concentration of $5x10^6$cells/ml. 35 µl of cells were mixed with 75 µl collagen from rat tail (Roche, Basel, Switzerland), 5 µl of sodium bicarbonate buffer, 10 µl of 10x HBSS with $Ca^{2+}$ and $Mg^{2+}$ and phenol red. 6 µl of this suspension was added to the capillary of µ-slide and removed with the pipette from another edge of capillary. Slides were incubated for 20 min at 37 C until collagen solidify. 65 µl of HBSS++ buffer with or without 50 nM fMLF were added to the slides to create a gradient and images of neutrophils were taken every 30 s for 30 min at 37 C with the Nikon HCS microscope (Minato, Tokio, Japan). Paths of individual neutrophils were tracked and analysis of the tracks was performed using TrackMaxima plug-in in ImageJ kindly.

**Supplementary data**

Table S1: Antibodies, clones, fluorochromes, manufacturer and reference number of the antibodies used for B- and T-cell analysis

| Antibody | Clone | Fluorochrome | Manufacturer | ref# |
|---|---|---|---|---|
| CD3 | SK7 | APC-H7 | Becton Dickinson | 560176 |
| CD4 | RPA-T4 | BV605 | Becton Dickinson | 562659 |
| CD45RA | HI100 | BV786 | Becton Dickinson | 563870 |
| CD15S | CSLEX1 | BV421 | Becton Dickinson | 563912 |
| Ki-67 | B56 | AF488 | Becton Dickinson | 558616 |
| FoxP3 | 259D/C7 | PE | Becton Dickinson | 560046 |
| CD8 | RPA-T8 | BV711 | Becton Dickinson | 563677 |
| CD161 | DX12 | APC | Becton Dickinson | 550968 |
| CD127 | HIL-7R-M21 | BV510 | Becton Dickinson | 563086 |
| Fixable Viability Dye | | APC-R700 | Becton Dickinson | 564997 |
| CD25 | 2A3 | PE-Cy7 | Becton Dickinson | 335824 |
| CD39 | TU66 | PE-CF594 | Becton Dickinson | 563678 |
| CD147 | HIM6 | PerCP-Cy5.5 | Becton Dickinson | 562554 |
| CD3 | SP34-2 | AF700 | Becton Dickinson | 561805 |
| CD4 | RPA-T4 | PE-CF594 | Becton Dickinson | 562316 |
| CD27 | M-T271 | APC-H7 | Becton Dickinson | 560222 |
| CD57 | HNK-1 | FITC | Becton Dickinson | 333169 |
| PD-1 (CD279) | EH12.1 | PE | Becton Dickinson | 560795 |
| CCR7 (CD197) | 3D12 | AF647 | Becton Dickinson | 557734 |
| HLA-DR | G46-6 | BV650 | Becton Dickinson | 564231 |
| CD127 | HIL-7R-M21 | PerCpCy5.5 | Becton Dickinson | 560551 |
| CD95 | DX2 | SuperBright 600 | ThermoFisher | 63-0959-41 |
| CD38 | HIT2 | BV421 | Becton Dickinson | 562445 |
| CD28 | CD28.2 | BV510 | Becton Dickinson | 563075 |
| CD19 | SJ25C1 | PE | Becton Dickinson | 345789 |
| CD20 | L27 | APC-H7 | Becton Dickinson | 641414 |
| IgD | IA6-2 | PerCp-Cy5.5 | Becton Dickinson | 561315 |
| IgM | G20-127 | BB515 | Becton Dickinson | 564622 |
| CD24 | ML5 | BV711 | Becton Dickinson | 563401 |
| CD27 | L128 | BV786 | Becton Dickinson | 563327 |
| CD86 | 2331 (FUN-1) | PE-CF594 | Becton Dickinson | 562390 |
| CD5 | L17F12 | PE-Cy7 | Becton Dickinson | 348810 |

| Antibody | Clone | Fluorochrome | Manufacturer | ref# |
|---|---|---|---|---|
| CD38 | HIT2 | APC-R700 | Becton Dickinson | 564979 |
| CD10 | HI10a | BV510 | Becton Dickinson | 563032 |
| CD21 | B-ly4 | BV421 | Becton Dickinson | 566260 |

Table S2: Baseline characteristics of patients after severe COVID-19 infection (n=21) and after mild COVID-19 infection (n=10).

| | Severe (n=21) | Mild (n=10) | p-value |
|---|---|---|---|
| ARTIQ | | | |
| Physical upper airways | 2 (1; 4) | 3 (1; 4) | ns |
| Physical lower airways | 2 (0.5; 2.5) | 0 (0; 2) | 0.041 |
| Psychological | 0.5 (0; 1.5) | 0 (0; 2) | ns |
| Sleep | 1 (0; 2) | 0 (0; 3) | ns |
| Medicine | 0 (0; 0) | 0 (0; 0) | ns |
| Muscle pain | 0 (0; 1) | 0 (0; 0) | ns |
| Felt tired | 0 (0; 1) | 0 (0; 1) | ns |
| Staying in bed | 0 (0; 0) | 0 (0; 0) | ns |
| Cancelled work | 0 (0; 0) | 0 (0; 0) | ns |
| Cancelled leisure activities | 0 (0; 0) | 0 (0; 0) | ns |
| GIQLI | | | |
| Symptoms | 66 (61; 72) | 69 (66.5; 73.5) | 0.04 |
| Emotions | 15 (12; 16) | 15 (14; 16) | ns |
| Physical function | 19 (14; 22) | 24 (18; 27) | 0.018 |
| Social function | 14 (12; 16) | 15 (11; 16) | ns |
| Medical treatment | 4 (3; 4) | 4 (4; 4) | 0.038 |
| SF-36 | | | |
| Physical functioning | 80 (70; 95) | 100 (92.5; 100) | 0.002 |
| Physical Role | 87.5 (50; 100) | 100 (75; 100) | ns |
| Bodily pain | 92 (62; 100) | 100 (87; 100) | ns |
| General health | 62 (57; 72) | 83.5 (67; 93.5) | 0.032 |
| Vitality | 60 (45; 70) | 70 (55; 85) | ns |
| Social functioning | 93.8 (68.8; 100) | 100 (87.5; 100) | ns |
| Emotional Role | 100 (66.7; 100) | 100 (33.3; 100) | ns |
| Mental health | 84 (68; 88) | 82 (64; 92) | ns |

| | Severe (n=21) | Mild (n=10) | p-value |
|---|---|---|---|
| Recovery | | | |
| Recovery scale | 4 (3; 4) | 5 (4.5; 5) | 0.003 |
| Full recovery (yes/no) n(%) | 5 (24) / 16 (76) | 8 (80) / 2 (20) | 0.003 |
| mMRC Dyspnoea Scale | 1 (1; 1) | 0 (0; 0.5) | 0.004 |
| Dyspnoea (yes/no) n (%) | 5 (24) / 16 (76) | 0 (0) / 10 (100) | ns |
| Routine laboratory parameters | | | |
| Erythrocyte (10^12/L) | 4.69 (4.64; 4.96) | 4.92 (4.42; 5.15) | ns |
| Hemoglobin (g/dL) | 14.4 (14.2; 15) | 14.6 (13.5; 15.6) | ns |
| Hematocrit (%) | 42.8 (41.6; 43.4) | 42.3 (40.4; 46.4) | ns |
| Mean corpuscular volume (fL) | 88.2 (86.6; 89.9) | 87.2 (85.6; 90.2) | ns |
| Mean corpuscular hemoglobin (pg) | 30.8 (29.2; 31) | 30 (28.8; 31.2) | ns |
| Mean corpuscular hemoglobin concentration (g/dL) | 34 (33.3; 35.1) | 34.3 (33.1; 34.8) | ns |
| Leukocytes (10^9/L) | 6.02 (5.58; 6.55) | 5.37 (4.55; 6.06) | ns |
| Neutrophils (%) | 61 (51.5; 62) | 52.5 (46; 60.5) | ns |
| Eosinophils (%) | 2 (2; 3) | 2.5 (1; 5) | ns |
| Monocytes (%) | 9 (8; 9) | 8 (7; 10) | ns |
| Basophils % | 1 (1; 1) | 1 (0.1; 1) | ns |
| Lymphocytes (%) | 28.5 (27; 31.5) | 33 (29; 42) | ns |
| Thrombocyte (10^9/L) | 224 (202; 268) | 260 (238; 303) | ns |
| Mean platelet volume (fL) | 10.4 (10; 11) | 10.4 (9.9; 10.9) | ns |
| Prothrombin time (%) | 113 (108; 116) | 106 (101; 119) | ns |
| Prothrombin time international normalized ratio | 0.935 (0.92; 0.96) | 0.965 (0.9; 0.99) | ns |
| Activated partial thromboplastin time (s) | 30.7 (28.8; 34) | 30.5 (28.1; 32.9) | ns |
| Fibrinogen (mg/dL) | 282 (249; 312) | 250 (219; 324) | ns |
| Triglyceride (mg/dL) | 114 (79.5; 182) | 73.5 (51; 132) | ns |
| Cholesterol (mg/dL) | 190 (160; 217) | 202 (178; 242) | ns |
| Glucose (mg/dL) | 100 (95; 108) | 85.5 (78; 94) | 0.003 |
| Creatinine (mg/dL) | 0.88 (0.81; 1) | 0.775 (0.73; 0.92) | ns |
| Glomerular filtration rate (ml/min) | 87.2 (81.3; 95.9) | 103 (90.8; 115) | 0.031 |
| Sodium (mmol/L) | 140 (139; 141) | 140 (138; 141) | ns |
| Potassium (mmol/L) | 4.1 (4; 4.3) | 4.05 (3.8; 4.65) | ns |

| | Severe (n=21) | Mild (n=10) | p-value |
|---|---|---|---|
| Chloride (mmol/l) | 103 (102; 104) | 102 (101; 102) | 0.025 |
| Calcium (mmol/L) | 2.37 (2.35; 2.39) | 2.44 (2.38; 2.54) | 0.014 |
| Total protein (g/dL) | 7.3 (7; 7.4) | 7.6 (7.55; 8) | 0.004 |
| Albumin (g/dL) | 4.6 (4.5; 4.7) | 4.7 (4.65; 5) | 0.007 |
| Bilirubin direct (mg/dL) | 0.2 (0.15; 0.255) | 0.255 (0.14; 0.325) | ns |
| Bilirubin total (mg/dL) | 0.51 (0.41; 0.59) | 0.625 (0.33; 0.86) | ns |
| Alkaline Phosphatase (U/L) | 62.5 (56; 71.5) | 62.5 (51; 71) | ns |
| Aspartate aminotransferase (U/L) | 20 (18; 25) | 23.5 (18; 31) | ns |
| Alanine aminotransferase (U/L) | 22 (16; 35) | 18.5 (13.5; 30) | ns |
| Gamma glutamyltransferase (U/l) | 27 (18.5; 30.5) | 19.5 (13; 41) | ns |
| Cholinesterase (U/L) | 8540 (7910; 8780) | 8020 (7010; 8710) | ns |
| C-reactive protein (mg/L) | 1.7 (1; 3.1) | 0.95 (0.6; 3.4) | ns |
| Lactate dehydrogenase (U/L) | 187 (180; 206) | 168 (144; 206) | ns |
| Gut permeability and bacterial translocation | | | |
| Zonulin (ng/mL) | 32 (26.7; 42.3) | 32.7 (22.8; 37.6) | ns |
| DAO (U/mL) | 12.5 (10.9; 14.4) | 7.85 (4.47; 15) | 0.035 |
| LBP (ng/mL) | 13.2 (9.6; 16) | 12.2 (10.3; 14) | ns |
| sCD14 ( g/mL) | 1.32 (1.21; 1.48) | 1.68 (1.26; 1.75) | ns |
| Macrophage activation markers | | | |
| sCD163 (mg/l) | 1.74 (1.58; 2.37) | 1.57 (1.38; 2.02) | ns |
| sMR (mg/l) | 0.24 (0.202; 0.258) | 0.178 (0.145; 0.212) | 0.008 |
| Neutrophil function | | | |
| Chemotaxis Directedness | 0.416 (0.368; 0.457) | 0.452 (0.429; 0.476) | 0.035 |
| Chemotactic Efficiency Index (CEI) | -0.003 (-0.021; 0.028) | 0.009 (-0.025; 0.043) | ns |
| Phosphate-buffered saline (PBS) | 9300 (4420; 15000) | 11100 (4370; 25500) | ns |
| N-Formylmethionyl-leucyl-phenylalanine (fMLF) | 24500 (12000; 48300) | 26100 (10300; 52300) | ns |
| Escherichia coli | 53200 (44500; 64400) | 82900 (69000; 104000) | 0.009 |
| Phorbol myristate acetate (PMA) | 177000 (157000; 199000) | 187000 (175000; 217000) | ns |

Acute Respiratory Tract Infection Questionnaire (ARTIQ): a lower score indicates a better outcome. Gastrointestinal Quality of Life Index (GIQLI) questionnaire score ranges from 0 to 144, and a higher score indicates a better gastrointestinal quality of life. 36-Item Short Form Survey (SF-36) score ranges between 0 and 100, a higher score indicates a better health-related quality of life. Recovery scale ranges from 1 to 5, a higher score indicates a better outcome. The modified Medical Research Council

(mMRC) Dyspnoea Scale score ranges from 0 to 4, a lower score indicates a better outcome. Data are shown as median and 95% confidence interval unless stated otherwise; ns not significant

Table S3: Read counts per sample for each group and time point (data is given as mean and standard deviation)

| Group | Timepoint | Raw reads per sample | | High quality reads per sample | |
|---|---|---|---|---|---|
| Mild | | 45863.8 | (10558.3) | 28675.1 | (7957.4) |
| Placebo | Baseline | 36300.5 | (11101.7) | 19065.8 | (5698.7) |
| Placebo | 3 months | 32288.9 | (10726.5) | 17037.9 | (5586.6) |
| Placebo | 6 months | 38385.2 | (10436.0) | 20316.3 | (5669.5) |
| Probiotic | Baseline | 31186.9 | (10017.4) | 15919.0 | (3979.7) |
| Probiotic | 3 months | 41369.1 | (13359.1) | 21657.1 | (6406.7) |
| Probiotic | 6 months | 41138.0 | (8704.3) | 22893.6 | (6638.2) |

Table S4: Serum metabolomics including inflammatory proteins, small molecular metabolites and lipoproteins for patients after severe and mild COVID-19 disease. Values are given as median (95% confidence interval)

| Parameter | Severe (n=20) | Mild (n=10) | p-value |
|---|---|---|---|
| Glycoprotein signal A (p.d.u) | 0.92 (0.84; 0.965) | 0.88 (0.795; 0.935) | ns |
| Glycoprotein signal B (p.d.u) | 0.365 (0.335; 0.39) | 0.36 (0.32; 0.38) | ns |
| Total glycoprotein signal | 1.29 (1.17; 1.35) | 1.25 (1.13; 1.31) | ns |
| Supramolecular phospholipidcomposite (p.d.u) | 2.22 (2.04; 2.48) | 2.56 (2.27; 3.28) | ns |
| Glycoprotein to supramolecular phospholipidcomposite ratio | 0.575 (0.515; 0.63) | 0.44 (0.38; 0.52) | 0.005* |
| Glycoprotein signal A to Glycoprotein signal B ratio | 2.5 (2.46; 2.53) | 2.47 (2.42; 2.53) | ns |
| Ethanol (mmol/L) | 0 (0; 0.08) | 0.45 (0; 0.89) | 0.009* |
| Alanine (mmol/L) | 0.38 (0.32; 0.415) | 0.43 (0.33; 0.54) | ns |
| Asparagine (mmol/L) | 0 (0; 0) | 0 (0; 0) | ns |
| Creatine (mmol/L) | 0.025 (0.015; 0.08) | 0.015 (0; 0.03) | ns |
| Creatinine (mmol/L) | 0.115 (0.085; 0.13) | 0.1 (0.08; 0.13) | ns |
| Glutamic acid (mmol/L) | 0.195 (0.165; 0.24) | 0.17 (0.13; 0.24) | ns |
| Glutamine (mmol/L) | 0.58 (0.545; 0.68) | 0.78 (0.6; 0.86) | 0.025* |
| Glycine (mmol/L) | 0.365 (0.25; 0.465) | 0.23 (0.18; 0.455) | ns |
| Histidine (mmol/L) | 0.07 (0.065; 0.08) | 0.075 (0.06; 0.09) | ns |
| Isoleucine (mmol/L) | 0.06 (0.055; 0.075) | 0.05 (0.045; 0.06) | ns |
| Leucine (mmol/L) | 0.1 (0.09; 0.12) | 0.095 (0.06; 0.1) | ns |
| Lysine (mmol/L) | 0.14 (0.11; 0.17) | 0.13 (0.11; 0.18) | ns |

| Parameter | Severe (n=20) | Mild (n=10) | p-value |
|---|---|---|---|
| Methionine (mmol/L) | 0.07 (0.06; 0.07) | 0.065 (0; 0.08) | ns |
| Ornithine (mmol/L) | 0 (0; 0.04) | 0 (0; 0.04) | ns |
| Phenylalanine (mmol/L) | 0.13 (0.11; 0.15) | 0.07 (0.055; 0.1) | 0.001* |
| Proline (mmol/L) | 0 (0; 0.47) | 0.25 (0; 0.64) | ns |
| Threonine (mmol/L) | 0 (0; 0.02) | 0 (0; 0) | ns |
| Tyrosine (mmol/L) | 0.05 (0.04; 0.06) | 0.05 (0.03; 0.06) | ns |
| Valine (mmol/L) | 0.215 (0.19; 0.245) | 0.2 (0.185; 0.225) | ns |
| Acetic acid (mmol/L) | 0.035 (0.025; 0.04) | 0.035 (0.02; 0.06) | ns |
| Citric acid (mmol/L) | 0.08 (0.065; 0.11) | 0.03 (0; 0.085) | 0.019* |
| Formic acid (mmol/L) | 0.06 (0.05; 0.07) | 0.05 (0.045; 0.065) | ns |
| Lactic acid (mmol/L) | 2 (1.75; 2.3) | 1.75 (1.4; 2.2) | ns |
| Succinic acid (mmol/L) | 0.01 (0; 0.045) | 0 (0; 0.01) | ns |
| 3-Hydroxybutyric acid (mmol/L) | 0.275 (0.08; 0.925) | 0.215 (0.1; 0.48) | ns |
| Acetoacetic acid (mmol/L) | 0.025 (0; 0.165) | 0 (0; 0.005) | 0.016* |
| Acetone (mmol/L) | 0.025 (0; 0.065) | 0.035 (0.01; 0.08) | ns |
| Pyruvic acid (mmol/L) | 0.08 (0.055; 0.115) | 0.07 (0.04; 0.1) | ns |
| Glucose (mmol/L) | 7.05 (6.15; 8.15) | 6.05 (5.5; 11.6) | ns |
| Dimethylsulfone (mmol/L) | 0 (0; 0) | 0 (0; 0.005) | ns |
| Total triglycerides (mg/dL) | 114 (73.7; 197) | 73.6 (46.3; 129) | 0.044 |
| Total cholesterol (mg/dL) | 215 (170; 234) | 216 (191; 267) | ns |
| LDL cholesterol (mg/dL) | 115 (94.7; 131) | 123 (109; 149) | ns |
| HDL cholesterol (mg/dL) | 58.8 (50.4; 63.6) | 71.7 (65.6; 81) | 0.001* |
| Total Apo-A1 (mg/dL) | 159 (145; 177) | 180 (157; 200) | 0.044 |
| Total Apo-A2 (mg/dL) | 36.4 (33.6; 39.5) | 43.5 (37.1; 48.6) | 0.017* |
| Total Apo-B100 (mg/dL) | 91.4 (72.1; 110) | 80.7 (71.9; 94.4) | ns |
| LDL cholesterol to HDL cholerstol ratio | 1.86 (1.48; 2.61) | 1.64 (1.4; 2.26) | ns |
| Apo-B100 to Apo-A1 ratio | 0.485 (0.415; 0.755) | 0.455 (0.4; 0.55) | ns |
| Total particle number (nmol/L) | 1660 (1310; 1990) | 1470 (1310; 1750) | ns |
| VLDL particle number (nmol/L) | 152 (108; 233) | 72.3 (44.9; 138) | 0.011* |
| IDL particle number (nmol/L) | 104 (75.3; 132) | 79.5 (38.9; 110) | ns |
| LDL particle number (nmol/L) | 1410 (1060; 1650) | 1300 (1190; 1610) | ns |
| LDL-1 particle number (nmol/L) | 169 (164; 197) | 227 (167; 281) | ns |
| LDL-2 particle number (nmol/L) | 128 (109; 140) | 212 (170; 249) | 0.001* |
| LDL-3 particle number (nmol/L) | 158 (134; 215) | 233 (189; 278) | 0.035 |
| LDL-4 particle number (nmol/L) | 204 (187; 281) | 217 (145; 279) | ns |

| Parameter | Severe (n=20) | Mild (n=10) | p-value |
|---|---|---|---|
| LDL-5 particle number (nmol/L) | 259 (200; 400) | 199 (162; 270) | ns |
| LDL-6 particle number (nmol/L) | 319 (236; 491) | 318 (286; 340) | ns |
| VLDL triglycerides (mg/dL) | 75.4 (45.4; 129) | 41.6 (20.9; 88) | ns |
| IDL triglycerides (mg/dL) | 11.3 (4.73; 22.3) | 5.48 (0.87; 15) | ns |
| LDL triglycerides (mg/dL) | 23 (18.2; 25.6) | 16.2 (13.2; 24) | ns |
| VLDL cholesterol (mg/dL) | 16.3 (12.2; 34.9) | 8.86 (4.75; 20.5) | 0.019* |
| IDL cholesterol (mg/dL) | 11.8 (8.62; 20.5) | 8.2 (3.71; 13.7) | ns |
| VLDL free cholesterol (mg/dL) | 7.75 (5.8; 14.3) | 3.78 (2.45; 8.14) | 0.02* |
| IDL free cholesterol (mg/dL) | 3.48 (2.51; 5.98) | 2.42 (1.38; 4.42) | ns |
| LDL free cholesterol (mg/dL) | 31.8 (28.4; 38.6) | 36.9 (32.1; 42.7) | ns |
| HDL free cholesterol (mg/dL) | 11.7 (10.4; 13.8) | 15.3 (14.1; 18.7) | 0.003* |
| VLDL phospholipids (mg/dL) | 19.7 (13.6; 32.7) | 11.4 (6.54; 19.8) | 0.022* |
| IDL phospholipids (mg/dL) | 8.02 (6.04; 12.2) | 5.69 (4.21; 9.99) | ns |
| LDL phospholipids (mg/dL) | 67.1 (55.7; 74.8) | 69.5 (62.8; 81.6) | ns |
| HDL Apo-A1 (mg/dL) | 157 (143; 177) | 179 (160; 200) | 0.022* |
| HDL Apo-A2 (mg/dL) | 36.4 (34.1; 39) | 42.6 (36.9; 47.8) | 0.023* |
| VLDL Apo-B100 (mg/dL) | 8.34 (5.96; 12.8) | 3.98 (2.47; 7.57) | 0.011* |
| IDL Apo-B100 (mg/dL) | 5.7 (4.14; 7.26) | 4.37 (2.14; 6.04) | ns |
| LDL Apo-B100 (mg/dL) | 77.8 (58.5; 90.5) | 71.8 (66.5; 88.6) | ns |
| VLDL-1 triglycerides (mg/dL) | 36.3 (21.2; 55.9) | 13.3 (3.61; 41.3) | ns |
| VLDL-2 triglycerides (mg/dL) | 12.6 (6.8; 21) | 7.48 (3.02; 14.6) | ns |
| VLDL-3 triglycerides (mg/dL) | 12.3 (6.89; 19.1) | 6.65 (4.12; 13.1) | 0.035 |
| VLDL-4 triglycerides (mg/dL) | 10.2 (6.5; 13) | 4.55 (3.2; 9.46) | 0.022* |
| VLDL-5 triglycerides (mg/dL) | 2.56 (2.02; 3.08) | 1.72 (1.14; 2.28) | 0.021* |
| VLDL-1 cholesterol (mg/dL) | 4.76 (2.66; 13.9) | 1.4 (0.33; 5.98) | 0.028* |
| VLDL-2 cholesterol (mg/dL) | 2.38 (1.76; 5.92) | 1.86 (0.63; 3.28) | ns |
| VLDL-3 cholesterol (mg/dL) | 3.43 (1.99; 7.09) | 2.04 (1.08; 4.3) | ns |
| VLDL-4 cholesterol (mg/dL) | 4.54 (2.92; 8.24) | 2.18 (1.36; 4.24) | 0.013* |
| VLDL-5 cholesterol (mg/dL) | 1.18 (0.645; 1.62) | 0.395 (0.12; 1.05) | 0.033 |
| VLDL-1 free cholesterol (mg/dL) | 2.16 (1.06; 4.89) | 0.895 (0.13; 3.18) | ns |
| VLDL-2 free cholesterol (mg/dL) | 1.17 (0.62; 2.52) | 0.56 (0.125; 1.48) | 0.049 |
| VLDL-3 free cholesterol (mg/dL) | 1.54 (0.78; 2.95) | 0.53 (0.2; 1.58) | 0.024* |
| VLDL-4 free cholesterol (mg/dL) | 1.97 (1.02; 3.32) | 0.695 (0.18; 2.01) | 0.018* |
| VLDL-5 free cholesterol (mg/dL) | 0.26 (0.075; 0.675) | 0.02 (0; 0.39) | ns |
| VLDL-1 phospholipids (mg/dL) | 6.28 (3.87; 10.9) | 2.56 (1.21; 6.72) | 0.039 |
| VLDL-2 phospholipids (mg/dL) | 3.38 (1.96; 5.67) | 2.24 (1.12; 3.92) | ns |

| Parameter | Severe (n=20) | Mild (n=10) | p-value |
|---|---|---|---|
| VLDL-3 phospholipids (mg/dL) | 4.32 (2.48; 6.58) | 2.58 (1.21; 4.36) | 0.048 |
| VLDL-4 phospholipids (mg/dL) | 5 (3.42; 6.74) | 2.23 (1.65; 4.5) | 0.013* |
| VLDL-5 phospholipids (mg/dL) | 1.5 (1.1; 2.06) | 0.54 (0.315; 1.38) | 0.033 |
| LDL-1 triglycerides (mg/dL) | 6.04 (4.57; 7.11) | 4.47 (3.18; 6.91) | ns |
| LDL-2 triglycerides (mg/dL) | 2.3 (2.05; 2.46) | 2.6 (1.86; 3.31) | ns |
| LDL-3 triglycerides (mg/dL) | 2.31 (1.98; 2.82) | 2.14 (1.66; 2.92) | ns |
| LDL-4 triglycerides (mg/dL) | 3.08 (2.36; 3.9) | 1.68 (1.28; 3.41) | 0.028* |
| LDL-5 triglycerides (mg/dL) | 3.16 (2.42; 4.79) | 1.64 (1.4; 3.41) | 0.019* |
| LDL-6 triglycerides (mg/dL) | 3.89 (2.76; 5.6) | 3.3 (2.96; 3.95) | ns |
| LDL-1 cholesterol (mg/dL) | 16.9 (15.3; 20.1) | 24.2 (18; 29.6) | ns |
| LDL-2 cholesterol (mg/dL) | 12.7 (8.75; 13.6) | 22.6 (17.2; 25.4) | 0.001* |
| LDL-3 cholesterol (mg/dL) | 15 (11.6; 19.5) | 22.4 (18.7; 26.8) | 0.015* |
| LDL-4 cholesterol (mg/dL) | 17.2 (15.2; 23.5) | 19.6 (14.3; 25) | ns |
| LDL-5 cholesterol (mg/dL) | 20.6 (15.4; 30.2) | 16.6 (13.2; 21.5) | ns |
| LDL-6 cholesterol (mg/dL) | 22.4 (16.7; 33.2) | 22.4 (19.3; 23.7) | ns |
| LDL-1 free cholesterol (mg/dL) | 5.7 (5.28; 6.44) | 7.75 (5.74; 9.33) | ns |
| LDL-2 free cholesterol (mg/dL) | 3.85 (3.32; 5.04) | 7.3 (6.32; 8.22) | 0.001* |
| LDL-3 free cholesterol (mg/dL) | 5.24 (4.05; 5.8) | 7.56 (6.18; 8.44) | 0.003* |
| LDL-4 free cholesterol (mg/dL) | 5.14 (4.88; 6.62) | 5.98 (4.59; 7.45) | ns |
| LDL-5 free cholesterol (mg/dL) | 6.1 (4.69; 7.61) | 4.78 (4.45; 6.48) | ns |
| LDL-6 free cholesterol (mg/dL) | 6.28 (4.64; 8.06) | 6.38 (5.61; 6.95) | ns |
| LDL-1 phospholipids (mg/dL) | 10.4 (9.83; 11.9) | 13.8 (10.8; 17.4) | ns |
| LDL-2 phospholipids (mg/dL) | 7.38 (5.38; 8.07) | 12.5 (10.2; 13.8) | 0.001* |
| LDL-3 phospholipids (mg/dL) | 8.78 (7.16; 10.7) | 12.9 (10.5; 14.8) | 0.022* |
| LDL-4 phospholipids (mg/dL) | 9.76 (8.75; 13.1) | 10.9 (8.14; 13.7) | ns |
| LDL-5 phospholipids (mg/dL) | 11.3 (8.87; 16.3) | 8.98 (7.46; 12.3) | ns |
| LDL-6 phospholipids (mg/dL) | 12.8 (10.1; 18.2) | 12.7 (11.8; 13.7) | ns |
| LDL-1 Apo-B100 (mg/dL) | 9.28 (8.99; 10.8) | 12.5 (9.2; 15.4) | ns |
| LDL-2 Apo-B100 (mg/dL) | 7.06 (5.98; 7.68) | 11.7 (9.37; 13.7) | 0.002* |
| LDL-3 Apo-B100 (mg/dL) | 8.72 (7.4; 11.8) | 12.8 (10.4; 15.3) | 0.035 |
| LDL-4 Apo-B100 (mg/dL) | 11.2 (10.3; 15.4) | 11.9 (8.15; 15.3) | ns |
| LDL-5 Apo-B100 (mg/dL) | 14.2 (11; 22) | 10.9 (8.92; 14.8) | ns |
| LDL-6 Apo-B100 (mg/dL) | 17.6 (13.1; 27) | 17.5 (15.7; 18.7) | ns |
| HDL-1 triglycerides (mg/dL) | 2.83 (2.43; 3.48) | 2 (1.27; 4.77) | ns |
| HDL-2 triglycerides (mg/dL) | 1.72 (1.55; 1.98) | 1.13 (0.95; 2.63) | ns |
| HDL-3 triglycerides (mg/dL) | 2.08 (1.98; 2.96) | 1.92 (1.25; 3.2) | ns |

| Parameter | Severe (n=20) | Mild (n=10) | p-value |
|---|---|---|---|
| HDL-4 triglycerides (mg/dL) | 4.3 (3.68; 4.94) | 3.4 (2.66; 4.1) | 0.045 |
| HDL-1 cholesterol (mg/dL) | 14.6 (10.2; 18.3) | 20.5 (17.3; 30.9) | 0.017* |
| HDL-2 cholesterol (mg/dL) | 8.68 (7.12; 9.89) | 11.2 (9.87; 13.4) | 0.009* |
| HDL-3 cholesterol (mg/dL) | 11.9 (10.5; 12.9) | 14.1 (12.1; 15.8) | 0.006* |
| HDL-4 cholesterol (mg/dL) | 20.7 (19; 23.5) | 24.5 (23.2; 29.2) | 0.039 |
| HDL-1 free cholesterol (mg/dL) | 3.58 (3.04; 4.47) | 5.82 (5.22; 7.9) | 0.001* |
| HDL-2 free cholesterol (mg/dL) | 2.06 (1.69; 2.49) | 2.88 (2.54; 3.5) | 0.003* |
| HDL-3 free cholesterol (mg/dL) | 2.53 (2.26; 2.79) | 3.34 (2.83; 4.22) | 0.006* |
| HDL-4 free cholesterol (mg/dL) | 4.53 (4.06; 4.84) | 5.15 (4.37; 6.52) | ns |
| HDL-1 phospholipids (mg/dL) | 17.2 (13.3; 22.4) | 25.7 (20.3; 38.2) | 0.022* |
| HDL-2 phospholipids (mg/dL) | 13.7 (11.7; 16.1) | 16.7 (14.3; 20.5) | 0.028* |
| HDL-3 phospholipids (mg/dL) | 18.4 (16.3; 22) | 21.8 (18.2; 25.6) | ns |
| HDL-4 phospholipids (mg/dL) | 31.1 (28.9; 33) | 33.8 (30.1; 39.9) | ns |
| HDL-1 Apo-A1 (mg/dL) | 19.5 (13.6; 28.3) | 31.1 (24.3; 46.5) | 0.035 |
| HDL-2 Apo-A1 (mg/dL) | 20.2 (17.8; 22.1) | 23 (21; 29.3) | 0.035 |
| HDL-3 Apo-A1 (mg/dL) | 29.6 (26.4; 35.1) | 34.4 (28.6; 40.1) | ns |
| HDL-4 Apo-A1 (mg/dL) | 83 (78.3; 88) | 88.5 (81; 99.7) | ns |
| HDL-1 Apo-A2 (mg/dL) | 2.12 (2.01; 2.9) | 3.03 (2.59; 5.72) | 0.023* |
| HDL-2 Apo-A2 (mg/dL) | 3.87 (3.59; 4.42) | 5.1 (3.76; 6.62) | ns |
| HDL-3 Apo-A2 (mg/dL) | 7.62 (7.07; 8.62) | 9.75 (7.25; 10.9) | ns |
| HDL-4 Apo-A2 (mg/dL) | 21.8 (20.1; 24.1) | 25.2 (21.4; 27.3) | ns |
| Total particle free cholesterol (mg/dL) | 62.6 (49; 68.7) | 59.6 (51.4; 74.2) | ns |
| Total particle phospholipids (mg/dL) | 183 (174; 193) | 185 (165; 228) | ns |

p.d.u - procedure defined units, VLDL – very low density lipoprotein, LDL – low density lipoprotein, IDL – intermediate density lipoprotein, HDL – high density lipoprotein, * - remained significant after Benjamini Hochberg correction; Data are shown as median and 95% confidence interval unless stated otherwise; ns not significant

Table S5: Stool metabolites in patients after mild and severe COVID-19 disease in arbitrary units. Data are given as median (95% confidence interval)

| Parameter | Severe (n=21) | Mild (n=10) | p-value |
|---|---|---|---|
| Chenodeoxycholic acid | 0.384 (0.319; 0.429) | 0.274 (0.191; 0.435) | ns |
| Glycocholic acid & derivatives | 0.305 (0.259; 0.347) | 0.175 (0.125; 0.296) | 0.004 |
| Cholic acid | 0.245 (0.232; 0.286) | 0.217 (0.187; 0.289) | ns |
| Capric acid | 81.1 (74.1; 86.4) | 81.4 (69; 98.1) | ns |
| Butyric acid | 4.27 (4.02; 4.92) | 4.82 (4.12; 5.49) | ns |
| Leucine | 2.46 (2.33; 2.75) | 2.47 (2.08; 2.78) | ns |

| Parameter | Severe (n=21) | Mild (n=10) | p-value |
|---|---|---|---|
| Valine | 1.18 (1.11; 1.41) | 0.992 (0.789; 1.27) | 0.039 |
| Isoleucine | 0.364 (0.322; 0.396) | 0.293 (0.25; 0.382) | ns |
| Propionic acid | 1.12 (0.958; 1.39) | 1.11 (0.893; 1.4) | ns |
| Propylene glycol | 0.75 (0.721; 0.768) | 0.767 (0.698; 0.83) | ns |
| Isopropyl alcohol | 2.26 (2.17; 2.36) | 2.37 (2.14; 2.54) | ns |
| Valeric acid | 99 (85.4; 110) | 97.6 (80.2; 112) | ns |
| Lactic acid | 58.5 (49.4; 60.2) | 56.5 (44.7; 65.6) | ns |
| Alanine | 1.28 (1.25; 1.38) | 1.22 (1.16; 1.3) | ns |
| Lysine | 1 (0.915; 1.37) | 1.04 (0.906; 1.25) | ns |
| Acetic acid | 19.7 (18.5; 26) | 22.4 (17.7; 26.3) | ns |
| 5-Aminopentanoic acid | 0.427 (0.36; 0.464) | 0.372 (0.316; 0.421) | ns |
| Glutamic acid | 2.19 (2.02; 2.31) | 2.29 (2.14; 2.96) | ns |
| Succinic acid | 42 (38.2; 44.4) | 39.3 (32.8; 47.2) | ns |
| beta-Alanine | 0.372 (0.361; 0.389) | 0.37 (0.346; 0.411) | ns |
| Methylamine | 0.143 (0.132; 0.159) | 0.16 (0.137; 0.187) | ns |
| Aspartic acid | 0.258 (0.24; 0.288) | 0.252 (0.228; 0.292) | ns |
| Sarcosine | 0.316 (0.267; 0.34) | 0.287 (0.25; 0.358) | ns |
| Trimethylamine | 14.1 (12.7; 15.7) | 14.6 (13.8; 17.7) | ns |
| Asparagine | 0.46 (0.435; 0.478) | 0.423 (0.418; 0.439) | 0.043 |
| Malonic acid | 3.38 (2.97; 3.63) | 3.44 (2.67; 3.99) | ns |
| Choline | 0.663 (0.61; 0.86) | 0.712 (0.559; 0.819) | ns |
| Betaine | 0.754 (0.687; 0.782) | 0.572 (0.497; 0.762) | ns |
| Taurine | 2.48 (2.26; 2.57) | 2.26 (1.9; 2.74) | ns |
| Phenylacetic acid | 1.2 (1.17; 1.33) | 1.24 (1.13; 1.32) | ns |
| Glycine | 0.801 (0.746; 0.874) | 0.876 (0.824; 1.05) | 0.048 |
| Glycerol | 1.52 (1.35; 1.74) | 1.79 (1.59; 2.13) | 0.039 |
| Fructose | 0.309 (0.294; 0.35) | 0.298 (0.267; 0.369) | ns |
| Proline | 0.315 (0.256; 0.387) | 0.313 (0.273; 0.406) | ns |
| Lactulose | 0.065 (0.0513; 0.0703) | 0.077 (0.0691; 0.103) | 0.025 |
| Glucose | 1.61 (1.46; 1.74) | 1.59 (1.35; 1.97) | ns |
| Galactose | 0.254 (0.176; 0.354) | 0.239 (0.196; 0.597) | ns |
| Sucrose | 0.118 (0.111; 0.133) | 0.125 (0.104; 0.145) | ns |
| Uracil | -0.0375 (-0.0446; -0.026) | -0.0306 (-0.0385; -0.0123) | ns |
| Fumaric acid | -0.000611 (-0.00328; 0.0013) | 0.000675 (-0.00337; 0.0038) | ns |

| Parameter | Severe (n=21) | Mild (n=10) | p-value |
|---|---|---|---|
| Deoxyuridine | -0.0122 (-0.0174; -0.00235) | -0.00467 (-0.0105; 0.00391) | ns |
| 3,4-Dihydroxybenzeneacetic acid | 0.0407 (0.0299; 0.0573) | 0.0573 (0.0274; 0.0863) | ns |
| Tyrosine | 0.0786 (0.0492; 0.0979) | 0.0669 (0.0374; 0.138) | ns |
| Gallic acid | 0.0377 (0.0302; 0.0563) | 0.0457 (0.0276; 0.0653) | ns |
| Histidine | 0.0804 (0.0637; 0.0927) | 0.081 (0.0612; 0.111) | ns |
| p-Cresol | 0.109 (0.0877; 0.138) | 0.112 (0.0701; 0.176) | ns |
| Phenylalanine | 0.481 (0.425; 0.515) | 0.499 (0.433; 0.593) | ns |
| Tryptophan | 0.0069 (0.00473; 0.0145) | 0.0101 (0.00638; 0.0209) | ns |
| Xanthine | 0.000444 (-0.00257; 0.0035) | -0.00107 (-0.00566; 0.0119) | ns |
| Hypoxanthine | -0.00125 (-0.00662; 0.0033) | -0.00438 (-0.00845; 0.00444) | ns |
| Formic acid | 0.0131 (0.0102; 0.0175) | 0.0131 (0.00764; 0.0228) | ns |
| Nicotinic acid | -0.0089 (-0.0177; -0.00159) | -0.0135 (-0.0184; 0.00316) | ns |

Data are shown as median and 95% confidence interval unless stated otherwise; ns not significant

Table S6: T- and B-cell populations in patients after mild and severe COVID-19 infection. Values are given as median and 95% confidence interval.

| | Severe (n=21) | Mild (n=10) | p-value |
|---|---|---|---|
| % CD3+CD8+ T cells in lymphocytes | 19.9 (16.4; 23.7) | 21 (14.4; 24.6) | ns |
| % CD161 in CD3+CD8 T cells | 8.59 (6.65; 11.9) | 11.6 (9.29; 21.5) | 0.04 |
| % CD3+CD4+ T cells in lymphocytes | 48.5 (42.5; 55) | 36.7 (33.6; 48.6) | 0.013 |
| % CD45RA-Ki67+ in CD3+CD4+ T cells | 1.65 (1.03; 1.96) | 0.81 (0.57; 1.39) | 0.028 |
| % CD45RA+Ki67- in CD3+CD4+ T cells | 45.3 (38.2; 51.5) | 47.5 (41.4; 64.8) | ns |
| % CD45RA-CD39+ in CD3+CD4+ T cells | 4.83 (3.02; 5.64) | 5.08 (3.02; 12.4) | ns |
| % CD45RA+CD39- in CD3+CD4+ T cells | 43.6 (36.8; 50.8) | 43.4 (31.8; 63.6) | ns |
| % CD25+FoxP3+ Tregs in CD3+CD4+ T cells | 4.23 (3.46; 5.08) | 3.56 (1.73; 5) | ns |
| % CD147high in Tregs | 8.38 (6.49; 10.1) | 13.8 (9.95; 18.5) | ns |
| % effector Tregs in Tregs | 20.2 (17.4; 29.9) | 25.1 (17.6; 36.8) | ns |
| % naive Tregs in Tregs | 35.7 (33; 48.5) | 28.6 (22.5; 40.6) | ns |

| | Severe (n=21) | Mild (n=10) | p-value |
|---|---|---|---|
| % CM cells in CD4+ T cells | 11.4 (9.23; 13.8) | 18.2 (13.4; 23.5) | 0.024 |
| % EM cells in CD4+ T cells | 40.2 (34.1; 45.6) | 24 (16.9; 30.3) | 0.009 |
| % NAIVE cells in CD4+ T cells | 21.2 (17.2; 34) | 42.2 (30.8; 55.6) | 0.031 |
| % TEMRA cells in CD4+ T cells | 20.8 (15.2; 23.7) | 11.2 (8.41; 16.5) | 0.035 |
| % CM cells in CD8+ T cells | 3.88 (1.95; 6.81) | 3.06 (2.46; 6.03) | ns |
| % EM cells in CD8+ T cells | 24.6 (20.7; 34.4) | 29.6 (24.6; 49.8) | ns |
| % NAIVE cells in CD8+ T cells | 17.6 (10.9; 36.4) | 25 (18.9; 35.2) | ns |
| % TEMRA cells in CD8+ T cells | 37.1 (26.4; 50.6) | 36.7 (23.6; 41) | ns |
| % CD19+CD20+ B cells in lymphocytes | 7.97 (6.24; 10.3) | 6.07 (5.14; 9.85) | ns |
| % activated B cells in B cells | 1.52 (1.33; 1.99) | 2.38 (1.39; 3.57) | ns |
| % CCR7+ B cells in B cells | 7.73 (5.56; 14.3) | 6.12 (3.99; 12) | ns |
| % naive B cells in B cells | 72.3 (63.1; 78.6) | 65.9 (54.8; 69) | ns |
| % marginal Zone B cells in B cells | 4.99 (3.92; 7.61) | 11.3 (5.86; 17.4) | 0.021 |
| % Non-switched memory B cells in B cells | 10.9 (9.2; 15.2) | 6.66 (5.74; 12.4) | ns |
| % Class-switched memory B cells in B cells | 12.5 (11; 17) | 14.6 (11.5; 17) | ns |
| % IgD post-switched B cells in B cells | 11 (10.1; 14.7) | 12.8 (9.74; 16.4) | ns |
| % IgM only memory B cells in B cells | 5.56 (2.99; 8.28) | 8.33 (4.02; 13.1) | ns |

Data are shown as median and 95% confidence interval unless stated otherwise; ns not significant. Populations are named according to the applied gating strategy (e.g. CD3CD8 double positive cells in lymphocytes)

Table S7: Baseline and 3 months of treatment values, and changes in ARTIQ, GIQLI and SF-36 questionnaire, recovery- and mMRC score parameters; data are given as median (95% confidence interval).

| | Probiotic | | | Placebo | | | p-value (between changes) |
|---|---|---|---|---|---|---|---|
| | Baseline | 3 months | Change | Baseline | 3 months | Change | |
| ARTIQ | | | | | | | |
| Physical upper airways | 4 (1.5; 9) | 3.5 (2; 8.5) | 0 (-1; 3) | 1 (1; 3.5) | 1.5 (0.5; 3.5) | 0 (-2; 1) | ns |
| Physical lower airways | 3 (0.5; 4.5) | 1.5 (0.5; 3.5) | -1 (-3; 0) | 1.5 (0; 2) | 0.5 (0; 2) | 0 (-2; 1) | ns |
| Psychological | 2 (0; 5) | 1.5 (0.5; 2) | -2 (-4; 0) | 0 (0; 1) | 0 (0; 1) | 0 (0; 0) | 0.022 |
| Sleep | 1 (0; 2) | 2 (0; 5) | 1 (-1; 6) | 1 (0; 2) | 0 (0; 1) | 0 (-1; 0) | ns |

| | Probiotic | | | Placebo | | | p-value (between changes) |
|---|---|---|---|---|---|---|---|
| | Baseline | 3 months | Change | Baseline | 3 months | Change | |
| Medicine | 0 (0; 0) | 0 (0; 0) | 0 (-1; 0) | 0 (0; 1) | 0 (0; 0.5) | 0 (0; 0) | ns |
| Muscle pain | 0.5 (0; 1) | 0 (0; 1.5) | 0 (-1; 0) | 0 (0; 1) | 0 (0; 1) | 0 (0; 1) | ns |
| Felt tired | 1 (0; 2) | 0 (0; 1) | -1 (-2; -1) | 0 (0; 0.5) | 0 (0; 1) | 0 (0; 0) | 0.014 |
| Staying in bed | 0 (0; 0) | 0 (0; 0) | 0 (0; 0) | 0 (0; 0) | 0 (0; 0) | 0 (0; 0) | ns |
| Cancelled work | 0 (0; 0) | 0 (0; 0) | 0 (0; 0) | 0 (0; 0) | 0 (0; 0) | 0 (0; 0) | ns |
| Cancelled leisure activities | 0 (0; 1) | 0 (0; 0) | 0 (-1; 0) | 0 (0; 0) | 0 (0; 0) | 0 (0; 0) | ns |
| GIQLI | | | | | | | |
| Symptoms | 65 (46; 66) | 64.5 (55; 68.5) | 2 (-4; 3) | 69 (62; 72.5) | 69 (61; 74) | 1 (-5; 6) | ns |
| Emotions | 12 (11.5; 15.5) | 15 (13.5; 16.5) | 2.5 (0; 4) | 16 (13; 16) | 15 (13; 16) | 0 (0; 1) | ns |
| Physical function | 17 (10.5; 20.5) | 17.5 (14; 21.5) | 4.5 (-4; 6) | 20 (13.5; 23.5) | 21 (17; 24) | 2 (-2; 5) | ns |
| Social function | 12 (8; 14) | 14.5 (10.5; 15) | 1 (0; 3) | 15.5 (13.5; 16) | 14.5 (13; 16) | 0 (-1; 2) | ns |
| Medical treatment | 3 (3; 4) | 4 (4; 4) | 0 (0; 1) | 4 (4; 4) | 4 (3.5; 4) | 0 (0; 0) | ns |
| SF-36 | | | | | | | |
| Physical functioning | 80 (70; 95) | 85 (80; 90) | 5 (-10; 20) | 80 (65; 95) | 90 (80; 100) | 5 (0; 10) | ns |
| Role Physical | 62.5 (25; 87.5) | 50 (50; 75) | 0 (-50; 0) | 100 (50; 100) | 100 (100; 100) | 0 (0; 25) | ns |
| Bodily pain | 73 (51.5; 100) | 74 (61; 100) | 16 (-78; 20) | 100 (62; 100) | 100 (67; 100) | 0 (0; 0) | ns |
| General health | 62 (52; 82) | 67 (62; 87) | 2.5 (-10; 23.5) | 62 (56; 77) | 75 (57; 87) | 5 (-5; 18) | ns |
| Vitality | 45 (35; 60) | 50 (45; 60) | 7.5 (-17.5; 22.5) | 67.5 (47.5; 75) | 60 (40; 72.5) | -5 (-10; 5) | ns |
| Social functioning | 81.2 (62.5; 100) | 87.5 (75; 100) | 0 (-25; 37.5) | 100 (68.8; 100) | 100 (81.2; 100) | 0 (0; 12.5) | ns |
| Role Emotional | 83.3 (16.7; 100) | 66.7 (66.7; 100) | 0 (-33.3; 33.3) | 100 (83.3; 100) | 100 (83.3; 100) | 0 (0; 0) | ns |
| Mental health | 84 (52; 84) | 80 (72; 84) | 2 (-10; 14) | 86 (68; 92) | 86 (76; 96) | 0 (0; 4) | ns |

| | Probiotic | | | Placebo | | | p-value (between changes) |
|---|---|---|---|---|---|---|---|
| | Baseline | 3 months | Change | Baseline | 3 months | Change | |
| COVID recovery | | | | | | | |
| Recovery scale | 4 (3; 4) | 4 (3; 4) | 0 (0; 1) | 4 (3; 5) | 5 (4; 5) | 0.5 (0; 1) | ns |
| mMRC Dyspnoea Scale | 1 (1; 1) | 1 (0; 1) | 0 (-1; 0) | 1 (0; 2) | 0 (0; 1) | 0 (-1; 0) | ns |

Data are shown as median and 95% confidence interval unless stated otherwise; ns not significant

Table S8: Baseline and end of treatment values, and changes in ARTIQ, GIQLI and SF-36 questionnaire, recovery- and mMRC score parameters

| | Probiotic | | | Placebo | | | p-value (between changes) |
|---|---|---|---|---|---|---|---|
| | Baseline | 6 months | Change | Baseline | 6 months | Change | |
| ARTIQ | | | | | | | |
| Physical upper airways | 4 (1.5; 9) | 3 (1; 5) | -0.5 (-6; 0.5) | 1 (1; 3.5) | 3 (1; 7) | 1 (-1; 4) | ns |
| Physical lower airways | 3 (0.5; 4.5) | 1 (0; 2) | -0.5 (-2; 0) | 1.5 (0; 2) | 0 (0; 1.5) | -1 (-2; 0) | ns |
| Psychological | 2 (0; 5) | 0 (0; 1) | -1 (-5; 0) | 0 (0; 1) | 0 (0; 0) | 0 (-1; 0) | ns |
| Sleep | 1 (0; 2) | 1 (1; 3) | 1 (-1; 3) | 1 (0; 2) | 0.5 (0; 1.5) | 0 (-1; 1) | ns |
| Medicine | 0 (0; 0) | 0 (0; 1) | 0 (0; 1) | 0 (0; 1) | 0 (0; 1) | 0 (0; 0) | ns |
| Muscle pain | 0.5 (0; 1) | 1 (0; 1) | 0 (0; 0) | 0 (0; 1) | 0 (0; 1) | 0 (0; 0) | ns |
| Felt tired | 1 (0; 2) | 0 (0; 1) | -1 (-1; 0) | 0 (0; 0.5) | 0 (0; 0.5) | 0 (0; 0) | ns |
| Staying in bed | 0 (0; 0) | 0 (0; 0) | 0 (0; 0) | 0 (0; 0) | 0 (0; 0) | 0 (0; 0) | |
| Cancelled work | 0 (0; 0) | 0 (0; 0) | 0 (0; 0) | 0 (0; 0) | 0 (0; 0) | 0 (0; 0) | ns |
| Cancelled leisure activities | 0 (0; 1) | 0 (0; 0) | 0 (-1; 0) | 0 (0; 0) | 0 (0; 0) | 0 (0; 0) | ns |
| GIQLI | | | | | | | |
| Symptoms | 65 (46; 66) | 63 (51; 69) | 3.5 (-3; 6) | 69 (62; 72.5) | 69 (55; 72.5) | 1 (-3; 6) | ns |
| Emotions | 12 (11.5; 15.5) | 14 (13; 15) | 2 (-6; 3) | 16 (13; 16) | 15 (13.5; 16) | 0 (-1; 1) | ns |
| Physical function | 17 (10.5; 20.5) | 13 (10; 25) | 3 (-8; 7) | 20 (13.5; 23.5) | 20 (15; 24.5) | 3 (-2; 5) | ns |
| Social function | 12 (8; 14) | 10 (9; 15) | 1 (-3; 2.5) | 15.5 (13.5; 16) | 15 (13; 16) | 0 (-1; 2) | ns |

| | Probiotic | | | Placebo | | | p-value (between changes) |
|---|---|---|---|---|---|---|---|
| | Baseline | 6 months | Change | Baseline | 6 months | Change | |
| Medical treatment | 3 (3; 4) | 4 (3; 4) | 0 (-0.5; 0.5) | 4 (4; 4) | 4 (3; 4) | 0 (-1; 0) | ns |
| SF-36 | | | | | | | |
| Physical functioning | 80 (70; 95) | 90 (80; 95) | 10 (0; 15) | 80 (65; 95) | 95 (87.5; 100) | 10 (0; 25) | ns |
| Role Physical | 62.5 (25; 87.5) | 87.5 (50; 100) | 12.5 (0; 25) | 100 (50; 100) | 100 (87.5; 100) | 0 (0; 50) | ns |
| Bodily pain | 73 (51.5; 100) | 74 (52.1; 84) | -5 (-13.5; 16.5) | 100 (62; 100) | 92 (67; 100) | 0 (-19; 0) | ns |
| General health | 62 (52; 82) | 69.5 (42; 82) | 10 (-12; 17) | 62 (56; 77) | 74.5 (71; 88.5) | 5 (-7; 20) | ns |
| Vitality | 45 (35; 60) | 55 (32.5; 70) | 0 (0; 25) | 67.5 (47.5; 75) | 60 (47.5; 77.5) | 0 (-10; 5) | ns |
| Social functioning | 81.2 (62.5; 100) | 93.8 (62.5; 100) | 0 (-6.25; 25) | 100 (68.8; 100) | 100 (75; 100) | 0 (0; 12.5) | ns |
| Role Emotional | 83.3 (16.7; 100) | 83.3 (33.3; 100) | 0 (0; 33.3) | 100 (83.3; 100) | 100 (83.3; 100) | 0 (0; 0) | ns |
| Mental health | 84 (52; 84) | 76 (52; 88) | 4 (-16; 16) | 86 (68; 92) | 86 (74; 92) | 0 (-8; 12) | ns |
| COVID recovery | | | | | | | |
| Recovery scale | 4 (3; 4) | 4.5 (3; 5) | 1 (0; 1) | 4 (3; 5) | 5 (4; 5) | 1 (0; 1) | ns |
| mMRC Dyspnoea Scale | 1 (1; 1) | 0 (0; 1) | -1 (-1; 0) | 1 (0; 2) | 0 (0; 1) | 0 (-1; 0) | ns |

Data are shown as median and 95% confidence interval unless stated otherwise; ns not significant

Table S9: Baseline and after 3 months of treatment values, and changes in standard laboratory parameters; data are given as median (95% confidence interval).

| | Probiotics | | | Placebo | | | p-values (between changes) |
|---|---|---|---|---|---|---|---|
| | Baseline | 3 months | Change | Baseline | 3 months | Change | |
| Erythrocyte (10^12/L) | 4.66 (4.54; 5) | 4.67 (4.53; 4.95) | -0.09 (-0.12; 0.11) | 4.88 (4.59; 5.16) | 4.9 (4.61; 5.18) | 0.06 (0.02; 0.16) | ns |
| Hemoglobin (g/dL) | 14.4 (14; 15.7) | 14.8 (13.9; 15.6) | 0.1 (-0.2; 0.2) | 14.5 (13.6; 15) | 14.7 (13.5; 15.3) | 0.2 (-0.05; 0.5) | ns |
| Hematocrit (%) | 42.2 (41.5; 44.4) | 43.4 (40.2; 43.7) | -0.7 (-1.3; 0.2) | 43 (40.4; 43.7) | 43.3 (41.6; 44.7) | 0.85 (0.5; 1.7) | 0.016 |
| Mean corpuscular volume (fL) | 89.9 (86.6; 93.8) | 90.3 (85.1; 95.3) | -1.5 (-2.1; 1) | 86.6 (83.4; 89) | 86.6 (84.6; 88.9) | 0.5 (-0.2; 1.4) | ns |

| | Probiotics | | | Placebo | | | p-values (between changes) |
|---|---|---|---|---|---|---|---|
| | Baseline | 3 months | Change | Baseline | 3 months | Change | |
| Mean corpuscular hemoglobin (pg) | 31 (30.1; 31.8) | 31 (29.8; 32.7) | -0.3 (-0.7; 0.9) | 30.1 (27.4; 31) | 29.5 (28.1; 30.7) | -0.05 (-0.3; 0.25) | ns |
| Mean corpuscular hemoglobin concentration (g/dL) | 34.2 (33.7; 35.1) | 34.6 (34.3; 35.3) | 0.4 (-0.3; 0.6) | 33.6 (32.8; 35.3) | 33.6 (32.7; 34.7) | 0 (-0.95; 0.4) | ns |
| Leukocyte (10^9/L) | 6.07 (4.6; 6.84) | 6.4 (5.04; 8.24) | 0.24 (0.04; 1.5) | 6.02 (5.28; 6.54) | 6.21 (5.6; 6.99) | 0.285 (-0.57; 0.745) | ns |
| Neutrophils (%) | 60.5 (49; 62) | 56 (48; 66) | -1 (-4; 6) | 61.5 (47.5; 65.5) | 61 (50; 63) | -0.5 (-6.5; 0.5) | ns |
| Eosinophils (%) | 2.5 (2; 3) | 3 (2; 3) | 0 (-1; 1) | 2 (1.5; 5) | 3 (2; 5) | 0 (-0.5; 1) | ns |
| Monocytes (%) | 9 (8; 11) | 9 (7; 10) | 0 (-1; 2) | 8.5 (6; 10) | 9 (8; 10) | 0.5 (0; 1.5) | ns |
| Basophils % | 1 (1; 1) | 1 (1; 1) | 0 (0; 0) | 1 (0; 1) | 1 (0; 1) | 0 (0; 0) | ns |
| Lymphocytes (%) | 29 (27; 35) | 30 (24; 39) | 1 (-5; 4) | 28.5 (23.5; 31.5) | 30 (24; 33) | 0 (-2; 5.5) | ns |
| Thrombocytes (10^9/L) | 232 (198; 282) | 237 (197; 267) | -3 (-30; 36) | 224 (202; 278) | 228 (208; 275) | 0 (-18.5; 18.5) | ns |
| Mean platelet volume (fL) | 10.2 (9.1; 10.8) | 10.3 (9.2; 10.9) | 0.1 (-0.1; 0.5) | 10.5 (9.85; 11.4) | 10.2 (9.8; 11.4) | -0.05 (-0.35; 0.25) | ns |
| Prothrombin time (%) | 112 (106; 116) | 107 (97; 114) | -2 (-10; 0) | 113 (108; 118) | 110 (101; 116) | -5.5 (-8; 2) | ns |
| Prothrombin time international normalized ratio | 0.94 (0.92; 0.97) | 0.96 (0.94; 1) | 0.05 (0.01; 0.06) | 0.935 (0.91; 0.96) | 0.96 (0.94; 0.99) | 0.015 (-0.02; 0.03) | ns |
| Activated partial thromboplastin time (s) | 31 (29.5; 34.5) | 30.5 (28.2; 31.6) | -0.7 (-3.7; 1) | 30.3 (27.3; 34.4) | 29.8 (28.2; 31.3) | 0.3 (-0.4; 2.1) | ns |
| Fibrinogen (mg/dL) | 278 (231; 339) | 270 (216; 348) | 11 (-9; 87) | 294 (258; 323) | 317 (282; 338) | 12 (2.5; 37.5) | ns |
| Triglyceride (mg/dL) | 100 (53; 199) | 148 (129; 163) | 42 (0; 84) | 136 (85.5; 212) | 132 (87; 230) | 18.5 (-4; 44) | ns |
| Cholesterol (mg/dL) | 214 (148; 239) | 200 (137; 229) | -6 (-17; 8) | 186 (160; 209) | 171 (161; 223) | 9.5 (-5.5; 25.5) | ns |
| Glucose (mg/dL) | 102 (93; 124) | 100 (89; 174) | -4 (-11; 12) | 100 (94; 108) | 96 (92; 107) | 4.5 (-3.5; 9.5) | ns |
| Creatinine (mg/dL) | 0.985 (0.71; 1.07) | 1.13 (0.86; 1.22) | 0.07 (-0.01; 0.15) | 0.84 (0.76; 0.98) | 0.88 (0.77; 1) | 0 (-0.03; 0.04) | ns |
| Glomerular filtration rate (ml/min) | 84.3 (74.5; 91.2) | 74.3 (67.2; 82.3) | -11.2 (-13; 0.97) | 91 (84.8; 104) | 93.3 (81.3; 104) | -0.15 (-1.77; 4.45) | ns |

| | Probiotics | | | Placebo | | | p-values (between changes) |
|---|---|---|---|---|---|---|---|
| | Baseline | 3 months | Change | Baseline | 3 months | Change | |
| Sodium (mmol/L) | 142 (139; 142) | 139 (139; 141) | -1 (-2; 0) | 140 (139; 140) | 139 (139; 140) | -1 (-1; 1) | ns |
| Potassium (mmol/L) | 4.2 (3.7; 4.6) | 4.2 (3.7; 4.4) | 0 (-0.3; 0.2) | 4.1 (3.9; 4.2) | 4 (3.9; 4.3) | 0 (-0.2; 0.2) | ns |
| Chloride (mmol/l) | 103 (99; 106) | 102 (100; 104) | 0 (-3; 1) | 103 (102; 104) | 102 (101; 103) | -2 (-2; 0) | ns |
| Calcium (mmol/L) | 2.38 (2.35; 2.39) | 2.39 (2.36; 2.46) | 0.04 (-0.03; 0.08) | 2.37 (2.32; 2.41) | 2.37 (2.35; 2.4) | 0 (-0.02; 0.03) | ns |
| Total protein (g/dL) | 7.5 (7.2; 7.8) | 7.7 (7.2; 7.8) | -0.05 (-0.1; 0.3) | 7.2 (6.9; 7.3) | 7.2 (7.1; 7.4) | 0.05 (-0.1; 0.3) | ns |
| Albumin (g/dL) | 4.6 (4.4; 4.7) | 4.7 (4.5; 4.8) | 0.1 (-0.1; 0.2) | 4.5 (4.4; 4.7) | 4.4 (4.4; 4.6) | -0.1 (-0.3; 0.2) | ns |
| Bilirubin direct (mg/dL) | 0.18 (0.15; 0.24) | 0.21 (0.14; 0.25) | -0.03 (-0.09; 0.09) | 0.21 (0.13; 0.28) | 0.225 (0.18; 0.245) | -0.03 (-0.07; 0.05) | ns |
| Bilirubin total (mg/dL) | 0.455 (0.38; 0.61) | 0.53 (0.36; 0.62) | -0.12 (-0.22; 0.16) | 0.56 (0.41; 0.69) | 0.55 (0.45; 0.61) | -0.05 (-0.14; 0.15) | ns |
| Alkaline Phosphatase (U/L) | 60 (44; 85) | 69 (40; 79) | 8 (-4; 12) | 64 (56; 77.5) | 65 (60; 77) | 2 (-1; 8) | ns |
| Aspartate aminotransferase (U/L) | 24 (19; 34) | 37 (24; 47) | 8 (-1; 17) | 19 (16; 23) | 22 (16; 27) | 2 (-2; 4) | ns |
| Alanine aminotransferase (U/L) | 33.5 (22; 56) | 48 (42; 87) | 10 (-2; 22) | 17 (16; 23) | 19 (16; 27) | 2 (1; 6) | ns |
| Gamma glutamyltransferase (U/l) | 27 (19; 83) | 64 (27; 104) | 10 (3; 24) | 26.5 (15; 30.5) | 31 (16; 34) | 2 (-1; 5) | ns |
| Cholinesterase (U/L) | 8670 (8540; 9240) | 9120 (8750; 9460) | 485 (-216; 1150) | 8190 (7080; 8790) | 8790 (7450; 9460) | 51.5 (-73; 651) | ns |
| C-reactive protein (mg/L) | 1.65 (0.7; 3.1) | 1.9 (1.4; 2.2) | 0.5 (-0.4; 1.2) | 1.7 (0.9; 4.3) | 2.6 (0.9; 6.1) | 0 (-0.2; 1.1) | ns |
| Lactate dehydrogenase (U/L) | 187 (174; 219) | 245 (163; 267) | 39.5 (-13.5; 69.5) | 187 (176; 208) | 176 (168; 191) | 0 (-22; 13) | ns |

Data are shown as median and 95% confidence interval unless stated otherwise; ns not significant

Table S10: Baseline and end of treatment values, and changes in standard laboratory parameters; data are given as median (95% confidence interval)

| | Probiotic | | | Placebo | | | p-value (between changes) |
|---|---|---|---|---|---|---|---|
| | Baseline | 6 months | Change | Baseline | 6 months | Change | |
| Erythrocyte (10^12/L) | 4.66 (4.54; 4.88) | 4.62 (4.4; 5.05) | 0.025 (-0.23; 0.1) | 4.88 (4.59; 5.16) | 4.82 (4.74; 5.17) | -0.01 (-0.29; 0.17) | ns |
| Hemoglobin (g/dL) | 14.4 (14; 15.7) | 14.6 (13.7; 15.6) | -0.1 (-0.8; 0.7) | 14.5 (13.6; 15) | 14.7 (13.5; 15) | 0 (-0.4; 0.5) | ns |
| Hematocrit (%) | 42.2 (41.5; 44.4) | 41.4 (39.5; 44.4) | -0.75 (-2.4; 0.6) | 43 (40.4; 43.7) | 42.7 (40.4; 45.4) | 0.2 (-1.75; 2.2) | ns |
| Mean corpuscular volume (fL) | 89.9 (86.6; 93.8) | 88.9 (85.6; 95.4) | -0.9 (-1; -0.1) | 86.6 (83.4; 89) | 86.8 (84.2; 90.1) | 0.75 (-0.55; 2.2) | ns |
| Mean corpuscular hemoglobin (pg) | 31 (30.1; 31.8) | 31.1 (30.3; 32.8) | 0.15 (-0.5; 1) | 30.1 (27.4; 31) | 28.9 (27.5; 31) | 0.1 (-0.5; 0.45) | ns |
| Mean corpuscular hemoglobin concentration (g/dL) | 34.2 (33.7; 35.6) | 35 (34; 35.5) | 0.45 (-0.4; 1.1) | 33.6 (32.8; 35.3) | 33.6 (32.7; 34.6) | -0.55 (-0.8; 0.4) | 0.037 |
| Leukocyte (10^9/L) | 6.07 (4.6; 6.84) | 6.2 (4.33; 7.15) | 0.305 (-0.62; 1.15) | 6.02 (5.28; 6.54) | 6.57 (6.35; 7.36) | 1.12 (-0.175; 1.44) | ns |
| Neutrophils (%) | 60.5 (49; 62) | 58 (47; 66) | 0.5 (-4; 5) | 61.5 (47.5; 65.5) | 61 (56; 64) | -1.5 (-5; 3.5) | ns |
| Eosinophils (%) | 2.5 (2; 3) | 3 (2; 4) | 0 (0; 1) | 2 (1.5; 5) | 2 (2; 4) | 0 (0; 1) | ns |
| Monocytes (%) | 9 (8; 11) | 9 (6; 10) | 0 (-2; 1) | 8.5 (6; 10) | 9 (7; 11) | 1 (-1.5; 2.5) | ns |
| Basophils % | 1 (1; 1) | 1 (1; 1) | 0 (-1; 0) | 1 (0; 1) | 1 (1; 1) | 0 (0; 0.5) | ns |
| Lymphocytes (%) | 29 (27; 35) | 31 (22; 38.5) | -0.5 (-4; 4) | 28.5 (23.5; 31.5) | 27 (23; 30) | 0.5 (-6.5; 3) | ns |
| Thrombocytes (10^9/L) | 232 (198; 282) | 232 (201; 261) | -2.5 (-28; 35) | 224 (202; 278) | 218 (207; 283) | -0.5 (-19.5; 15.5) | ns |
| Mean platelet volume (fL) | 10.2 (9.1; 10.8) | 10.3 (9.4; 11.1) | 0.3 (-0.15; 0.5) | 10.5 (9.85; 11.4) | 10.4 (9.7; 11.3) | -0.1 (-0.3; 0.1) | ns |
| Prothrombin time (%) | 112 (106; 116) | 118 (104; 120) | 2 (-8; 11) | 113 (108; 118) | 113 (108; 120) | 1 (-4; 8) | ns |
| Prothrombin time international normalized ratio | 0.94 (0.92; 0.97) | 0.92 (0.89; 0.99) | 0 (-0.05; 0.04) | 0.935 (0.91; 0.96) | 0.93 (0.91; 0.96) | 0.01 (-0.05; 0.02) | ns |
| Activated partial thromboplastin time (s) | 31 (29.5; 34.5) | 32.4 (27.2; 33.6) | -1.6 (-2.2; -0.5) | 30.3 (27.3; 34.4) | 30 (28.7; 33.8) | 1.2 (0.3; 4.5) | ns |
| Fibrinogen (mg/dL) | 278 (231; 339) | 273 (220; 373) | -6 (-52.5; 22.5) | 294 (258; 323) | 294 (268; 348) | 2 (-34; 22) | ns |
| Triglyceride (mg/dL) | 100 (53; 199) | 150 (93; 257) | 37 (22; 65) | 136 (85.5; 212) | 112 (60; 146) | -25 (-58.5; 66) | ns |

| | Probiotic | | | Placebo | | | p-value (between changes) |
|---|---|---|---|---|---|---|---|
| | Baseline | 6 months | Change | Baseline | 6 months | Change | |
| Cholesterol (mg/dL) | 214 (148; 239) | 222 (153; 287) | 6.5 (-1; 48) | 186 (160; 209) | 168 (162; 214) | 2.5 (-1.5; 11.5) | ns |
| Glucose (mg/dL) | 102 (93; 124) | 102 (89; 161) | -1 (-7; 37) | 100 (94; 108) | 99 (96; 103) | 1 (-4.5; 9) | ns |
| Creatinine (mg/dL) | 0.985 (0.71; 1.07) | 0.98 (0.8; 1.18) | 0.015 (-0.03; 0.15) | 0.84 (0.76; 0.98) | 0.9 (0.85; 0.99) | 0.05 (0; 0.08) | ns |
| Glomerular filtration rate (ml/min) | 84.3 (74.5; 91.2) | 82.5 (66.4; 93.8) | -1.87 (-14; 2.66) | 91 (84.8; 104) | 88.2 (82.2; 98.2) | -2.69 (-7.48; -0.35) | ns |
| Sodium (mmol/L) | 142 (138; 142) | 140 (139; 141) | -0.5 (-2; 1) | 140 (139; 140) | 140 (139; 141) | 1 (0; 2) | ns |
| Potassium (mmol/L) | 4.2 (3.7; 4.6) | 4.05 (3.7; 4.5) | 0 (-0.3; 0.2) | 4.1 (3.9; 4.2) | 4.2 (4; 4.4) | 0.2 (0.1; 0.3) | 0.049 |
| Chloride (mmol/l) | 103 (99; 106) | 102 (99; 105) | -0.5 (-2; 1) | 103 (102; 104) | 103 (102; 104) | 0 (-1; 2) | ns |
| Calcium (mmol/L) | 2.38 (2.35; 2.39) | 2.41 (2.35; 2.5) | 0.03 (-0.02; 0.12) | 2.37 (2.32; 2.41) | 2.37 (2.36; 2.42) | 0 (-0.03; 0.08) | ns |
| Total protein (g/dL) | 7.5 (7.2; 7.8) | 7.45 (6.8; 7.8) | 0 (-0.3; 0.4) | 7.2 (6.9; 7.3) | 7.3 (7.1; 7.5) | 0.25 (-0.1; 0.5) | ns |
| Albumin (g/dL) | 4.6 (4.4; 4.7) | 4.55 (4.3; 4.9) | 0.05 (-0.2; 0.2) | 4.5 (4.4; 4.7) | 4.5 (4.4; 4.6) | 0 (-0.1; 0.1) | ns |
| Bilirubin direct (mg/dL) | 0.18 (0.15; 0.24) | 0.19 (0.11; 0.35) | 0.01 (-0.03; 0.04) | 0.21 (0.13; 0.28) | 0.195 (0.155; 0.285) | 0.01 (-0.03; 0.04) | ns |
| Bilirubin total (mg/dL) | 0.455 (0.38; 0.61) | 0.445 (0.28; 0.55) | -0.06 (-0.23; 0.08) | 0.56 (0.41; 0.69) | 0.45 (0.31; 0.61) | -0.04 (-0.14; 0.03) | ns |
| Alkaline Phosphatase (U/L) | 60 (44; 85) | 72.5 (40; 83) | 2 (-5; 10) | 64 (56; 77.5) | 63 (58; 72) | 1.5 (-3; 8.5) | ns |
| Aspartate aminotransferase (U/L) | 24 (19; 34) | 28.5 (19; 34) | 0.5 (-3; 9) | 19 (16; 23) | 21 (19; 23) | 3 (1; 5) | ns |
| Alanine aminotransferase (U/L) | 33.5 (22; 56) | 33.5 (21; 47) | -1.5 (-3; 1) | 17 (16; 23) | 18 (16; 29) | 1 (-2; 4) | ns |
| Gamma glutamyltransferase (U/l) | 27 (19; 83) | 42 (20; 97) | 8.5 (3; 33) | 26.5 (15; 30.5) | 36 (17; 37) | 2 (-0.5; 7.5) | ns |
| Cholinesterase (U/L) | 8670 (8540; 9240) | 8720 (8250; 9400) | 200 (-282; 1200) | 8190 (7080; 8790) | 8710 (7600; 9410) | 318 (-372; 704) | ns |
| C-reactive protein (mg/L) | 1.65 (0.7; 3.1) | 1.7 (0.7; 3.8) | 0 (-0.8; 0.7) | 1.7 (0.9; 4.3) | 2.2 (0.8; 5.9) | 0.1 (-0.8; 0.6) | ns |

| | Probiotic | | | Placebo | | | p-value (between changes) |
|---|---|---|---|---|---|---|---|
| | Baseline | 6 months | Change | Baseline | 6 months | Change | |
| Lactate dehydrogenase (U/L) | 187 (174; 219) | 196 (187; 232) | 2 (-17; 29) | 187 (176; 208) | 180 (168; 194) | 1 (-22; 19.5) | ns |

Data are shown as median and 95% confidence interval unless stated otherwise; ns not significant

Table S11: Baseline and after 3 months of treatment values, and changes in lung function parameters; data are given as median (95% confidence interval).

| | Probiotics | | | Placebo | | | p-values (between changes) |
|---|---|---|---|---|---|---|---|
| | Baseline | 3 months | Change | Baseline | 3 months | Change | |
| 6 min walking test (6MWT) | | | | | | | |
| Borg dyspnea score before 6MWT | 0 (0; 0.5) | 0 (0; 0) | 0 (0; 0) | 0 (0; 0.5) | 0 (0; 0) | 0 (0; 0) | ns |
| Borg dyspea score after 6MWT | 0.75 (0; 1) | 0 (0; 1) | 0 (-0.5; 0) | 0.5 (0; 3) | 0 (0; 0) | -0.25 (-2; 0) | ns |
| Oxygen saturation before | 95.5 (94; 97) | 96 (93; 97) | 0 (-1; 1) | 96 (95; 98) | 97 (95; 98) | 0 (-1; 1) | ns |
| Oxygen saturation after | 96 (87; 96) | 96 (93; 98) | 2 (0; 3) | 95 (91; 95) | 94 (93; 97) | 1 (-1; 3) | Ns |
| Distance (m) | 423 (403; 465) | 462 (438; 468) | 6 (-22; 31) | 420 (388; 528) | 454 (418; 480) | 12.5 (-3; 51) | ns |
| Spirometry | | | | | | | |
| FVC (L) | 4.2 (2.77; 4.51) | 4.12 (4.1; 4.6) | -0.08 (-0.1; 0.12) | 4.19 (3.59; 4.76) | 4.19 (3.64; 4.59) | 0 (-0.07; 0.05) | ns |
| FVC (% predicted) | 89.5 (77; 107) | 87 (79; 104) | -1 (-3; 2) | 96 (93; 109) | 98 (91; 101) | 0 (-2; 2) | ns |
| (z-score) | -0.745 (-1.61; 0.53) | -1.04 (-1.44; 0.33) | -0.06 (-0.2; 0.17) | -0.31 (-0.53; 0.54) | 0.02 (-0.54; 0.83) | 0.005 (-0.1; 0.07) | ns |
| $FEV_1$ (L) | 3.42 (2.71; 3.69) | 3.44 (3.13; 3.82) | 0.05 (-0.05; 0.18) | 3.29 (2.7; 3.54) | 3.19 (2.69; 3.64) | 0.02 (-0.03; 0.15) | ns |
| FEV1 (% predicted) | 94.5 (83; 101) | 97 (86; 103) | 2 (-1; 6) | 90 (88; 96) | 93 (88; 101) | 1 (-1; 5) | ns |
| (z-score) | -0.375 (-1.12; 0.12) | -0.28 (-0.92; 0.18) | 0.15 (-0.1; 0.38) | -0.75 (-0.95; -0.3) | -0.485 (-0.81; 0.05) | 0.095 (-0.23; 0.3) | ns |
| PEF | 9.01 (6.05; 12) | 10.1 (8.33; 12.6) | 0.52 (-0.39; 0.94) | 9.1 (7.56; 9.78) | 9.07 (6.64; 9.75) | -0.27 (-0.74; 0.22) | ns |

| | Probiotics | | | Placebo | | | p-values (between changes) |
|---|---|---|---|---|---|---|---|
| | Baseline | 3 months | Change | Baseline | 3 months | Change | |
| (% predicted) | 115 (94; 127) | 120 (98; 132) | 6 (-6; 13) | 110 (105; 118) | 107 (98; 114) | -3.5 (-10; 1) | ns |
| (z-score) | 1.12 (-0.39; 2.17) | 1.51 (-0.11; 2.59) | 0.42 (-0.41; 0.82) | 0.71 (0.35; 1.17) | 0.515 (-0.1; 0.97) | -0.24 (-0.65; 0.11) | ns |
| Body plethysmography | | | | | | | |
| VCmax | 4.45 (2.95; 4.69) | 4.58 (4.12; 4.79) | -0.09 (-0.16; 0.12) | 4.55 (3.73; 4.97) | 4.6 (3.75; 4.82) | -0.02 (-0.14; 0.05) | ns |
| VC (% predicted) | 92.5 (89; 108) | 91 (87; 104) | -2 (-3; 3) | 104 (98; 112) | 106 (100; 112) | -0.5 (-3; 1) | ns |
| (z-score) | -0.51 (-0.8; 0.64) | -0.63 (-1.02; 0.33) | -0.13 (-0.22; 0.22) | 0.26 (-0.16; 0.94) | 0.38 (-0.01; 1.03) | -0.025 (-0.19; 0.09) | ns |
| RV | 1.92 (1.53; 2.26) | 2.01 (1.83; 2.15) | -0.04 (-0.11; 0.21) | 2.18 (1.75; 2.52) | 2.2 (1.71; 2.48) | -0.01 (-0.08; 0.23) | ns |
| RV (% predicted) | 91.5 (77; 104) | 91 (81; 114) | -2 (-5; 11) | 98 (81; 108) | 89.5 (77; 108) | -2.5 (-6; 5) | ns |
| (z-score) | -0.51 (-1.14; 0.2) | -0.42 (-1.2; 0.63) | -0.1 (-0.33; 0.55) | -0.49 (-1.1; 0.48) | -0.585 (-1.06; 0.36) | -0.035 (-0.26; 0.56) | ns |
| TLC | 6.44 (4.84; 6.86) | 6.77 (5.95; 6.84) | 0.1 (-0.21; 0.13) | 6.47 (5.76; 7.54) | 6.51 (5.69; 7.09) | -0.04 (-0.13; 0.05) | ns |
| TLC (% predicted) | 91.5 (86; 115) | 90 (85; 114) | 2 (-3; 2) | 105 (93; 110) | 97 (91; 110) | -1 (-2; 1) | ns |
| (z-score) | -0.915 (-1.55; 0.57) | -1.1 (-1.67; 1.2) | 0.15 (-0.29; 0.19) | 0.52 (-0.73; 0.88) | -0.235 (-1.09; 0.9) | -0.065 (-0.18; 0.09) | ns |
| RV to TLC | 31.2 (26.3; 35.1) | 30.8 (27.1; 32.2) | -0.32 (-1.05; 2.78) | 31.2 (28.6; 34.1) | 30.1 (27.6; 34.4) | 0.37 (-1.15; 2.85) | ns |
| (% predicted) | 87 (80; 98) | 93 (80; 95) | -1 (-3; 7) | 83 (78; 91) | 86.5 (81; 90) | 3 (-3; 9.62) | ns |
| (z-score) | -0.86 (-1.17; -0.17) | -0.46 (-1.36; -0.29) | -0.05 (-0.19; 0.42) | -0.95 (-1.68; -0.43) | -0.69 (-1.94; -0.46) | 0.07 (-0.21; 0.45) | ns |
| $R_{total}$ | 0.195 (0.14; 0.27) | 0.18 (0.15; 0.23) | 0.01 (-0.03; 0.03) | 0.17 (0.13; 0.32) | 0.19 (0.15; 0.21) | 0.005 (-0.03; 0.06) | ns |
| (% predicted) | 64.5 (47; 89) | 60 (49; 78) | 2 (-9; 13) | 56 (45; 107) | 63 (50; 70) | 1 (-18; 18) | ns |

| | Probiotics | | | Placebo | | | p-values (between changes) |
|---|---|---|---|---|---|---|---|
| | Baseline | 3 months | Change | Baseline | 3 months | Change | |
| $DL_{CO}$ | 8.01 (6.21; 10.4) | 8.6 (5.78; 10.3) | 0.02 (-0.698; 1) | 6.65 (6.19; 8.72) | 6.3 (5.62; 8.06) | -0.36 (-0.58; 0.27) | ns |
| (% predicted) | 87 (80; 102) | 87 (75; 106) | 1 (-5.36; 11) | 88 (70; 95) | 77 (62; 99) | -6 (-6; 3) | ns |
| (z-score) | -0.81 (-1.42; 0.14) | -0.83 (-1.88; 0.39) | 0.04 (-0.46; 0.59) | -0.8 (-2.08; -0.36) | -1.37 (-2.69; -0.17) | -0.36 (-0.48; 0.2) | ns |
| $V_A$ | 6.01 (5.33; 6.45) | 5.83 (5.37; 6.52) | -0.055 (-0.195; 0.124) | 6.16 (5.24; 6.35) | 5.93 (4.8; 6.36) | 0.01 (-0.22; 0.21) | ns |
| (% predicted) | 90 (83; 101) | 88 (81; 101) | -1 (-3; 1.5) | 100 (91; 108) | 100 (90; 111) | 0 (-4; 5) | ns |
| (z-score) | -0.81 (-1.41; 0.06) | -0.97 (-1.68; 0.12) | -0.05 (-0.239; 0.141) | 0.02 (-0.74; 0.63) | 0.03 (-0.85; 0.88) | 0.01 (-0.36; 0.37) | ns |
| $DL_{CO}$ to $V_A$ | 1.47 (1.27; 1.61) | 1.42 (1.02; 1.75) | 0.02 (-0.0557; 0.145) | 1.07 (1.02; 1.48) | 1.01 (0.95; 1.63) | -0.04 (-0.07; 0.04) | ns |
| (% predicted) | 100 (92; 114) | 103 (71; 113) | 1.5 (-4; 10) | 76 (73; 95) | 76 (69; 106) | -2 (-4; 3) | ns |
| (z-score) | 0.02 (-0.5; 0.87) | 0.21 (-1.98; 0.96) | 0.095 (-0.25; 0.65) | -1.48 (-1.85; -0.38) | -1.46 (-2.24; 0.42) | -0.18 (-0.32; 0.19) | ns |
| $V_C$ | 56.4 (45.3; 71.5) | 53.4 (38.5; 62) | -1.3 (-10.6; 3.3) | 46.4 (41.7; 66.1) | 44.4 (38.9; 52.2) | 0.9 (-4.3; 5) | ns |
| (% predicted) | 68.5 (61; 79) | 62 (51; 72) | -2 (-12; 1.5) | 62 (50; 78) | 59 (46; 70) | 2 (-5; 6) | ns |
| $DL_{NO}$ | 35.2 (31.2; 40.7) | 32.6 (27.3; 43.4) | -1.05 (-3.01; 3.29) | 29 (24.6; 35.9) | 28.6 (24.6; 34.5) | 1.98 (-0.94; 2.65) | ns |
| $DL_{NO}$ to $DL_{CO}$ | 4.39 (4.14; 4.64) | 4.72 (3.8; 5.17) | 0.135 (-0.79; 0.67) | 4.3 (3.99; 4.56) | 4.45 (4.3; 5.11) | 0.58 (0; 0.84) | ns |
| $DL_{NO}$ to $V_A$ | 6.83 (5.76; 7.06) | 6.1 (5.31; 7.73) | 0.265 (0.24; 1.29) | 5.76 (4.48; 6.92) | 5.37 (4.49; 6.97) | 0.2 (-0.15; 0.43) | ns |

FVC - forced vital capacity; FEV1 - forced expiratory volume (first second); PEF - peak expiratory flow; VCmax - maximum vital capacity; RV - residual volume; TLC - total lung capacity; Rtot - Resistance; DLCO - Diffusion capacity for carbonmonoxide; VA - alveolar volume; VC - alveolar membrane; DLNO - Diffusion capacity for nitric oxid; Data are shown as median and 95% confidence interval unless stated otherwise; ns not significant

Table S12: Baseline and end of treatment values, and changes in lung function parameters; data are given as median (95% confidence interval).

| | Probiotics | | | Placebo | | | p-values (between changes) |
|---|---|---|---|---|---|---|---|
| | Baseline | 6 months | Change | Baseline | 6 months | Change | |
| 6 min walking test | | | | | | | |
| Borg rating before | 0 (0; 0.5) | 0 (0; 0) | 0 (-0.5; 0) | 0 (0; 0.5) | 0 (0; 0) | 0 (-0.5; 0) | ns |
| Borg rating after | 0.75 (0; 1) | 0 (0; 0.5) | 0 (-0.5; 0) | 0.5 (0; 3) | 0 (0; 0) | -0.25 (-3; 0) | ns |
| Oxygen saturation before | 95.5 (94; 97) | 97 (95; 98) | 1 (-1; 2) | 96 (95; 98) | 96 (95; 97) | -1 (-1; 1) | ns |
| Oxygen saturation after | 96 (89; 96) | 95.5 (94; 97) | 0.5 (0; 3) | 95 (91; 95) | 95.5 (93; 97) | 1 (0; 2) | ns |
| Distance (m) | 423 (403; 465) | 476 (408; 486) | 16.5 (-12.5; 66) | 420 (388; 528) | 483 (436; 513) | 21.5 (-27; 72) | ns |
| Spirometry | | | | | | | |
| FVC (L) | 4.2 (2.77; 4.51) | 4.18 (3.38; 4.5) | -0.01 (-0.14; 0.13) | 4.19 (3.59; 4.76) | 4.41 (3.78; 4.8) | 0.03 (-0.05; 0.23) | ns |
| (% predicted) | 89.5 (77; 107) | 87.5 (80; 108) | 0 (-3; 3) | 96 (93; 109) | 103 (92; 113) | 1 (0; 6) | ns |
| (z-score) | -0.745 (-1.61; 0.53) | -0.895 (-1.41; 0.65) | 0.03 (-0.23; 0.23) | -0.31 (-0.53; 0.54) | 0.21 (-0.56; 0.92) | 0.07 (-0.04; 0.38) | ns |
| $FEV_1$ | 3.42 (2.38; 3.69) | 3.47 (2.55; 3.73) | 0.045 (-0.05; 0.17) | 3.29 (2.7; 3.54) | 3.46 (2.62; 3.82) | 0.03 (-0.06; 0.16) | ns |
| (% predicted) | 94.5 (83; 101) | 96.5 (84; 101) | 2.5 (-1; 6) | 90 (88; 96) | 95 (89; 100) | 1 (-1; 6) | ns |
| (z-score) | -0.375 (-1.12; 0.12) | -0.225 (-1.01; 0.09) | 0.225 (-0.1; 0.38) | -0.75 (-0.95; -0.3) | -0.38 (-0.84; -0.02) | 0.11 (-0.06; 0.42) | ns |
| PEF | 9.01 (6.05; 12) | 9.8 (5.98; 11.7) | -0.045 (-0.55; 0.93) | 9.1 (7.56; 9.78) | 9.01 (6.66; 10.1) | -0.14 (-0.62; 0.69) | ns |
| (% predicted) | 115 (94; 127) | 112 (100; 131) | 0 (-6.5; 13) | 110 (105; 118) | 107 (90; 118) | -1 (-7; 8) | ns |
| (z-score) | 1.12 (-0.39; 2.17) | 0.94 (0.01; 2.24) | 0.01 (-0.587; 0.82) | 0.71 (0.35; 1.17) | 0.51 (-0.78; 1.33) | -0.09 (-0.51; 0.62) | ns |
| Body plethysmography | | | | | | | |
| VCmax | 4.45 (2.95; 4.69) | 4.52 (2.88; 4.77) | 0.06 (-0.07; 0.1) | 4.55 (3.73; 4.97) | 4.74 (3.78; 5.19) | 0.03 (-0.23; 0.04) | ns |

| | Probiotics | | | Placebo | | | p-values (between changes) |
|---|---|---|---|---|---|---|---|
| | Baseline | 6 months | Change | Baseline | 6 months | Change | |
| (% predicted) | 92.5 (89; 108) | 91.5 (85; 110) | 2 (1; 3) | 104 (98; 112) | 107 (98; 117) | 1 (-5; 2) | ns |
| (z-score) | -0.51 (-0.8; 0.64) | -0.585 (-0.96; 0.753) | 0.125 (0.07; 0.23) | 0.26 (-0.16; 0.94) | 0.51 (-0.16; 1.09) | 0.07 (-0.31; 0.0939) | ns |
| RV | 1.92 (1.53; 2.26) | 1.88 (1.32; 2.35) | 0.035 (-0.19; 0.26) | 2.18 (1.75; 2.52) | 2.17 (1.78; 2.52) | 0.05 (-0.16; 0.49) | ns |
| (% predicted) | 91.5 (77; 104) | 93 (60; 110) | 1.5 (-8; 10) | 98 (81; 108) | 101 (78; 118) | 1 (-10; 22) | ns |
| (z-score) | -0.51 (-1.14; 0.2) | -0.405 (-1.46; 0.5) | 0.055 (-0.52; 0.59) | -0.49 (-1.1; 0.48) | 0.02 (-1.17; 1.06) | 0.11 (-0.43; 1.15) | ns |
| TLC | 6.44 (4.84; 6.86) | 6.34 (4.47; 7.09) | 0.05 (-0.29; 0.3) | 6.47 (5.76; 7.54) | 6.66 (6.01; 7.99) | 0 (-0.16; 0.52) | ns |
| (% predicted) | 91.5 (86; 108) | 90 (84; 118) | 1.5 (-4; 4) | 105 (93; 110) | 104 (92; 115) | 1 (-2; 7) | ns |
| (z-score) | -0.915 (-1.55; 1.35) | -0.95 (-1.7; 1.54) | 0.115 (-0.409; 0.43) | 0.52 (-0.73; 0.88) | 0.33 (-0.84; 1.3) | 0.12 (-0.22; 0.42) | ns |
| RV to TLC | 31.2 (26.3; 35.1) | 31.9 (30; 35.3) | 1.12 (-2.15; 2.18) | 31.2 (28.6; 34.1) | 31.5 (27.7; 36.2) | 1.98 (-2.04; 3.74) | ns |
| (% predicted) | 87 (80; 97) | 88 (82; 94) | 2 (-7; 5) | 83 (78; 91) | 88 (72; 99) | 5 (-5; 16) | ns |
| (z-score) | -0.86 (-1.17; -0.17) | -0.77 (-1.23; -0.31) | 0.17 (-0.46; 0.34) | -0.95 (-1.68; -0.43) | -0.79 (-1.47; -0.09) | 0.34 (-0.37; 0.61) | ns |
| $R_{total}$ | 0.195 (0.14; 0.27) | 0.21 (0.2; 0.31) | 0.04 (-0.01; 0.07) | 0.17 (0.13; 0.32) | 0.16 (0.15; 0.2) | 0.03 (-0.02; 0.07) | ns |
| (% predicted) | 64.5 (47; 87.5) | 71 (66; 104) | 14 (-3; 23) | 56 (45; 107) | 55 (51; 83) | 9 (-5; 20) | ns |
| $DL_{CO}$ | 8.01 (6.21; 10.4) | 8.18 (5.17; 10.9) | -0.16 (-0.5; 0.88) | 6.65 (6.19; 8.72) | 6.18 (5.84; 7.56) | -0.25 (-0.54; 0.15) | ns |
| (% predicted) | 87 (80; 102) | 85.5 (76; 103) | 1 (-4; 9) | 88 (70; 95) | 81 (70; 94.9) | -3 (-4.5; 2) | ns |
| (z-score) | -0.81 (-1.42; 0.14) | -0.91 (-1.72; 0.19) | 0.04 (-0.3; 0.62) | -0.8 (-2.08; -0.36) | -1.73 (-2.56; -0.44) | -0.13 (-0.31; 0.19) | ns |
| $V_A$ | 6.01 (5.03; 6.45) | 5.93 (3.6; 6.39) | -0.08 (-0.38; 0.14) | 6.16 (5.24; 6.35) | 5.82 (4.49; 7.69) | 0.03 (-0.49; 0.126) | ns |
| (% predicted) | 90 (83; 101) | 86.5 (75; 102) | 0 (-6; 2) | 100 (91; 108) | 96 (91; 108) | 0 (-7; 4) | ns |

| | Probiotics | | | Placebo | | | p-values (between changes) |
|---|---|---|---|---|---|---|---|
| | Baseline | 6 months | Change | Baseline | 6 months | Change | |
| (z-score) | -0.81 (-1.41; 0.06) | -1.16 (-2.15; 0.2) | 0.06 (-0.53; 0.18) | 0.02 (-0.74; 0.63) | -0.12 (-0.73; 0.75) | 0.04 (-0.16; 0.28) | ns |
| $DL_{CO}$ to $V_A$ | 1.47 (1.27; 1.61) | 1.44 (1.11; 1.7) | -0.01 (-0.04; 0.14) | 1.07 (1.02; 1.48) | 1.05 (0.98; 1.41) | -0.02 (-0.04; 0.03) | ns |
| (% predicted) | 100 (92; 114) | 102 (77; 109) | -1 (-3; 10) | 76 (73; 95) | 75 (70; 85) | -1 (-3; 2) | ns |
| (z-score) | 0.02 (-0.5; 0.87) | 0.12 (-1.53; 0.66) | -0.06 (-0.17; 0.65) | -1.48 (-1.85; -0.38) | -1.53 (-2.07; -1.04) | -0.07 (-0.18; 0.15) | ns |
| $V_C$ | 56.4 (44.5; 71.5) | 51 (37.4; 67.8) | -0.25 (-3.75; 2.2) | 46.4 (41.7; 66.1) | 48.8 (38.3; 63.3) | 0.9 (-4.9; 6.1) | ns |
| (% predicted) | 68.5 (63; 79) | 64 (56; 75) | 1 (-4; 4.5) | 62 (50; 78) | 62 (49; 76) | 1 (-5; 9) | ns |
| $DL_{NO}$ | 35.2 (28; 40) | 32 (23.5; 40.8) | -2.96 (-4.47; 1.12) | 29 (24.6; 35.9) | 26.9 (22.9; 38.7) | -0.35 (-2.67; 1.48) | ns |
| $DL_{NO}$ to $DL_{CO}$ | 4.39 (4.2; 4.58) | 4.59 (3.52; 4.83) | -0.22 (-0.8; 0.6) | 4.3 (3.99; 4.56) | 4.23 (3.98; 4.55) | 0.05 (-0.33; 0.23) | ns |
| $DL_{NO}$ to $V_A$ | 6.83 (5.83; 7.08) | 6.09 (5.1; 7.86) | -0.35 (-0.64; 0.58) | 5.76 (4.48; 6.92) | 5.79 (4.39; 6.79) | -0.02 (-0.76; 0.2) | ns |

FVC - forced vital capacity; FEV1 - forced expiratory volume (first second); PEF - peak expiratory flow; VCmax - maximum vital capacity; RV - residual volume; TLC - total lung capacity; Rtot - Resistance; DLCO - Diffusion capacity for carbonmonoxide; VA - alveolar volume; VC - alveolar membrane; DLNO - Diffusion capacity for nitric oxid; Data are shown as median and 95% confidence interval unless stated otherwise; ns not significant

Table S13: Baseline and 3 months of treatment values, and changes in gut permeability and bacterial translocation parameters; data are given as median (95% confidence interval).

| | Probiotic | | | Placebo | | | p-value (between changes) |
|---|---|---|---|---|---|---|---|
| | Baseline | 3 months | Change | Baseline | 3 months | Change | |
| Serum zonulin (ng/mL) | 29.3 (19.7; 74.1) | 24.1 (18.4; 83.2) | 0.952 (-8.8; 10.7) | 38.2 (28.6; 45.4) | 35.9 (32.1; 44.2) | 0.938 (-9.78; 5.67) | ns |
| Diamine Oxidase (U/ml) | 13.2 (10.9; 17.9) | 10.5 (8.1; 19.9) | -1.52 (-3.08; 4.69) | 11.9 (9.57; 15.5) | 12.7 (9.69; 14.6) | -0.401 (-0.89; 0.645) | ns |
| LPS-binding protein (ng/ml) | 15.9 (11; 16.1) | 14.5 (8.95; 21.1) | 0.129 (-2.83; 5.66) | 11.4 (6.94; 15.7) | 12.2 (8.33; 14.4) | 1.39 (0.425; 2.48) | ns |

| | Probiotic | | | Placebo | | | p-value (between changes) |
|---|---|---|---|---|---|---|---|
| | Baseline | 3 months | Change | Baseline | 3 months | Change | |
| sCD14 ( g/mL) | 1.4 (1.16; 1.59) | 1.24 (1.15; 1.61) | -0.0479 (-0.159; 0.172) | 1.26 (1.2; 1.43) | 1.26 (1.22; 1.44) | -0.00474 (-0.0988; 0.0982) | ns |

Table S14: Baseline and end of treatment values, and changes in gut permeability and bacterial translocation parameters; data are given as median (95% confidence interval).

| | Probiotic | | | Placebo | | | p-value (between changes) |
|---|---|---|---|---|---|---|---|
| | Baseline | 6 months | Change | Baseline | 6 months | Change | |
| Serum zonulin (ng/ml) | 29.3 (19.7; 74.1) | 31 (23.1; 86.2) | 7.58 (-3.62; 38.8) | 38.2 (28.6; 45.4) | 38.6 (31.6; 43.9) | 2.57 (-8.44; 6.64) | ns |
| Diamine Oxidase (U/ml) | 13.2 (10.9; 17.9) | 12.5 (8.35; 16.1) | -1.92 (-3.22; 2.14) | 11.9 (9.57; 15.5) | 12.5 (9.94; 13.8) | -0.616 (-1.03; 0.289) | ns |
| LPS-binding protein (ng/ml) | 15.9 (11; 16.1) | 12.3 (11.3; 20.8) | -0.244 (-2.55; 3.03) | 11.4 (6.94; 15.7) | 10.8 (9.35; 16.8) | 0.566 (-0.477; 3.45) | ns |
| sCD14 ( g/mL) | 1.4 (1.16; 1.59) | 1.38 (1.23; 1.6) | 0.0533 (-0.0537; 0.0841) | 1.26 (1.2; 1.43) | 1.27 (1.21; 1.33) | -0.029 (-0.082; 0.0404) | ns |

Data are shown as median and 95% confidence interval unless stated otherwise; ns not significant

Table S15: Baseline and 3 months of treatment values, and changes in macrophage activation markers; data are given as median (95% confidence interval)

| | Probiotic | | | Placebo | | | p-value (between the changes |
|---|---|---|---|---|---|---|---|
| | Baseline | 3 months | Change | Baseline | 3 months | Change | |
| sCD163 (mg/l) | 2.82 (2.06; 3.49) | 2.17 (1.27; 3.64) | -0.26 (-3.06; 0.75) | 1.6 (1.46; 1.74) | 1.7 (1.43; 1.99) | 0 (-0.12; 0.16) | ns |
| sMR (mg/l) | 0.286 (0.194; 0.338) | 0.229 (0.151; 0.372) | -0.007 (-0.116; 0.049) | 0.234 (0.195; 0.247) | 0.217 (0.198; 0.244) | -0.002 (-0.006; 0.034) | ns |

Data are shown as median and 95% confidence interval unless stated otherwise; ns not significant

Table S16: Baseline and end of treatment values, and changes in macrophage activation markers; data are given as median (95% confidence interval)

| | Probiotic | | | Placebo | | | p-value (between changes) |
|---|---|---|---|---|---|---|---|
| | Baseline | 6 months | Change | Baseline | 6 months | Change | |
| sCD163 (mg/l) | 2.82 (2.06; 3.49) | 1.99 (1.56; 3.39) | -0.305 (-0.5; 0.11) | 1.6 (1.46; 1.74) | 1.7 (1.5; 2.09) | 0 (-0.2; 0.14) | ns |
| sMR (mg/l) | 0.286 (0.194; 0.34) | 0.262 (0.182; 0.316) | -0.0025 (-0.025; 0.023) | 0.234 (0.195; 0.247) | 0.232 (0.194; 0.248) | 0.001 (-0.01; 0.018) | ns |

Data are shown as median and 95% confidence interval unless stated otherwise; ns not significant

Table S17: Baseline and 3 months of treatment values, and changes in neutrophil function parameters; data are given as median (95% confidence interval).

| | Probiotic | | | Placebo | | | p-value (between changes) |
|---|---|---|---|---|---|---|---|
| | Baseline | 3 months | Change | Baseline | 3 months | Change | |
| Chemotaxis directedness | 0.434 (0.339; 0.458) | 0.403 (0.322; 0.467) | 0.01 (-0.042; 0.064) | 0.403 (0.364; 0.462) | 0.383 (0.364; 0.406) | -0.027 (-0.131; 0.027) | ns |
| Chemotactic Efficiency Index (CEI) | 0.015 (-0.004; 0.101) | 0.016 (-0.008; 0.112) | -0.012 (-0.109; 0.116) | -0.008 (-0.032; 0.019) | 0.0075 (-0.017; 0.055) | 0.046 (-0.05; 0.073) | ns |
| Phosphate-buffered saline (PBS) | 11900 (5680; 20300) | 10600 (1770; 23300) | -4690 (-23800; 11700) | 6080 (2410; 19200) | 3790 (608; 20600) | -4930 (-18300; 7870) | ns |
| N-Formylmethionyl-leucyl-phenylalanine (fMLF) | 40900 (4980; 81100) | 17100 (12300; 19300) | -46600 (-66000; 4330) | 23800 (12000; 36000) | 17400 (9420; 28700) | 785 (-23700; 15500) | 0.033 |
| Escherichia coli | 60400 (26100; 106000) | 34900 (23900; 48000) | -32800 (-102000; -11200) | 50600 (44500; 63600) | 51700 (43600; 54400) | 5610 (-5000; 18600) | 0.01 |
| Phorbol myristate acetate (PMA) | 216000 (96200; 298000) | 135000 (103000; 151000) | -135000 (-191000; -18600) | 177000 (156000; 187000) | 137000 (104000; 182000) | -26700 (-57900; 28300) | 0.034 |

Data are shown as median and 95% confidence interval unless stated otherwise; ns not significant

Table S18: Baseline and end of treatment values, and changes in neutrophil chemotaxis parameters; data are given as median (95% confidence interval)

| | Probiotic | | | Placebo | | | p-value (between the changes |
|---|---|---|---|---|---|---|---|
| | Baseline | 6 months | Change | Baseline | 6 months | Change | |

| | | | | | | | |
|---|---|---|---|---|---|---|---|
| Directedness | 0.434 (0.339; 0.458) | 0.35 (0.155; 0.495) | -0.093 (-0.351; 0.17) | 0.403 (0.364; 0.462) | 0.42 (0.399; 0.467) | -0.0165 (-0.0615; 0.0575) | ns |
| Chemotactic Efficiency Index (CEI) | 0.015 (-0.004; 0.101) | -0.0045 (-0.0265; 0.102) | -0.01 (-0.124; 0.137) | -0.008 (-0.032; 0.019) | 0.021 (-0.039; 0.049) | 0.013 (-0.0405; 0.0365) | ns |

Data are shown as median and 95% confidence interval unless stated otherwise; ns not significant

Table S19: B- and T-cell populations at baseline and after 3 months of intervention for patients in the probiotic group and the placebo group. Data are given as median (95% confidence interval)

| | Probiotic | | | Placebo | | | p-value (between changes) |
|---|---|---|---|---|---|---|---|
| | Baseline | 3 months | Change | Baseline | 3 months | Change | |
| % CD3+CD8+ T cells in lymphocytes | 18 (14.8; 23.7) | 19.5 (14.6; 27) | 2.2 (-0.684; 10.2) | 20.5 (13.7; 25.8) | 21.2 (17.5; 25.3) | 0.6 (-0.5; 3.8) | ns |
| % CD161 in CD3+CD8 T cells | 7.6 (3.82; 11.1) | 8.37 (4.2; 12.1) | -1.36 (-5.28; 1.88) | 8.68 (6.78; 12.8) | 6.28 (5.15; 7.97) | -3.07 (-5.17; -1.17) | ns |
| % CD3+CD4+ T cells in lymphocytes | 48.4 (38.6; 64) | 44.8 (39.2; 51.3) | 0.4 (-6.8; 2) | 48.5 (40.6; 55) | 46.4 (43.8; 51.6) | -0.2 (-1.9; 1.76) | ns |
| % CD45RA-Ki67+ in CD3+CD4+ T cells | 1.62 (0.8; 2.09) | 1.57 (1.1; 2.18) | -0.005 (-1.09; 0.82) | 1.86 (1.03; 1.98) | 1.46 (1.26; 2.45) | 0.2 (-0.72; 0.4) | ns |
| % CD45RA+Ki67- in CD3+CD4+ T cells | 43.3 (25.6; 54.9) | 34.2 (25.8; 48.4) | -0.51 (-7.4; 0.2) | 45.3 (39.2; 57) | 45.3 (35.7; 57.3) | 0.5 (-1.9; 4.4) | ns |
| % CD45RA-CD39+ in CD3+CD4+ T cells | 4.93 (3.02; 6.77) | 3.01 (0.925; 6.25) | -1.38 (-2.24; -0.97) | 4.76 (1.76; 6.43) | 3.26 (0.74; 4.3) | -0.905 (-2.55; -0.39) | ns |
| % CD45RA+CD39- in CD3+CD4+ T cells | 42.4 (23.2; 55) | 31.8 (28.3; 48.2) | -1 (-5.5; 3.6) | 43.6 (37.8; 55.8) | 47.2 (39.1; 63) | 1.95 (-1.2; 3.2) | ns |
| % CD25+FoxP3+ Tregs in CD3+CD4+ T cells | 4.27 (2.99; 5.55) | 4.27 (3.64; 4.91) | -0.19 (-0.837; 1.13) | 4.14 (3.25; 5.81) | 3.86 (3.71; 5.12) | -0.13 (-0.64; 0.53) | ns |
| % CD147high in Tregs | 10 (6.71; 15.5) | 9.8 (7.46; 10.3) | -1.55 (-12.1; 2.05) | 6.92 (6.34; 10.1) | 7.84 (5.69; 9) | 0.805 (-1.95; 2.68) | ns |
| % effector Tregs in Tregs | 25.2 (19.4; 32.6) | 25.3 (14.2; 36.7) | -6.5 (-15.3; 9.5) | 18.1 (16.1; 29.9) | 23.2 (15.8; 35.3) | 5.15 (1.3; 8.4) | ns |
| % naive Tregs in Tregs | 33.8 (13.8; 44.4) | 23.6 (21.2; 37.8) | -6.64 (-14.4; 11.5) | 41.8 (33; 53.6) | 37.3 (22.8; 53.4) | -5.4 (-10.2; 5.6) | ns |
| % CM cells in CD4+ T cells | 11.9 (7.81; 18.6) | 12.9 (10.6; 17.9) | 3.65 (-7.8; 8.37) | 11.4 (8.63; 13.8) | 11.8 (7.47; 13.6) | -1.74 (-2.8; 1.4) | ns |
| % EM cells in CD4+ T cells | 41.3 (34.1; 67.3) | 45.2 (32.4; 57.1) | -1.8 (-12.7; 6.9) | 40.2 (28.8; 45.6) | 35.1 (22.5; 43.5) | -0.2 (-6.3; 5.16) | ns |

| | Probiotic | | | Placebo | | | p-value (between changes) |
|---|---|---|---|---|---|---|---|
| | Baseline | 3 months | Change | Baseline | 3 months | Change | |
| % NAIVE cells in CD4+ T cells | 22.8 (6.88; 34) | 19.6 (8.88; 40.1) | 5.42 (0.89; 8.6) | 21.2 (18.3; 41.2) | 33.8 (19.5; 41.8) | 1.1 (-3.2; 4.7) | ns |
| % TEMRA cells in CD4+ T cells | 21.2 (11.4; 23.7) | 17.2 (7.71; 22.5) | -3.7 (-10.6; 0.47) | 16.9 (14.2; 24.8) | 20.9 (17.5; 26.2) | 2.1 (0.6; 4.1) | 0.022 |
| % CM cells in CD8+ T cells | 4.2 (2.59; 7.84) | 2.95 (1.54; 5.43) | -2.74 (-3.74; -0.21) | 3.61 (1.77; 10) | 3.37 (1.74; 5.73) | -1.05 (-3.69; 0.16) | ns |
| % EM cells in CD8+ T cells | 24.6 (14.7; 43.2) | 25.7 (18.6; 38.1) | -4.5 (-6.1; 1.53) | 24.6 (20.1; 34.4) | 32.5 (16.4; 33.9) | 1.9 (-3.7; 8.8) | ns |
| % NAIVE cells in CD8+ T cells | 24.2 (11; 41.5) | 12.6 (7.06; 31.4) | 0.29 (-14.4; 3.13) | 16.4 (10.7; 39.9) | 10.8 (9.53; 38.1) | 0.1 (-3.4; 0.8) | ns |
| % TEMRA cells in CD8+ T cells | 37.4 (24.1; 62.4) | 55.5 (32.4; 71.1) | 5.55 (-0.05; 17.9) | 37.1 (25.4; 52.9) | 43.7 (28.2; 53.2) | -1 (-3.3; 12.8) | ns |
| % CD19+CD20+ B cells in lymphocytes | 8.36 (5.97; 12) | 9.64 (6.95; 12.6) | 1.83 (-1.85; 2.48) | 7.93 (5.72; 10.7) | 7.88 (6.38; 10.6) | 0.5 (-1.13; 0.93) | ns |
| % activated B cells in B cells | 1.36 (1.06; 2.31) | 1.26 (1.06; 1.5) | -0.615 (-1.24; 0.32) | 1.7 (1.41; 1.99) | 1.4 (1.14; 1.79) | -0.21 (-0.54; 0.15) | ns |
| % CCR7+ B cells in B cells | 13.7 (5.51; 25.7) | 4.72 (3.18; 6.5) | -10.5 (-22.5; -4.89) | 7.3 (5; 13.6) | 4.39 (3.73; 5.03) | -2.31 (-9.21; -1.86) | ns |
| % naive B cells in B cells | 72.6 (62.5; 79.6) | 74.8 (64.2; 84.4) | 2.7 (-2.9; 6.35) | 72.3 (61.5; 79.6) | 74.2 (59; 80.7) | -0.9 (-1.5; 0.9) | ns |
| % marginal Zone B cells in B cells | 6.91 (4.59; 14.5) | 7.33 (2.45; 13.4) | -0.67 (-2.73; 1.94) | 4.08 (3.38; 7.61) | 4.1 (3.46; 7.82) | 0.21 (-0.5; 1.07) | ns |
| % Non-switched memory B cells in B cells | 13.9 (8.49; 18) | 15 (10.9; 20.9) | -1.23 (-4.3; 5.66) | 10.5 (8.18; 12.3) | 11.1 (8.62; 14.6) | 2.3 (0.65; 4.6) | ns |
| % Class-switched memory B cells in B cells | 12 (7.02; 17.1) | 11.6 (6.82; 13.9) | -1.39 (-3.4; 0.7) | 13.7 (6.96; 18.6) | 13.6 (6.99; 17.7) | 0.6 (0.15; 0.74) | ns |
| % IgD post-switched B cells in B cells | 12 (7.48; 15.2) | 12.6 (7.85; 15.8) | 1.1 (-2.05; 3.22) | 11 (10.1; 16.7) | 11.6 (9.99; 18.2) | 1.01 (-0.4; 1.5) | ns |
| % IgM only memory B cells in B cells | 7.06 (3.69; 8.59) | 5.76 (2.51; 7.6) | -0.31 (-2.03; 2) | 4.81 (2.31; 6.48) | 2.88 (1.88; 5.27) | -0.3 (-1.98; -0.11) | ns |

Data are shown as median and 95% confidence interval unless stated otherwise; ns not significant

Table S20: B- and T-cell populations at baseline and after 6 months of intervention for patients in the probiotic group and the placebo group. Data are given as median (95% confidence interval)

| | Probiotic | | | Placebo | | | p-value (between changes) |
|---|---|---|---|---|---|---|---|
| | Baseline | 6 months | Change | Baseline | 6 months | Change | |
| % CD3+CD8+ T cells in lymphocytes | 18 (14.8; 23.7) | 18.8 (15.2; 22.2) | 1.6 (-0.35; 6.6) | 20.5 (13.7; 25.8) | 19 (16.2; 21.6) | 0.4 (-4.1; 2.5) | ns |
| % CD161 in CD3+CD8 T cells | 7.6 (3.82; 11.1) | 6.5 (4.15; 10.4) | -2.61 (-3.44; -0.87) | 8.68 (6.78; 12.8) | 6.82 (3.89; 9.91) | -1.24 (-5.49; 1.51) | ns |
| % CD3+CD4+ T cells in lymphocytes | 48.4 (37.6; 64) | 48.7 (39.9; 61.9) | -0.1 (-4.8; 5) | 48.5 (40.6; 55) | 52.6 (46.4; 55.2) | 2.55 (-4.7; 8.5) | ns |
| % CD45RA-Ki67+ in CD3+CD4+ T cells | 1.62 (0.8; 2.09) | 1.09 (0.79; 1.65) | -0.54 (-1; -0.01) | 1.86 (1.03; 1.98) | 1.23 (0.97; 1.73) | -0.13 (-0.78; 0.82) | ns |
| % CD45RA+Ki67- in CD3+CD4+ T cells | 43.3 (25.6; 54.9) | 55.1 (39.3; 57.7) | 6.2 (1.5; 8.1) | 45.3 (39.2; 57) | 48.4 (39.8; 65.5) | 3 (0; 8.9) | ns |
| % CD45RA-CD39+ in CD3+CD4+ T cells | 4.93 (3.02; 6.77) | 3.55 (2.51; 5.25) | -0.86 (-1.52; 0.76) | 4.76 (1.76; 6.43) | 2.9 (1.15; 4.17) | -0.965 (-2.26; -0.12) | ns |
| % CD45RA+CD39- in CD3+CD4+ T cells | 42.4 (23.2; 55) | 53.5 (35.3; 55.7) | 4.6 (-1.5; 7.7) | 43.6 (37.8; 55.8) | 48.2 (41.1; 64.9) | 3.15 (1.2; 9.1) | ns |
| % CD25+FoxP3+ Tregs in CD3+CD4+ T cells | 4.27 (2.51; 5.57) | 6.08 (5.01; 6.29) | 2.32 (0.66; 3.61) | 4.14 (3.25; 5.81) | 5.68 (4.26; 7.07) | 1.21 (0.14; 1.44) | ns |
| % CD147high in Tregs | 10 (6.71; 15.5) | 7.35 (6.89; 7.68) | -4.23 (-8.39; -2.06) | 6.92 (6.34; 10.1) | 7.82 (6.2; 9.81) | 0.28 (-1.56; 2.68) | ns |
| % effector Tregs in Tregs | 25.2 (19.4; 33.3) | 24 (18.6; 25.3) | -1.1 (-4.2; 3.8) | 18.1 (16.1; 29.9) | 16.6 (15.3; 24.2) | -1.3 (-8; 2.7) | ns |
| % naive Tregs in Tregs | 33.8 (13.8; 44.4) | 35.8 (31.6; 42.2) | -4.8 (-6.1; 19.3) | 41.8 (33; 53.6) | 41 (24.8; 53.7) | 0.45 (-10.3; 6.6) | ns |
| % CM cells in CD4+ T cells | 11.9 (7.81; 18.5) | 7.8 (6.38; 10.2) | -2.35 (-8.4; -1.43) | 11.4 (8.63; 13.8) | 7.32 (5.86; 9) | -3.98 (-7.46; -0.39) | ns |
| % EM cells in CD4+ T cells | 41.3 (34.1; 67.3) | 35.3 (30.3; 62.6) | -3.6 (-6.9; 5.3) | 40.2 (28.8; 45.6) | 31.3 (24.7; 46.6) | 1.3 (-6.3; 5.3) | ns |

| | Probiotic | | | Placebo | | | p-value (between changes) |
|---|---|---|---|---|---|---|---|
| | Baseline | 6 months | Change | Baseline | 6 months | Change | |
| % NAIVE cells in CD4+ T cells | 22.8 (6.88; 34) | 26.7 (4.86; 37.1) | 8.88e-16 (-3.04; 9.2) | 21.2 (18.3; 41.2) | 26.3 (15; 42.6) | -2.2 (-6.8; 0.1) | ns |
| % TEMRA cells in CD4+ T cells | 21.2 (11.4; 23.7) | 25.8 (17.5; 27) | 4.48 (1.9; 9.8) | 16.9 (14.2; 24.8) | 25.7 (19.9; 31.7) | 11.4 (0.2; 14.6) | ns |
| % CM cells in CD8+ T cells | 4.2 (2.59; 7.84) | 1.85 (1.54; 2.67) | -2.78 (-4.56; -0.77) | 3.61 (1.77; 10) | 2.91 (1.5; 4.44) | -2.06 (-6.82; 0.06) | ns |
| % EM cells in CD8+ T cells | 24.6 (14.7; 43.2) | 20.5 (16.4; 36.4) | -3.15 (-6.8; 0.6) | 24.6 (20.1; 34.4) | 23.2 (20.8; 36.7) | 0.7 (-1.7; 4.8) | ns |
| % NAIVE cells in CD8+ T cells | 24.2 (10.4; 41.5) | 14.8 (4.67; 38.6) | -4.55 (-14.4; -1.6) | 16.4 (10.7; 39.9) | 11 (10.6; 37.5) | -2.4 (-7; 0.2) | ns |
| % TEMRA cells in CD8+ T cells | 37.4 (24.1; 60.8) | 54.6 (31.7; 72.7) | 12.6 (3.3; 18.7) | 37.1 (25.4; 52.9) | 43.5 (35.7; 64.9) | 5.5 (-0.1; 14.2) | ns |
| % CD19+CD20+ B cells in lymphocytes | 8.36 (5.97; 12) | 9.24 (6.89; 10.7) | 0.88 (-0.53; 3.4) | 7.93 (5.72; 10.7) | 9.17 (8.49; 11.9) | 1.35 (-0.5; 2.25) | ns |
| % activated B cells in B cells | 1.36 (1.06; 2.31) | 1.23 (0.81; 1.65) | -0.37 (-0.66; 0.14) | 1.7 (1.41; 1.99) | 1.51 (1.05; 1.62) | -0.14 (-0.72; 0.16) | ns |
| % CCR7+ B cells in B cells | 13.7 (5.51; 25.7) | 1.7 (1.3; 3.74) | -9.47 (-22; -4.35) | 7.3 (5; 13.6) | 1.71 (1.03; 4.71) | -4.89 (-11.5; -2.86) | ns |
| % naive B cells in B cells | 72.6 (62.5; 79.6) | 68.5 (61.1; 81.9) | -1.45 (-3.1; 7.3) | 72.3 (61.5; 79.6) | 72.1 (64.6; 79.9) | -1.1 (-2.4; 2.2) | ns |
| % marginal Zone B cells in B cells | 6.91 (4.59; 14.5) | 11.1 (4.05; 12.2) | 1.57 (-4.71; 6.15) | 4.08 (3.38; 7.61) | 4.09 (2.78; 8.71) | -0.06 (-1.3; 0.46) | ns |
| % Non-switched memory B cells in B cells | 13.9 (8.49; 18) | 13 (11.7; 14.8) | 2.2 (-6.7; 5.33) | 10.5 (8.18; 12.3) | 6.72 (5.45; 9.09) | -1.44 (-5.25; -1.15) | ns |
| % Class-switched memory B cells in B cells | 12 (7.02; 16.2) | 10.2 (6.22; 15.7) | -1.05 (-5.11; 2.01) | 13.7 (6.96; 18.6) | 12.5 (7.64; 18.6) | 0.68 (-1.2; 3.2) | ns |

| | Probiotic | | | Placebo | | | p-value (between changes) |
|---|---|---|---|---|---|---|---|
| | Baseline | 6 months | Change | Baseline | 6 months | Change | |
| % IgD post-switched B cells in B cells | 12 (7.77; 15.2) | 11.2 (6.52; 16.7) | -1.33 (-3.9; 2.38) | 11 (10.1; 16.7) | 13.4 (11; 17.9) | 1.96 (0; 3.3) | ns |
| % IgM only memory B cells in B cells | 7.06 (3.69; 8.59) | 4.04 (2.87; 6.01) | -1.54 (-5.01; 0.3) | 4.81 (2.31; 6.48) | 3.67 (2.12; 4.73) | 0.01 (-1.14; 0.57) | ns |

Data are shown as median and 95% confidence interval unless stated otherwise; ns not significant

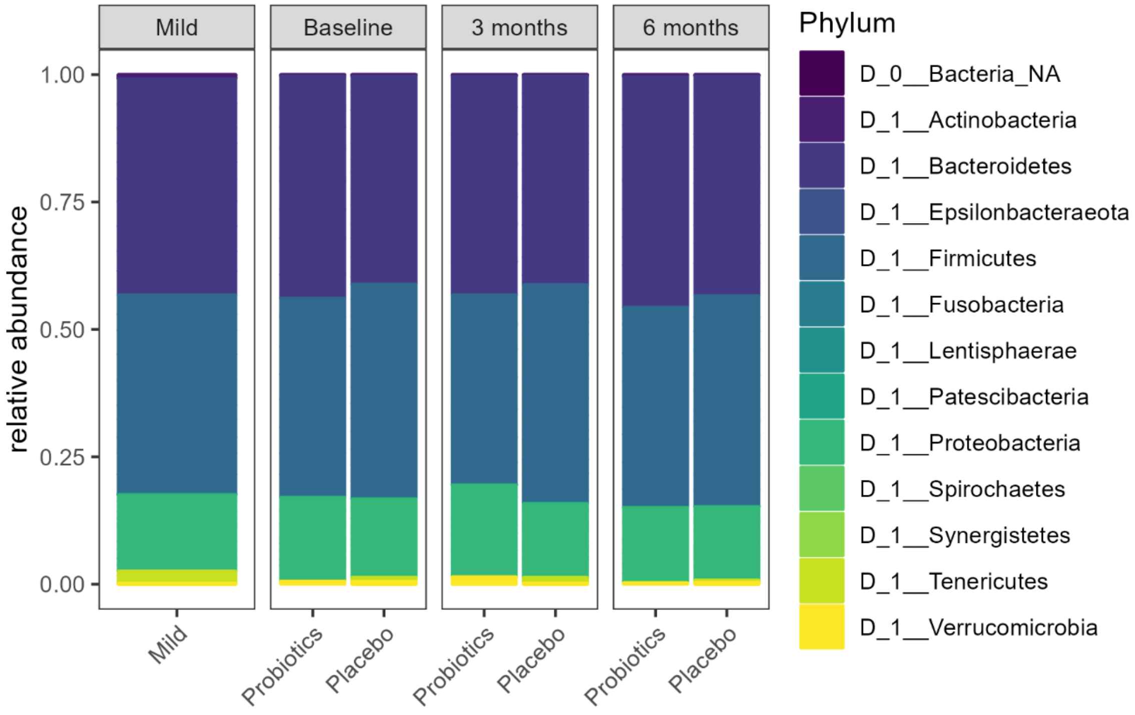


Figure S1: Taxa plot on Phylum level for all groups and time points.

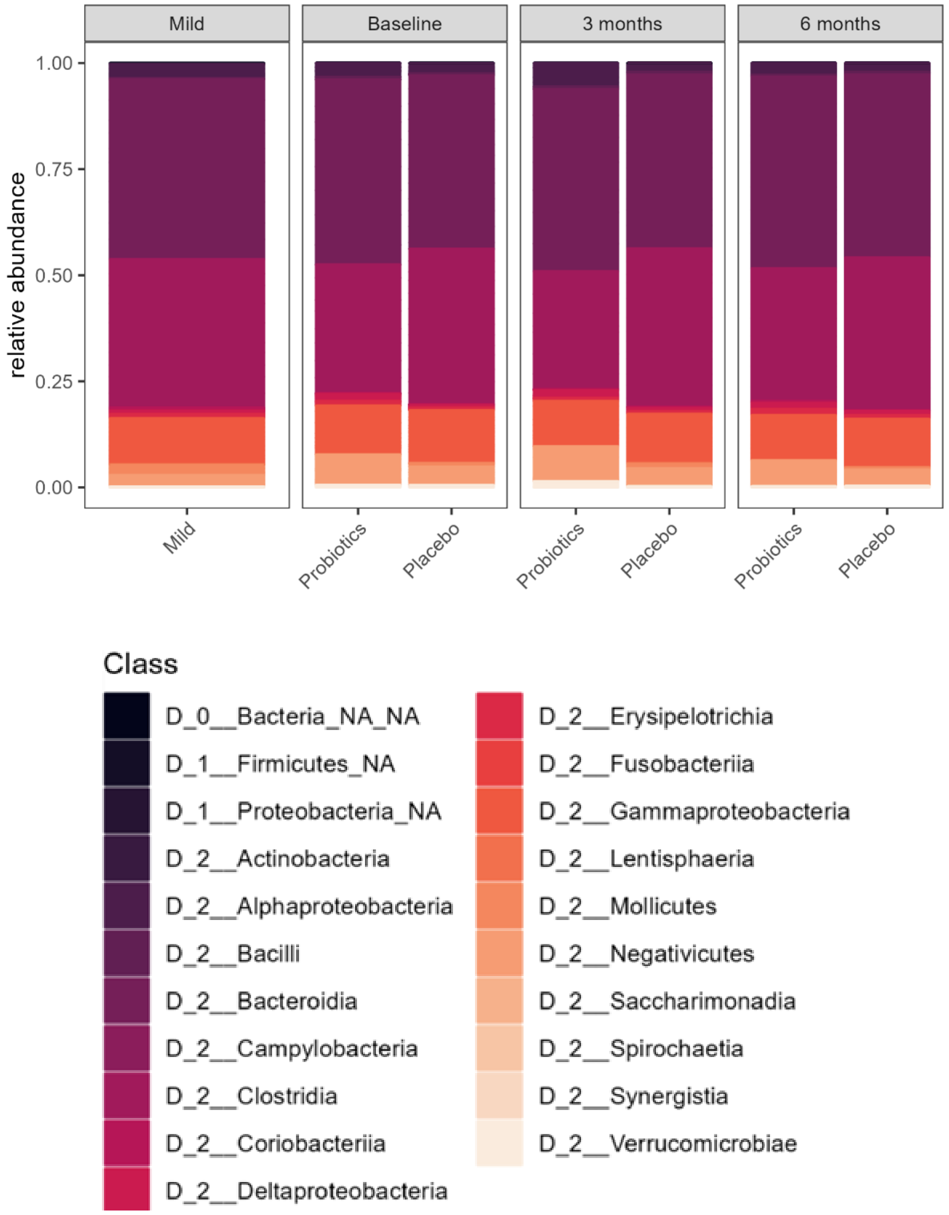


Figure S2: Taxa plot on Class level for all groups and time points.

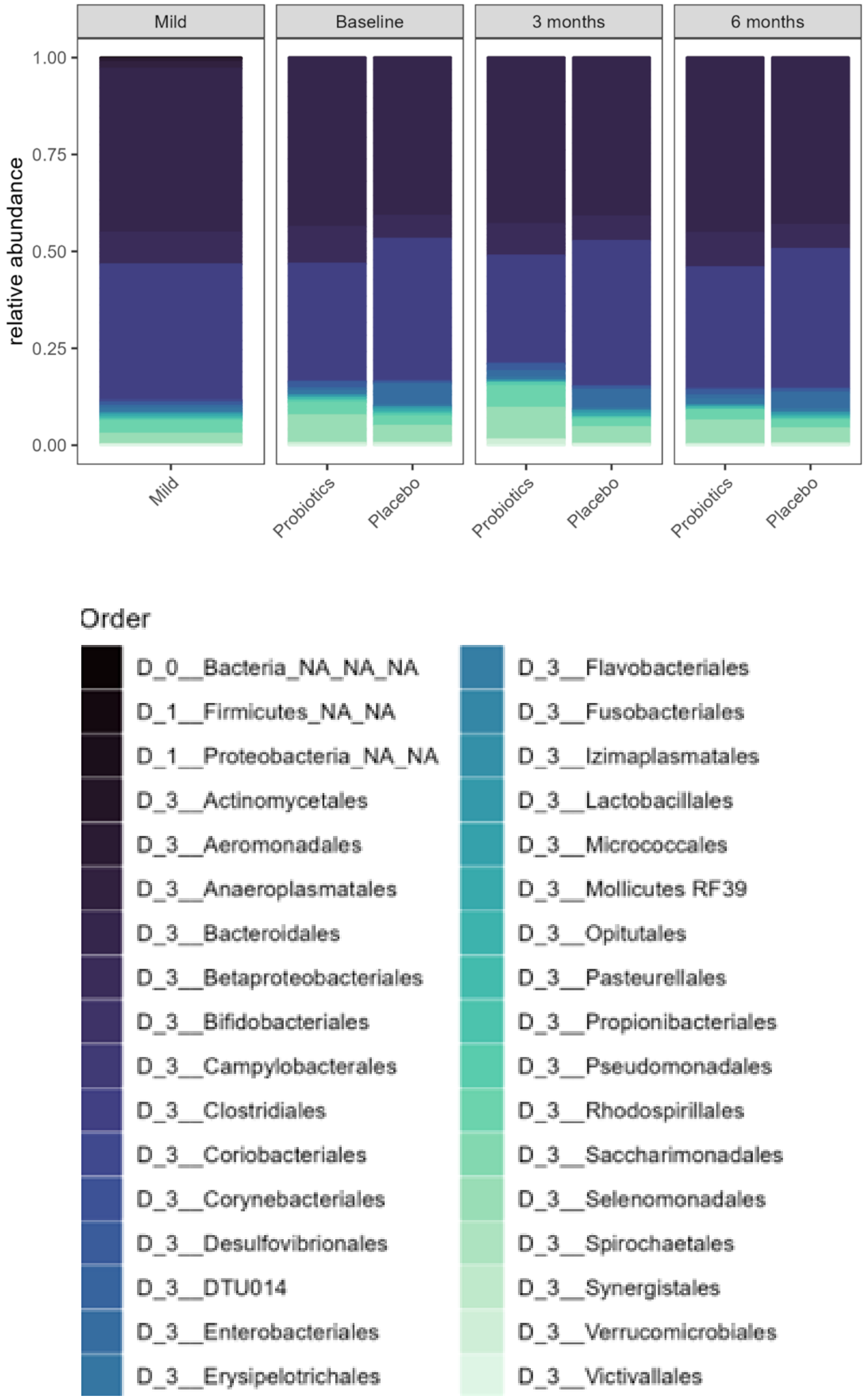


Figure S3: Taxa plot on Order level for all groups and time points.

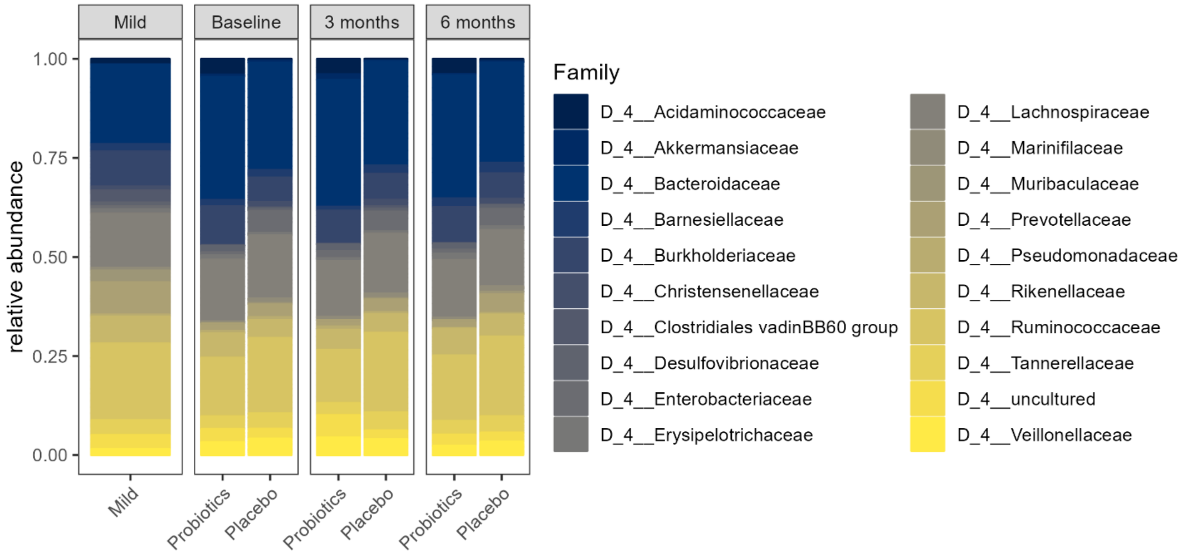


Figure S4: Taxa plot of the 20 most abundant families for all groups and time points.

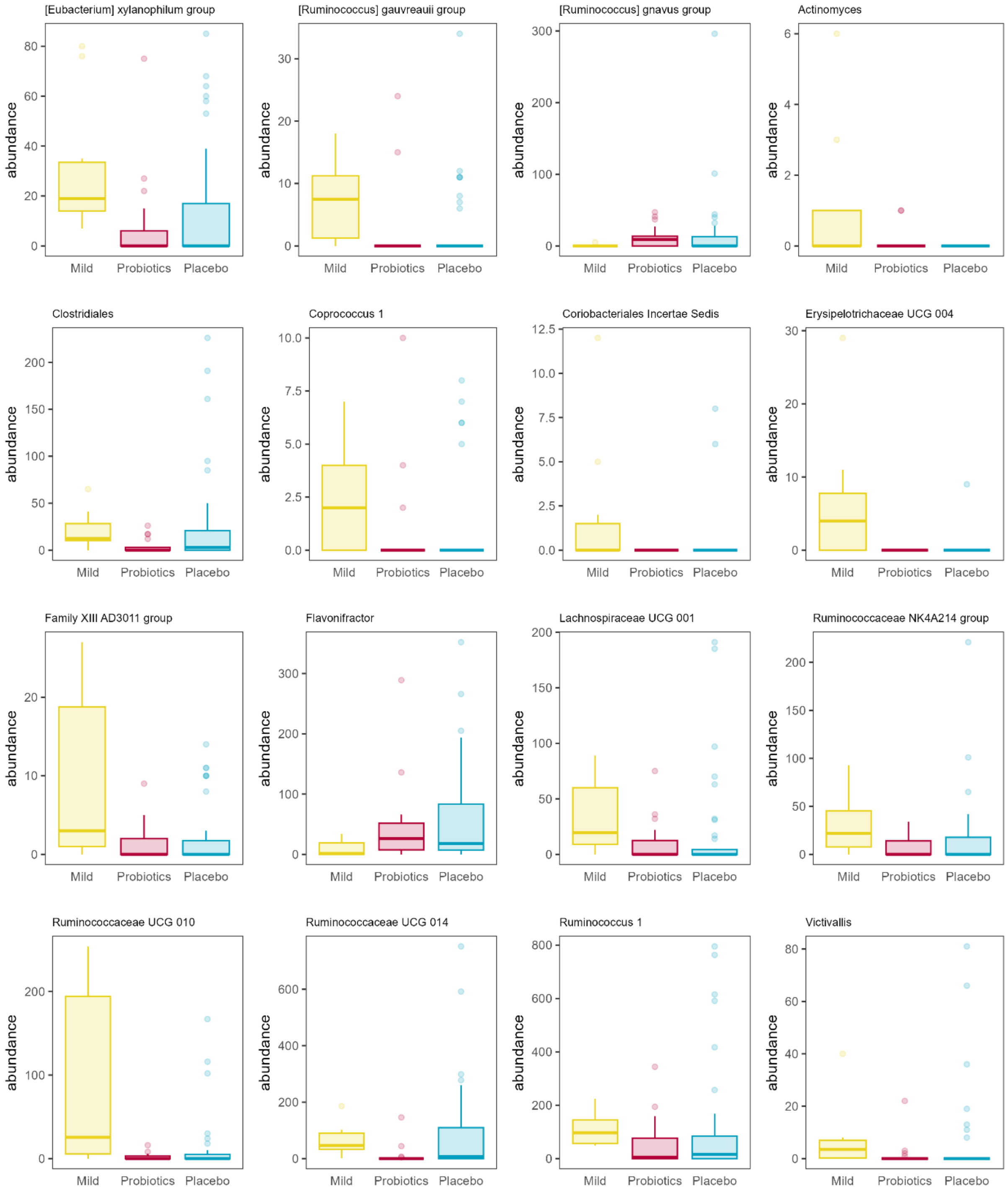


Figure S5: Differentially abundant genera at baseline assessed by MaAsLin2 comparing patients after severe COVID-19 disease to patients after mild COVID-19 disease (“Mild”). Patients after severe disease are shown according to their allocation in the intervention trial to show comparability of the microbiome before the intervention started (“Probiotics”/“Placebo”). All genera in this panel showed a q-value smaller than 0.05 after Benjamini Hochberg correction when comparing patients after mild to patients after severe disease.

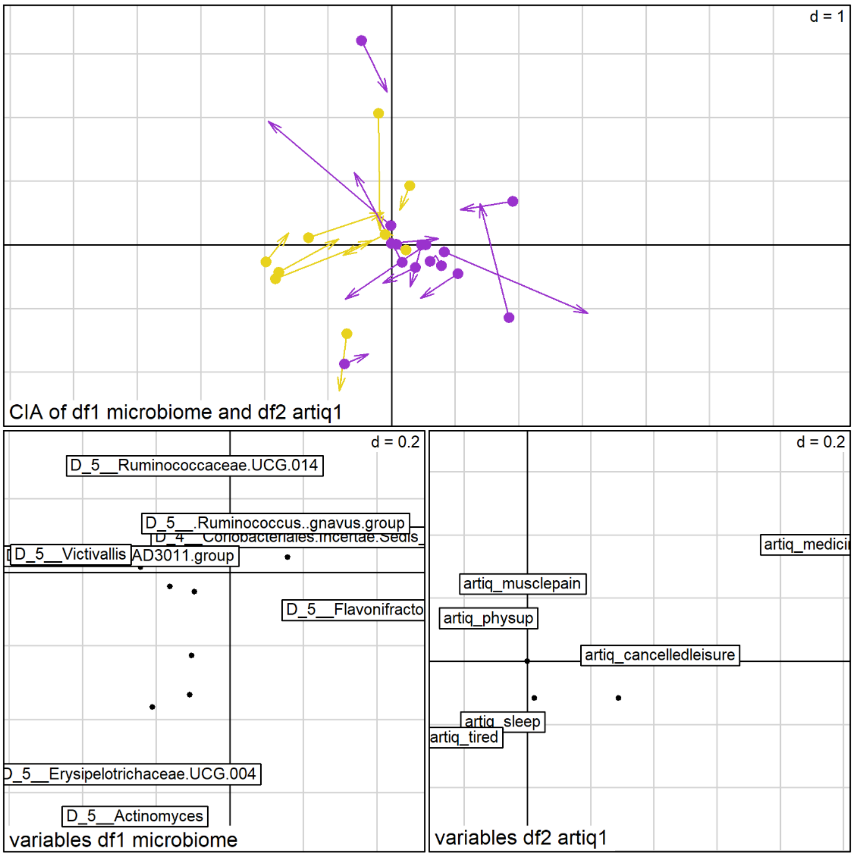


Figure S6: Co-inertia plot for differentially abundant genera and symptoms assessed with the ARTIQ questionnaire. In the sample space (top graph), patients after mild COVID-19 diseases are colored yellow, patients after severe disease are kept in purple.

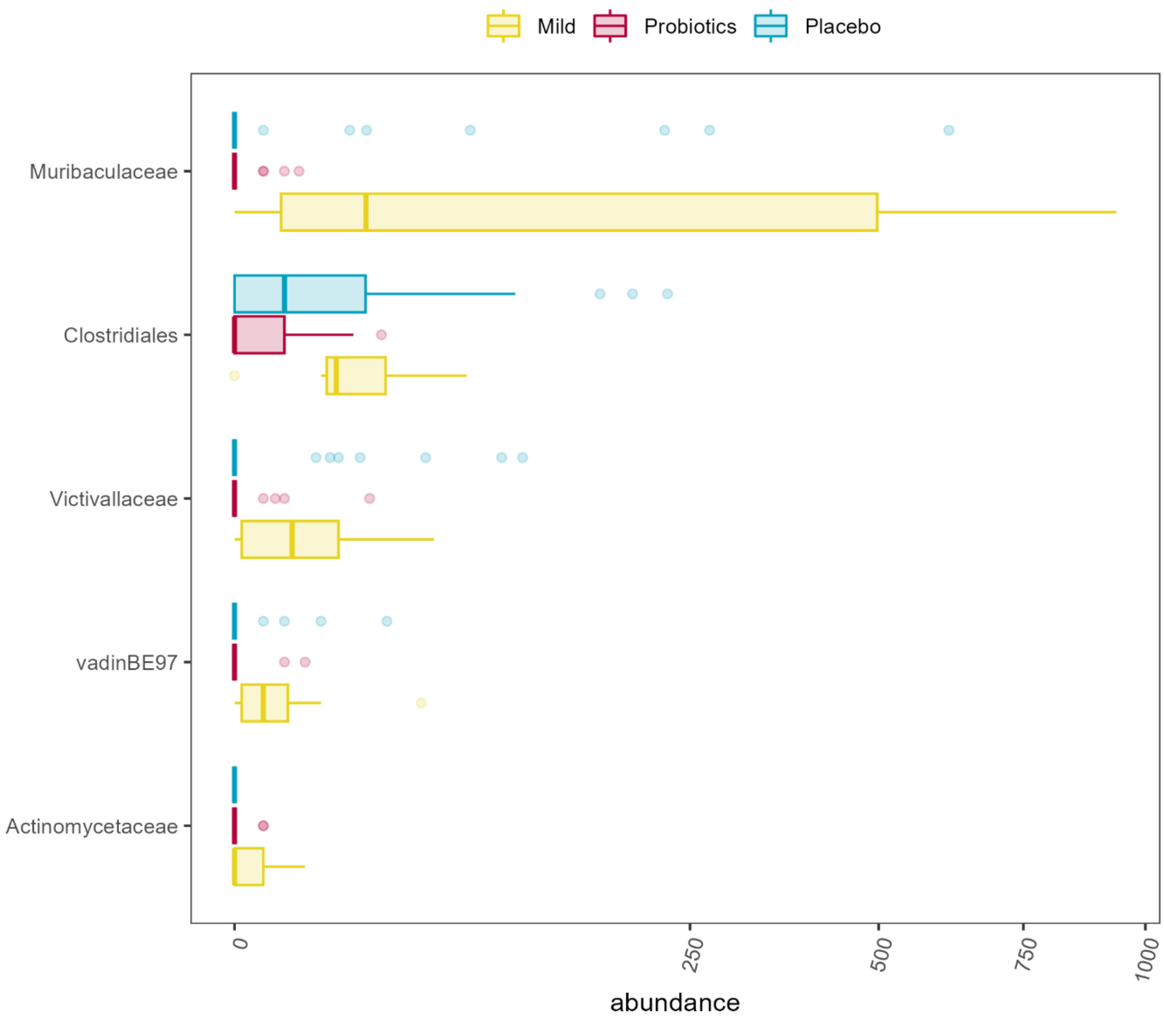


Figure S7: Differentially abundant families identified by MaAsLin2 in patients after severe COVID-19 infections (placebo, verum (probiotics)) compared to controls after mild COVID-19 infection.

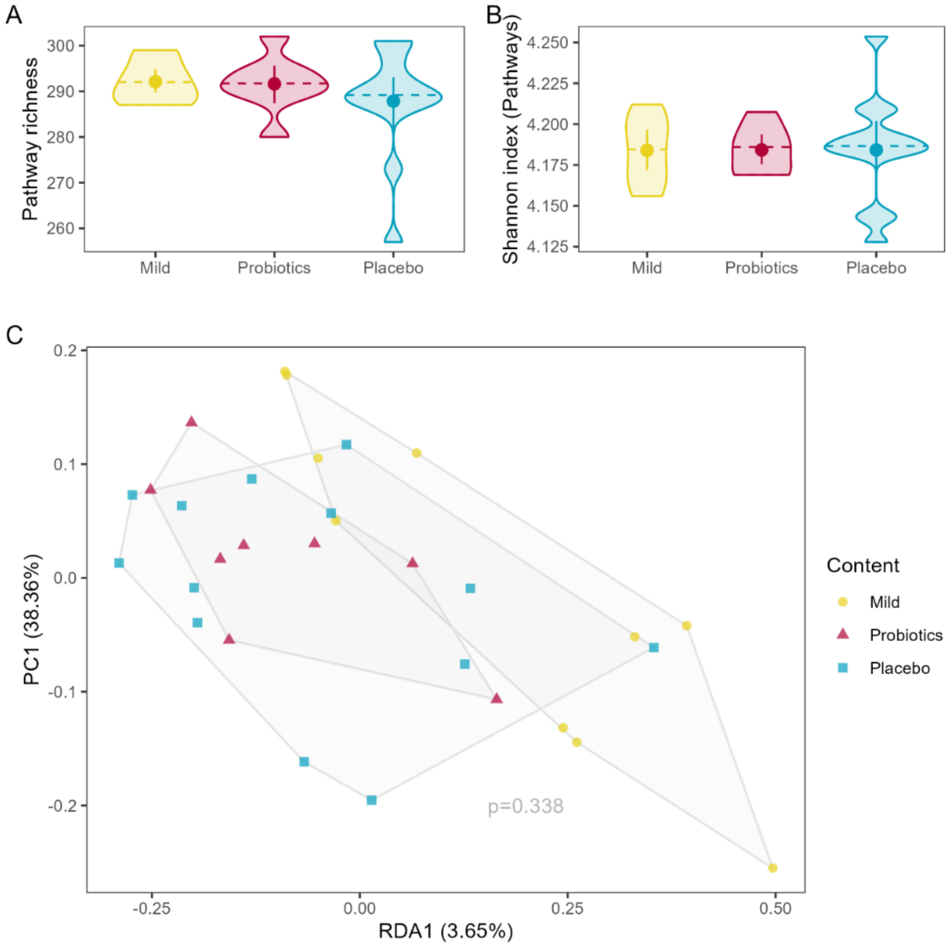


Figure S8: Pathway diversity of predicted metagenomics based on Tax4Fun analysis of the 16S rRNA gene sequencing of patients after severe COVID-19 infections (placebo, verum (probiotics)) and controls after mild COVID-19 infection. A+B. Alpha diversity of predicted pathways; B: Redundancy analysis of predicted pathway diversity.

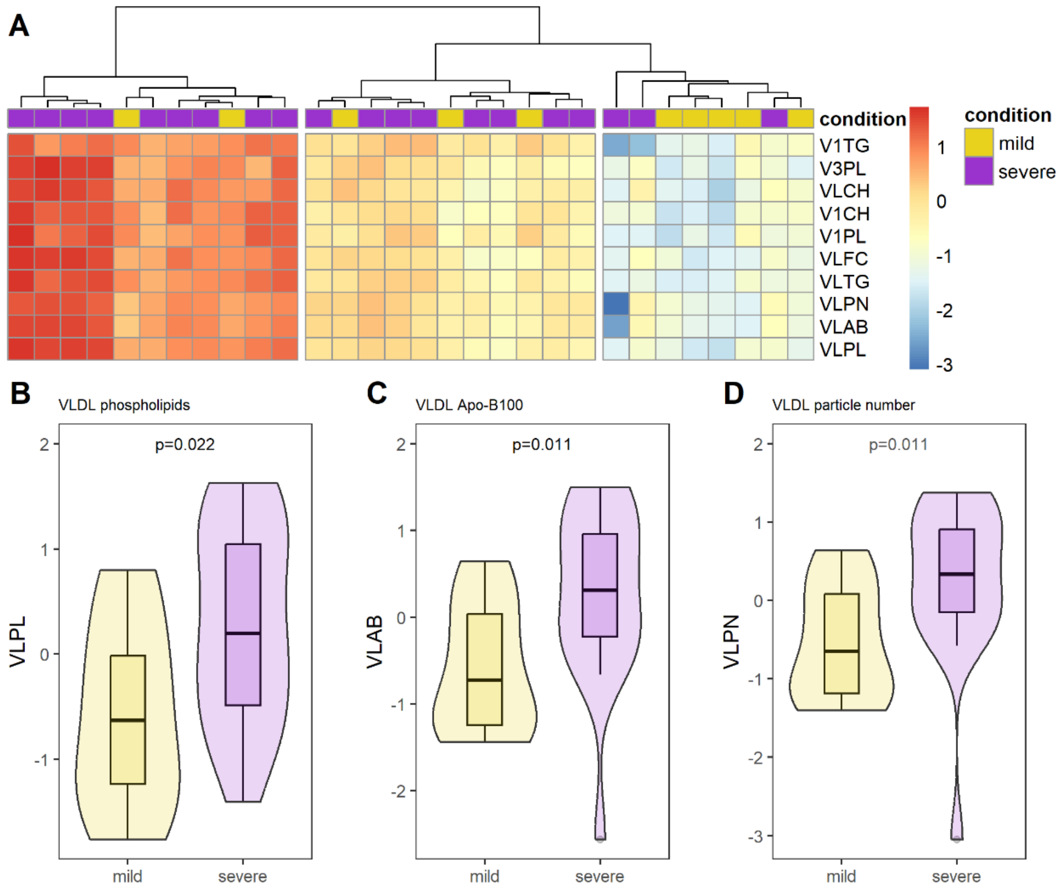


Figure S9: Multi-omics factor analysis; details for factor 2. A. Heatmap for the 10 serum metabolites contributing the highest weights to the factor with hierarchical clustering and group allocation for the examined samples. B-D: Normalized values for the top three contributors compared between patients after mild and severe disease. VLDL - very-low-density lipoprotein; VLPL  VLDL phospholipids; VLAB VLDL Apo B-100; VLPN - VLDL particle number; VLTG - VLDL triglycerides; VLFC - VLDL free cholesterol; V1PL - VLDL-1 phospholipids; V1CH - VLDL-1 cholesterol; VLCH – VLDL cholesterol; V3PL – VLDL-3 phospholipids; V1TG - VLDL-1 triglycerides.

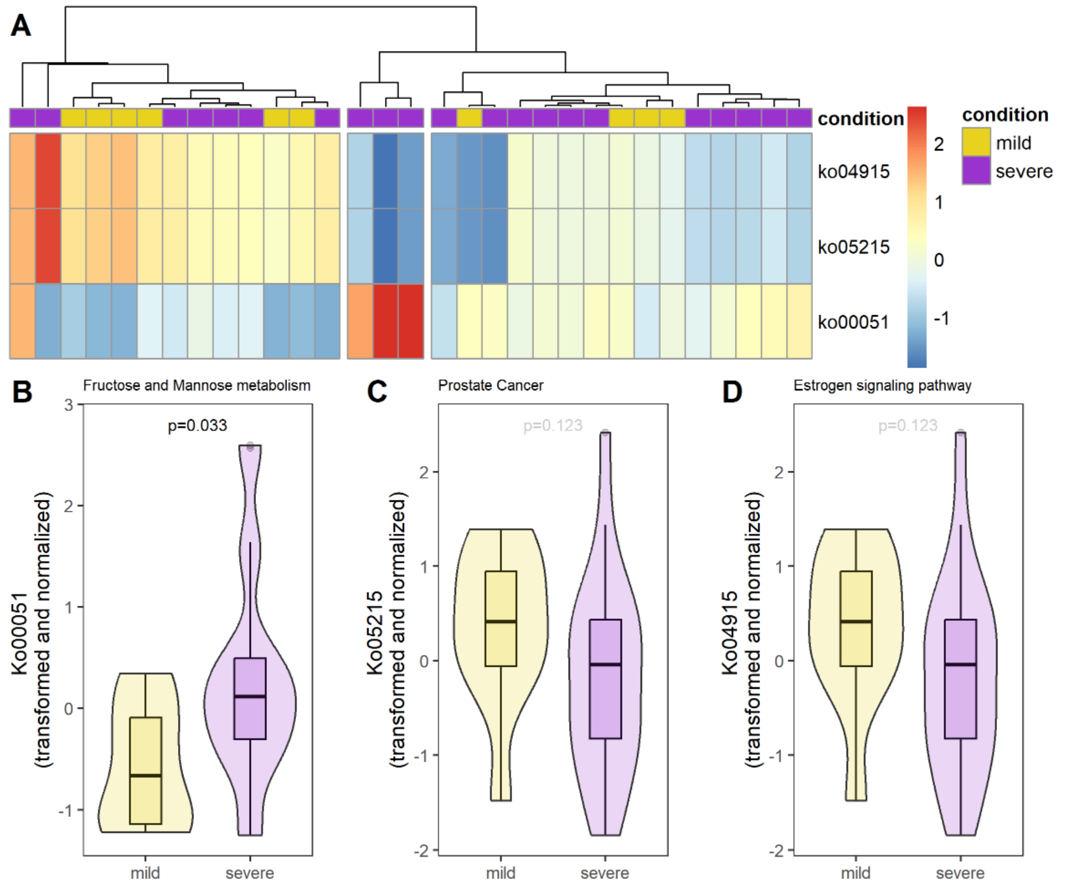


Figure S10: Multi-omics factor analysis; details for factor 5. A. Heatmap for the 3 predicted pathways contributing the highest weights to the factor with hierarchical clustering and group allocation of the examined samples. B-D: Normalized values for the contributors compared between patients after mild and severe COVID-19 disease; ko00051 - fructose and mannose metabolism pathway; ko05215 – prostate cancer; ko04915 – estrogen signaling pathway.

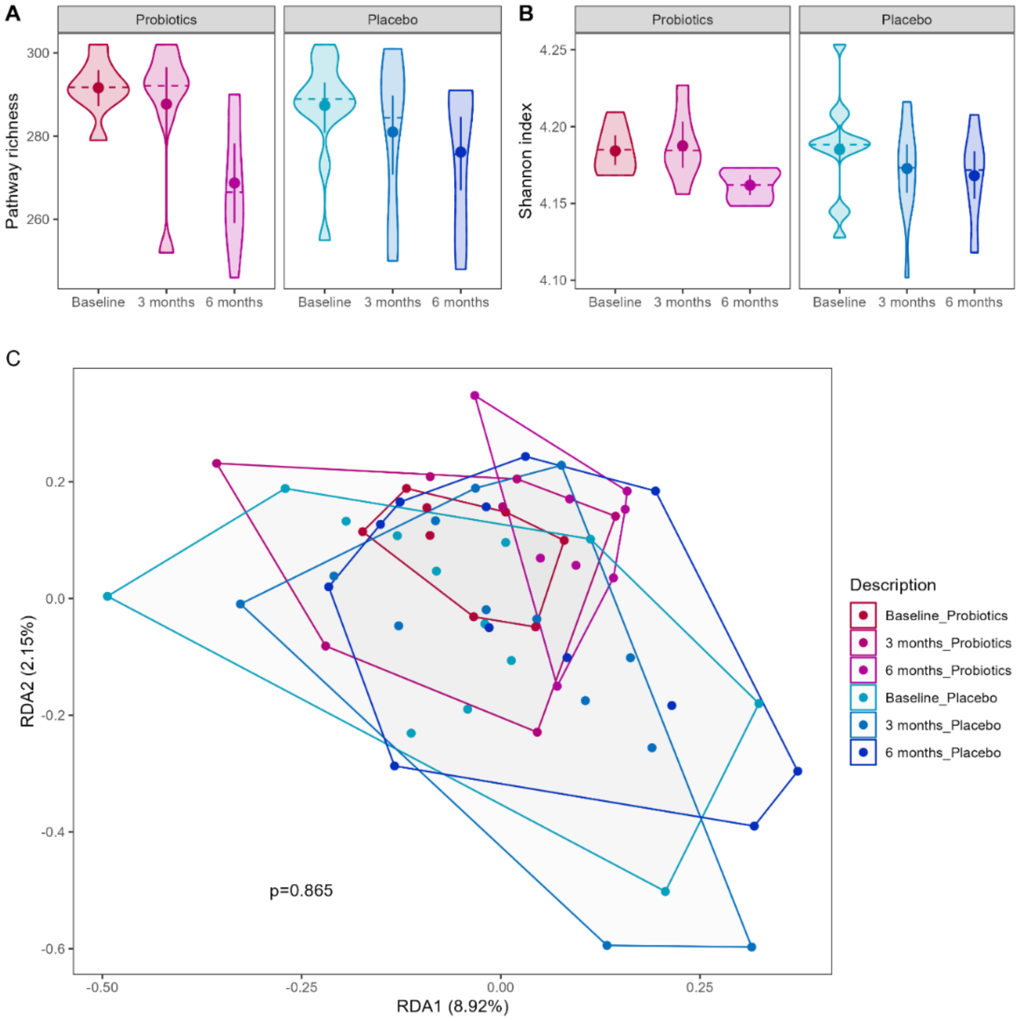


Figure S11: Pathway diversity of predicted metagenomics based on Tax4Fun analysis of the 16S rRNA gene sequencing in patients treated with Probiotics (I.e. Verum) or Placebo. A+B. Alpha diversity of predicted pathways; C. Redundancy analysis of predicted pathway diversity.

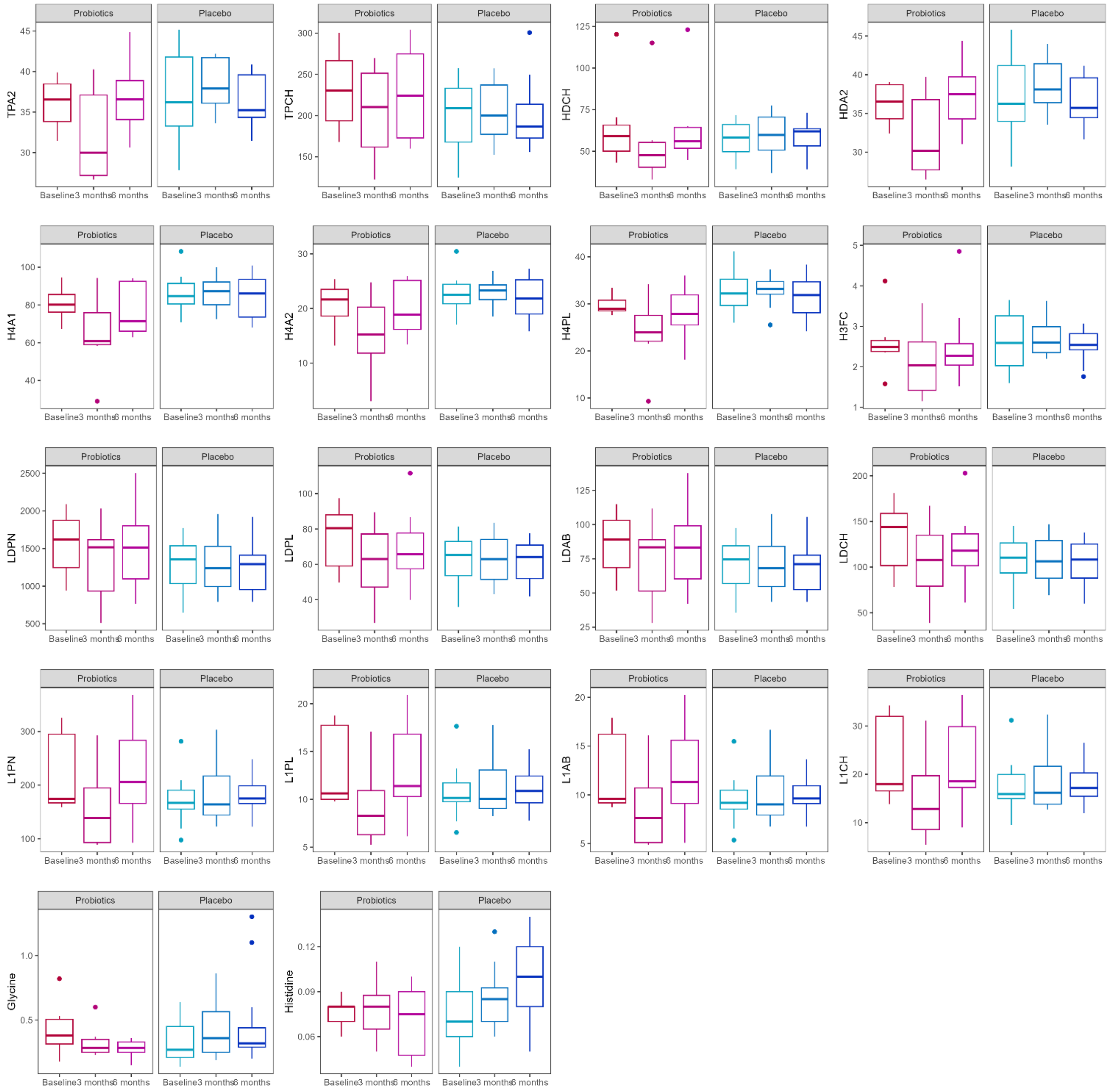


Figure S12: Serum lipoprotein parameters modulated by probiotic intervention. TPA2 - total Apo-A2, TPCH - total cholesterol, HDCH - HDL cholesterol, HDA2 - HDL Apo-A2, H4A1 - HDL-4 Apo-A1, H4A2 - HDL-4 Apo-A2, H4PL - HDL-4 phospholipids, H4CH - HDL-4 cholesterol, H4FC - HDL-4 free cholesterol, H3FC - HDL-3 free cholesterol, LDPN - LDL particle number, LDPL - LDL phospholipids, LDAB - LDL Apo-B100, LDCH - LDL cholesterol, LDFC - LDL free cholesterol, L1PN - LDL-1 particle number, L1PL - LDL-1 phospholipids, L1AB - LDL-1 Apo-B100, L1CH - LDL-1 cholesterol, L1FC - LDL-1 free cholesterol.

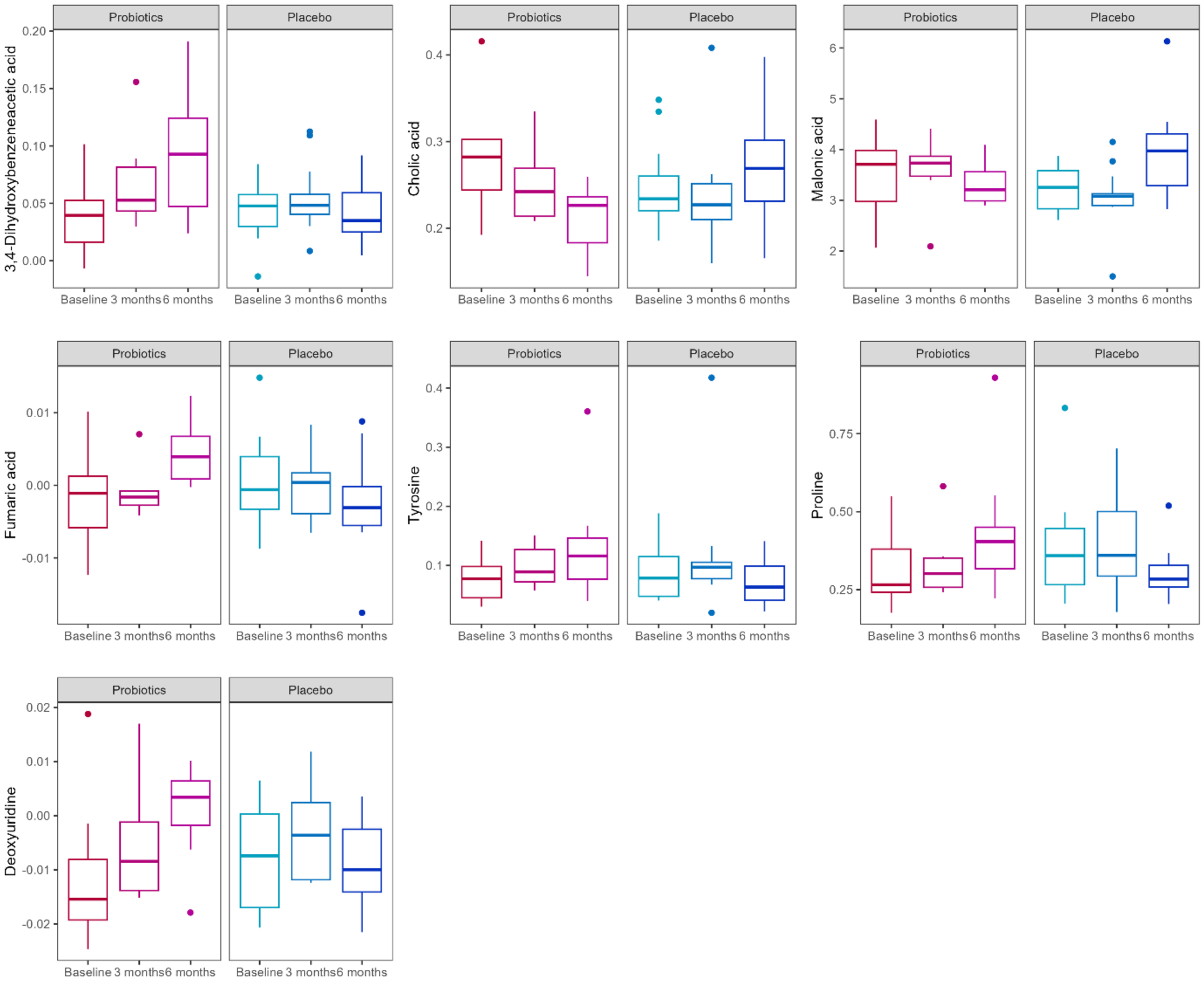


Figure S13: Stool metabolites modulated by probiotic interventions.

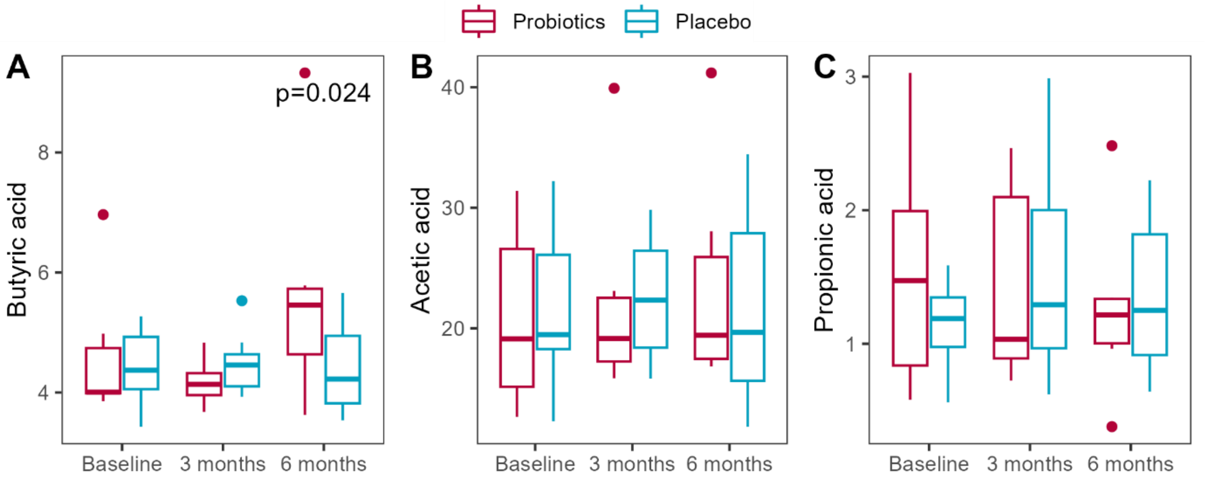


Figure S14: Short chain fatty acids in stool throughout the study period for patient in the probiotic and the placebo group.

1. Karmakar, U., et al., *Immune complex-induced apoptosis and concurrent immune complex clearance are anti-inflammatory neutrophil functions.* Cell Death Dis, 2021. **12**(4): p. 296.
